# Boolean regulatory network reconstruction using literature based knowledge with a genetic algorithm optimization method

**DOI:** 10.1186/s12859-016-1287-z

**Published:** 2016-10-06

**Authors:** Julien Dorier, Isaac Crespo, Anne Niknejad, Robin Liechti, Martin Ebeling, Ioannis Xenarios

**Affiliations:** 1 Vital-IT, Systems biology and medicine department, SIB Swiss Institute of Bioinformatics, 1015 Lausanne, Switzerland; 2 Pharmaceutical Sciences/Translational Technologies and Bioinformatics, Roche Innovation Center Basel, 124 Grenzacherstrasse, 4070 Basel, Switzerland

**Keywords:** Boolean regulatory networks, Qualitative modeling, Network inference, Optimization, Genetic algorithm, Prior knowledge network

## Abstract

**Background:**

Prior knowledge networks (PKNs) provide a framework for the development of computational biological models, including Boolean models of regulatory networks which are the focus of this work. PKNs are created by a painstaking process of literature curation, and generally describe all relevant regulatory interactions identified using a variety of experimental conditions and systems, such as specific cell types or tissues. Certain of these regulatory interactions may not occur in all biological contexts of interest, and their presence may dramatically change the dynamical behaviour of the resulting computational model, hindering the elucidation of the underlying mechanisms and reducing the usefulness of model predictions. Methods are therefore required to generate optimized contextual network models from generic PKNs.

**Results:**

We developed a new approach to generate and optimize Boolean networks, based on a given PKN. Using a genetic algorithm, a model network is built as a sub-network of the PKN and trained against experimental data to reproduce the experimentally observed behaviour in terms of attractors and the transitions that occur between them under specific perturbations. The resulting model network is therefore contextualized to the experimental conditions and constitutes a dynamical Boolean model closer to the observed biological process used to train the model than the original PKN. Such a model can then be interrogated to simulate response under perturbation, to detect stable states and their properties, to get insights into the underlying mechanisms and to generate new testable hypotheses.

**Conclusions:**

Generic PKNs attempt to synthesize knowledge of all interactions occurring in a biological process of interest, irrespective of the specific biological context. This limits their usefulness as a basis for the development of context-specific, predictive dynamical Boolean models. The optimization method presented in this article produces specific, contextualized models from generic PKNs. These contextualized models have improved utility for hypothesis generation and experimental design. The general applicability of this methodological approach makes it suitable for a variety of biological systems and of general interest for biological and medical research. Our method was implemented in the software optimusqual, available online at http://www.vital-it.ch/software/optimusqual/.

**Electronic supplementary material:**

The online version of this article (doi:10.1186/s12859-016-1287-z) contains supplementary material, which is available to authorized users.

## Background

High-throughput technologies in different areas of biomedical research provide massive amounts of data that are difficult to organize and interpret. Network-based representations of biological systems have become a popular way to structure and analyse this information. Networks can be inferred directly from experimental data or created from prior knowledge – Prior Knowledge Networks (PKN). Both strategies have their advantages and disadvantages. PKNs are normally not context-specific, as they usually include all regulatory interactions relevant to the process under study, and may be biased towards biological entities and interactions that are intensively studied, or merge interactions measured under different experimental conditions, including different cell types or tissues. Networks inferred exclusively from high-throughput data (such as transcriptomic or proteomic data) may be more comprehensive and more contextualized to the experimental conditions of interest, but they do not provide direct information about causal relationships, i.e., directionality, as they are usually based on the statistical co-occurrence of biological events (for example, co-expression of two genes). The underlying causality can be elucidated only for a reduced number of cases by costly perturbation experiments or time series data.

In an attempt to overcome the drawbacks of these two network construction strategies, several methods combining both prior knowledge and experimental data have been developed during the last years. Among these methods, we distinguish those that exhaustively explore the search space in order to identify an optimal configuration that explains the experimental data - combinatorial optimization methods - from those based on a heuristic approach. Combinatorial optimization methods include those based on integer programming [1–4] or answer set programming [5]. Their computational complexity grows exponentially with the network size, limiting their applicability to small systems. Heuristic approaches that attempt to overcome these limitations can be divided into those that focus on describing the response of the system to perturbations and those that focus on describing the stable states of the system. Heuristic contextualization methods that focus on describing the response of the system to perturbations require multiple perturbation experiments to train the PKN. Saez-Rodriguez et al. [6] proposed discrete logic modelling to curate and expand canonical signalling pathways using information from perturbation experiments to train the model. Irit Gat-Viks et al. [7] developed a similar method to construct discrete dynamical models from a PKN that include feedback loops, transforming the original graph into multiple acyclic graphs starting from multiple perturbed nodes, for which perturbation experiment data should be available.

Heuristic contextualization methods that focus on describing the stable states of the system require less experimental information but the resulting models are strongly contextualized to the stable states. Examples include the methods proposed by Layek et al. [8], Crespo et al. [9] and Rodriguez et al. [10]. The main limitation of these methods is that, given that they are trained to describe correctly the stable states, they may fail to describe other dynamical behaviours, such as the transitions between stable states under specific perturbations. In a specific example, Rodriguez et al. [10] showed in a model of the T-helper differentiation process how only a fraction of the alternative optimized models successfully described a known transdifferentiation between phenotypes Th1 and Th2 under the stimulation of GATA3.

Here we propose a new heuristic method for the contextualization of PKNs that specifically addresses the above-mentioned limitations. It consists in a heuristic network training approach that considers not only the stable states of the system but also the reachability of those states under specific perturbations. The method takes as input a PKN and experimental information about the stable states and transitions between them upon perturbation. A genetic algorithm is used to explore the search space of Boolean networks with asynchronous updates and to find networks that best describe the experimental data, using only edges present in the PKN. We demonstrate the ability of our algorithm to reconstruct a previously published cell-fate decision model [11] used as gold standard, by comparing the networks reconstructed by our algorithm to the original cell-fate decision model. The results demonstrate the utility of the approach to reconstruct reliable dynamical Boolean models based on the integration of PKN and experimental data. Such models can be interrogated to predict network response under perturbation, stability properties and robustness of the network, with potential application to guide experimental approaches including hypothesis generation.

## Methods

**Fig. 1:**
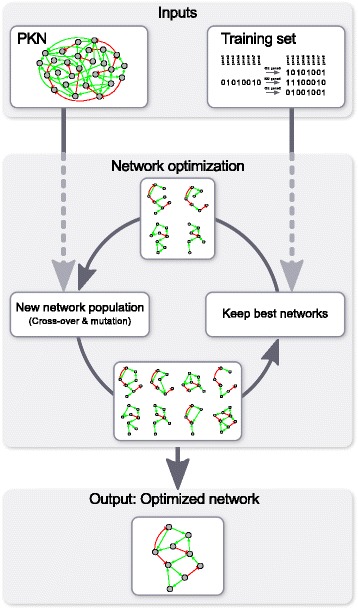
Optimization method. Our network optimization method takes as input a PKN and a training set and uses a genetic algorithm to find sub-graphs of the PKN which reproduce as well as possible all experiments in the training set. For each run of the optimization method, only the best network is kept as optimized network. To increase the chances to obtain good solutions, multiple independent runs of the optimization method are usually started in parallel, and only a fraction of optimized networks is kept as model networks

This section is divided in two main parts. Part 1 describes our network optimization method (Fig. 1). This takes as input a PKN and a set of experiments (training set) and uses a genetic algorithm to train model networks to reproduce as closely as possible the experimental data provided in the training set, with the constraint that all edges must be taken from the PKN. The output of the method is a set of model networks. Part 2 describes our method to assess the quality of network optimization. This uses an *in silico* gold standard network to generate PKNs and training sets, which are taken as input for the optimization method. The resulting model networks are then compared to the original *in silico* gold standard network and the result of this comparison is taken as a measure of our network optimization method quality.

### Description of the network optimization method

#### Definitions

##### Model network

**Fig. 2:**
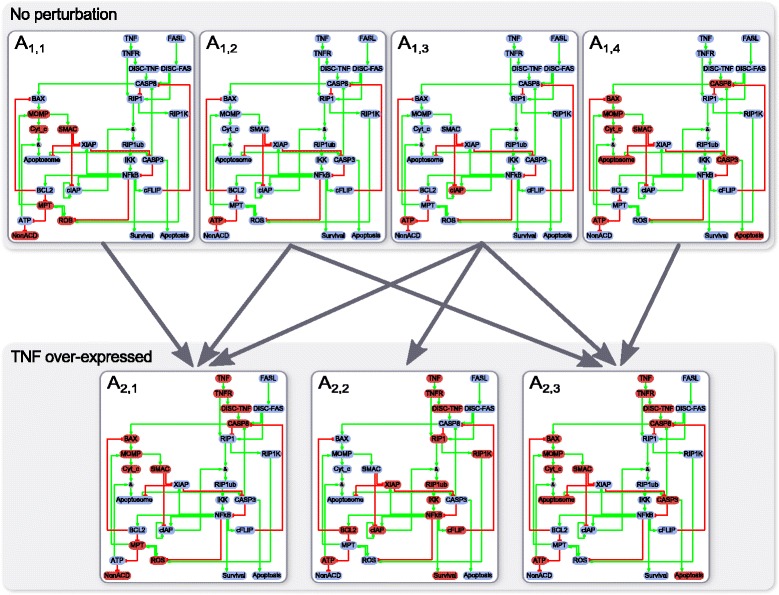
In silico experiments and attractor reachability graph. Example of attractor reachability graph for the transition from unperturbed network to over-expression of TNF on the cell-fate decision model [11]. The unperturbed network has four attractors, each shown as a network with *red nodes* corresponding to active nodes and *blue nodes* corresponding to inactive nodes. These attractors are labelled A_1,1_, A_1,2_, A_1,3_, and A_1,4_. Attractor A_1,3_ corresponds to the physiological state used by Calzone and co-authors [11] as initial state. The perturbed network (TNF fixed to 1) has only three attractors (denoted A_2,1_, A_2,2_ and A_2,3_), interpreted as the three cell fates: non-apoptotic cell death, survival, and apoptosis. After TNF over-expression, each attractor of the unperturbed network will stabilize into at least one attractor of the perturbed network (*grey arrows*). For example, the physiological state A_1,3_ will stabilize into either of the three cell fates A_2,1_, A_2,2_ and A_2,3_. Note that this figure presents only the transition from unperturbed network to over-expression of TNF, and not the reverse transition. Therefore no information is shown on the reachability from attractors obtained with over-expression of TNF to attractors of the unperturbed network

A model network is a Boolean network, used to model a given biological process. Ideally, the model network obtained after the optimization procedure should behave like the biological system. In this work we consider asynchronous Boolean networks, as defined by Garg and co-authors [12]. Each node corresponds to a gene or a protein and its state is given by a Boolean variable, which can represent node expression or activity. Edges correspond to interactions between nodes and can be positive (activators) or negative (inhibitors). The dynamical behaviour of a Boolean network can be measured by performing *in silico* experiments. In this work, an *in silico* experiment consists of a set of perturbations (over-expression/knock-out of one or any combination of nodes) and a set of transitions between them. For each transition from a perturbation P_1_ to a perturbation P_2_, the output of the *in silico* experiment is an attractor reachability graph (see Fig. 2) whose nodes are attractors obtained with each perturbation and edges denote reachability between attractors. More precisely, an edge will connect an attractor obtained with perturbation P_1_ to an attractor obtained with perturbation P_2_ if and only if the states of the first attractor are connected to the states of the second attractor by at least one path in the asynchronous state transition graph of the network with perturbation P_2_.

To perform *in silico* experiments, two methods were used in this work. The first method is boolSim, a software developed by Garg and co-authors [12], which uses an implicit method based on reduced ordered binary decision diagrams to evaluate the attractor reachability graph of a network. This method is exact and exhaustively finds all attractors but quickly becomes too computationally expensive for large networks. The second method we used was a simple algorithm based on a stochastic exploration of the state transition graph (see Additional file 1 for details). This stochastic approach scales better than boolSim as network size increases, but there is no guarantee that all attractors will be found. In this work, the stochastic method was used to evaluate attractor reachability graphs in our implementation of the optimization method, while boolSim was used to assess the quality of the final optimization results.

##### Prior knowledge network (PKN)

The PKN is a network that summarizes known interactions between genes and/or proteins of interest, usually obtained from the literature by a biocurator or by automatic text mining methods. Note that although this network can contain direct interactions, most of the interactions are usually indirect.

##### Training set

The training set is a set of known experimental results that the final model network should be able to reproduce. In this work, the training set consists of stable phenotypes measured in different conditions and transitions between them. It is given in the form of a transition graph. Each node of the training set graph is defined by a perturbation and an observation. A perturbation can involve the over-expression (node always set to 1) and/or knock-out (node always set to 0) of any subset of nodes in the network. An observation is a list of nodes and their corresponding state measured at equilibrium after the perturbation. Node states should be either 0 or 1. Edges in the training set graph correspond to transitions between stable phenotypes. An edge from perturbation P_1_ with observation O_1_ to perturbation P_2_ with observation O_2_ means that under perturbation P_1_ the system exhibits a stable phenotype characterized by observation O_1_ and after perturbation P_2_ the system will stabilize into a phenotype characterized by observation O_2_. Note that P_1_ = P_2_ is allowed, as well as O_1_ = O_2_.

Intuitively, a Boolean network reproduces a training set if for each perturbation/observation in the training set, the network can stabilize into an attractor compatible with the observation when applying the corresponding perturbation. In addition, attractors matched to perturbation/observation nodes linked by an edge in the training set graph should be connected by “time” evolution of the network, i.e. connected by an edge in the attractor reachability graph. Finally, each node of the training set graph should correspond to a unique attractor in the attractor reachability graph. For instance, the following training set graph (P_1_,O_1_) → (P_2_,O_2_) → (P_3_,O_3_) means that the model network should have at least one attractor A_1_ with node states corresponding to observation O_1_ when perturbed by P_1_, as well as attractors A_2_ and A_3_ with node states corresponding to observations O_2_ and O_3_ when perturbed by P_2_ and P_3_ respectively. In addition, A_1_ should be connected to A_2_ and the same attractor A_2_ should be connected to A_3_ in the attractor reachability graph.

This definition of training set is flexible enough to accommodate complex experimental scenarios such as the (long term) responses to drugs in specific mutational backgrounds, but also time-dependent processes such as cellular differentiation in response to various combinations of stimuli. Multiple scenarios can even be combined into a unique training set (e.g. training set 1 in Additional file 2).

#### Problem formulation

Given a PKN and a training set, find the model network that reproduces as well as possible all experiments in the training set, under the constraint that the model network must be a sub-graph of the PKN (all edges and nodes in the model network must exist in the PKN).

We also enforced the following properties of the model network, given in decreasing order of importance. (i) It should include a user-defined set of essential nodes. Typically, this set will contain nodes that are known to play an important role in the modelled biological process, or that represent key experimental readouts. (ii) It should be as small as possible in terms of number of nodes. Since the prior knowledge network can be very large, it can be challenging to evaluate its attractors, therefore having a model network that is as simple as possible should reduce the computational effort. (iii) Given a set of nodes, the model network should contain as many edges (connecting these nodes) from the PKN as possible. The idea here is to force the model network to be as close as possible to the PKN, removing only edges that are in contradiction with the training set.

To optimize all these properties simultaneously, we used a standard multi-objective optimization approach, based on a multi-dimensional fitness function, defined for each network as

$$ \mathbf{F}=\left({f}_T,-{N}_{ess. nodes},{N}_{nodes},-{N}_{edges}\right) $$

where *f*_*T*_ measures how well the network reproduce the experiments given in the training set, *N*_*ess. nodes*_ is the number of essential nodes appearing in the network, *N*_*nodes*_ is its number of nodes and *N*_*edges*_ its number of edges. Multi-dimensional fitness functions are compared using lexicographical ordering, which means that optimization of *f*_*T*_ is considered as most important, followed by *N*_*ess. nodes*_, *N*_*nodes*_ and finally *N*_*edges*_. Note that the component responsible for maximization of the number of edges (*N*_*edges*_) appears only after the number of nodes (*N*_*nodes*_). As a consequence, networks with same *f*_*T*_ and *N*_*ess. nodes*_ are first compared according to their number of nodes. Only if they have the same number of nodes, their number of edges is taken into account in the comparison. The first component *f*_*T*_ (defined in Additional file 1) measures the average distance between observations contained in the training set and attractors of the network, with the following constraints on the chosen attractors: (1) if two observations are connected by an edge in the training set graph then the corresponding attractors must be connected in the attractor reachability graph and (2) each observation in the training set graph must correspond to a unique attractor. Among all attractors satisfying these two constraints, only the ones that minimize *f*_*T*_ are taken into account. Approximately, *f*_*T*_ can be considered as the fraction of observations in the training set that are not reproduced correctly by the network.

With these definitions, the challenge becomes a non-linear discrete optimization problem: the model network corresponds to the sub-graph of the PKN that minimizes the multi-dimensional fitness function **F**. For a PKN with *M* edges, the number of possible model networks is 2^*M*^. As a consequence, except for a very small PKN, brute force testing of all possible networks is not possible, and a numerical optimization method is required. Here we use a genetic algorithm (more details are available in Additional file 1), a well-established heuristic optimization method, which can be easily implemented and is known to give reasonably good results with non-linear discrete optimization problems. Heuristic optimization algorithms, like the genetic algorithm, are designed to seek good solutions, at a reasonable computational cost, but without guarantee of optimality or completeness. It is worth noting that this problem is neither linear nor convex, and therefore more efficient linear or convex optimization methods cannot be used here. Indeed, contrary to the case where the training set contains only information on steady states (attractors with only one state) or successive states in the state transition graph, which can be reformulated as a linear or convex optimization problem [3, 4], the more general form of our training set, which contains information on attractors but also reachability between them, prevents this reformulation.

Except for very large training sets, this optimization problem will in general not be completely specified. Indeed, several different networks may be able to reproduce the training set equally well (minimize *f*_*T*_). Although adding more components to the fitness function (*N*_*ess. nodes*_, *N*_*nodes*_ and *N*_*edges*_) should help to reduce the number of networks that are solutions of the optimization problem, it may not be sufficient to reduce the solution to a unique network. Therefore, the solution to the optimization problem should not be considered as a unique network, but as a collection of different networks which are all equally good candidate models. This is not a limitation of the chosen optimization method (genetic algorithm), but a consequence of the lack of knowledge on the biological system. To reduce the number of solutions, an obvious solution consists in increasing the training set size. Alternatively, carefully building the PKN without unnecessary edges will help to decrease the dimension of the search space as well as the number of solutions.

The bottleneck with this approach in terms of performance is the evaluation of attractor reachability graphs. As a consequence, this method should be used with networks having on the order of hundreds of nodes. This limit on the network size is not strict, since the computational cost will depend on the number of nodes, connectivity and topology of the optimized networks, which are governed by the corresponding properties of the PKN as well as the number of essential nodes.

### Evaluation of the network optimization method

To evaluate the network optimization method, we used a gold standard model to generate *in silico* PKNs and training sets, which were then used as input for our network optimization method. The resulting model networks were then compared to the original gold standard network, and the result of this comparison was taken as a proxy for the quality of the optimization method.

**Fig. 3:**
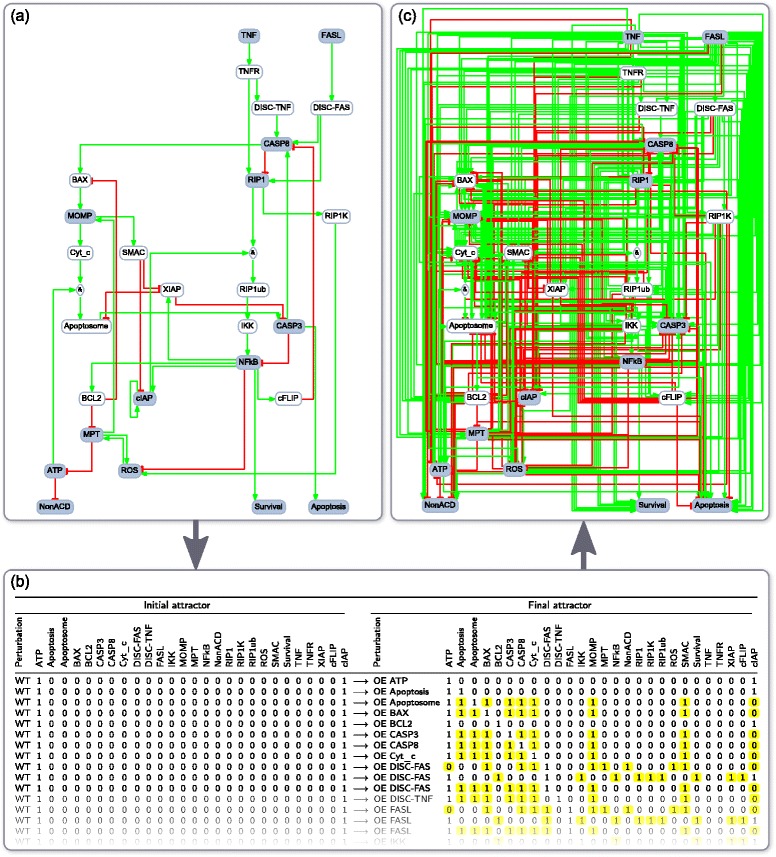
Cell-fate decision model. **a** Cell-fate decision model [11] used as gold standard. Essential nodes are shown with *grey* background. **b** List of transitions, starting from each of the 4 unperturbed network’s attractors to attractors reached after single node perturbations of the cell-fate decision model. Each line corresponds to an edge in the attractor reachability graph. Only the first lines are shown, the full table is given in Additional file 3. **c** PKN obtained from (**b**) by adding edges from perturbed nodes to observed nodes whose state (absolute value) change by more than 0.5 (highlighted in *yellow*). Edges are positive if perturbation and variation of observed node state have same sign, and negative if they have opposite sign. For example, line 3 in table (**b**) will generate seven positive edges from Apoptosome to Apoptosis, BAX, CASP3, CASP8, Cyt_c, MOMP and SMAC as well as one negative edge from Apoptosome to cIAP. The complete list of interactions in the PKN is given in Additional file 4 (PKN 1)

As gold standard, we used a cell-fate decision model proposed by Calzone and co-authors [11] (Fig. 3a). Note that it is outside the scope of the present paper to discuss whether this model correctly describes the underlying biological process. A toy model completely unrelated to any biological process could also have been used as a gold standard.

To illustrate the simplification of the network obtained by minimizing the number of nodes, we decided to use training sets containing information for only a fraction of all nodes in the gold standard model, namely the 14 nodes chosen by Calzone and co-authors for their reduced cell-fate decision model [11] (grey nodes in Fig. 3a).

#### Generating input data

##### In silico PKN

BoolSim was used to perform every possible single node perturbation experiment on the gold standard network, starting from the unperturbed network. The resulting attractor reachability graphs were used to build a list of attractor transitions (see Fig. 3b and Additional file 3), with each line corresponding to one edge in the attractor reachability graph. If an attractor had more than one state, it was replaced by the average of all its states. Assuming that each line in the list transitions (Fig. 3b and Additional file 3) corresponded to a transition that could be observed experimentally and reported in an article, an *in silico* PKN was built by linking perturbed nodes to observed nodes whose states changed significantly (Fig. 3c and PKN 1 in Additional file 4). That is, each line in Fig. 3b generates edges from the perturbed node to each observed node whose state changes by more than 0.5 (absolute value). Edges are positive if perturbation and variation of observed node state have the same sign, and negative if they have opposite signs. Finally all edges of the gold standard network also are added to the PKN.

In addition to this ideal PKN, we also generated five PKNs with increasing fractions of noise (10, 20, 30, 40 and 50 % noise). To generate a PKN with noise fraction *q*, we randomly replaced a fraction *q* of all edges in the ideal PKN by the same number of edges randomly chosen in the set of edges of the form *n*_1_ → *n*_2_ and *n*_1_ ⊣ *n*_2_ that were not in the ideal PKN (*n*_1_, *n*_2_ are nodes of the ideal PKN). The lists of interactions in each of these PKNs are given in Additional file 4 (PKN 2 to 6).

##### Essential nodes

We used the 14 nodes of the reduced cell-fate decision model proposed by Calzone and co-authors [11]: ATP, Apoptosis, CASP3, CASP8, cIAP, FASL, MOMP, MPT, NFkB, NonACD, RIP1, ROS, Survival and TNF (grey nodes in Fig. 3a; i.e., all nodes for which experimental data was assumed to be available).

##### In silico training sets

We built a training set based on the response to TNF and FASL perturbations by mutant versions of the cell-fate decision model discussed by Calzone and co-authors [11]. We considered seven mutant versions: wild-type, CASP3 knock-out, CASP8 knock-out, cIAP knock-out, NFkB knock-out, RIP1 knock-out and simultaneous knock-out of CASP3, CASP8 and RIP1. For each mutant, we measured the response of the gold standard network to TNF over-expression, FASL over-expression and simultaneous over-expression of TNF and FASL, starting from the physiological state (all nodes inactive except ATP and cIAP) described by Calzone and co-authors [11]. For each attractor obtained, an observation was added to the training set, using the measured states of all essential nodes in the attractor. For each attractor reached after perturbation of TNF, FASL or TNF and FASL together, we added to the training set a transition from the initial attractor (physiological state) to the reached attractor. The resulting training set is given in Additional file 2, training set 1. Note that for a given mutant, all perturbations are linked to the same initial observation obtained with the unperturbed network, which means that during the optimization, only transitions starting from the same initial attractor will be used to evaluate the fitness function.

In addition to this training set, we also built two smaller training sets obtained by keeping only transitions for the wild-type mutant (training set 2 in Additional file 2), and keeping only the initial physiological attractor (training set 3 in Additional file 2).

To study the effect of errors in training sets on the optimization method, we also generated five training sets with increasing fractions of errors (10, 20, 30, 40 and 50 %) by randomly reversing the corresponding fraction of all node states appearing in training set 1 (training sets 1 with 10 to 50 % error in Additional file 2).

#### Comparing a model network to the gold standard

Given a model network, obtained by using the network optimization method with an *in silico* PKN and training set generated from the gold standard network, we measured how close this model network was to the gold standard network. Various metrics could be used here, but we considered that the most important characteristic of a model network was its ability to correctly predict the behaviour of the underlying biological system (in the present case, the gold standard model). To compare predictions from the model network and gold standard network we defined a score (denoted *s*_*all*_) which measures the similarity between the average states reached by the model network and gold standard network after all possible single node perturbations of essential nodes, starting from each attractor of the unperturbed gold standard network. More precisely, for each perturbation and initial state, the average state reached by a network is a vector of dimension *N*_*ess. nodes*_ whose n-th component is obtained as the average state of the n-th essential node over all attractors reached after the perturbation, starting from the given initial state. The *s*_*all*_ score is then defined as *s*_*all*_ = 1 − *Δ*/*N*_*ess. nodes*_, where *Δ* denotes the average over all perturbations and initial states of the Manhattan distance between average states reached by the model network and gold standard network (for a detailed definition see Additional file 1). We decided to consider not only the best attractor (as during the optimization process) but to consider all attractors reached, thus penalizing those situations where only part of the attractors reached by the model correspond to the attractors reached by the gold standard network.

The *s*_*all*_ score defined in this way has a value of 1 when the average predictions of both networks consistently agree for all perturbations and initial states, and a value of 0 when both networks consistently predict opposite states. Since the evaluation of the *s*_*all*_ score is based on experiments that are not part of the training set, it can be interpreted as a measure of the predictive power of the model network.

#### Evaluation of the optimization method: workflow

**Fig. 4:**
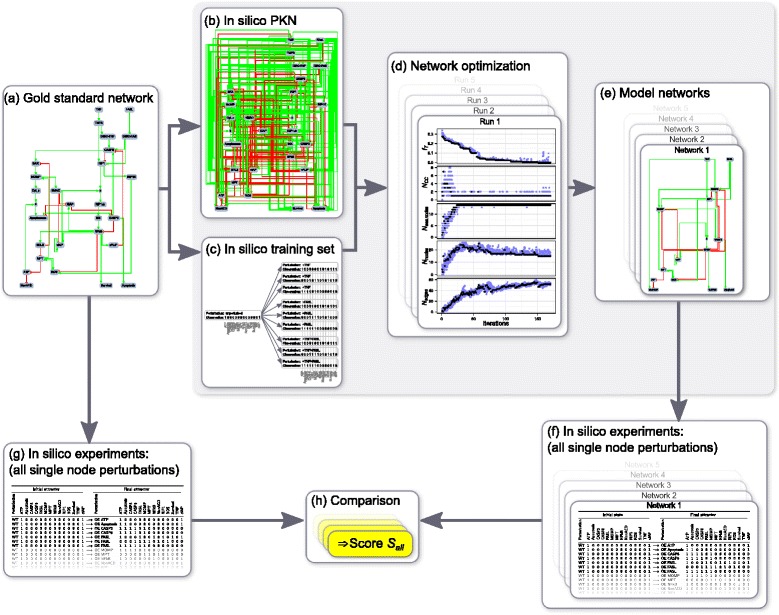
Evaluation of the optimization method: Workflow. To evaluate our optimization method, we used a gold standard network, interpreted as the true underlying biological system’s network (**a**). An *in silico* PKN (**b**) and a training set (**c**) containing a limited amount of information were generated by performing *in silico* experiments on the gold standard network. Using the PKN and training set as input, we started 500 independent runs of our network optimization (**d**), and kept the best network (with minimal value of the fitness function) obtained with each run. Among these 500 networks, the 50 best networks (according to fitness function) were kept as model networks (**e**). The predictive power of each model network was then evaluated by comparing its average predictions for all single node perturbations (**f**) to the corresponding gold standard network predictions (**g**). The resulting *s*_*all*_ scores (**h**) measure how close model networks and gold standard network predictions are and were taken as a proxy for the quality of the optimization method

An important question in the context of network optimization is whether a network is able to correctly predict the outcome of experiments that are not part of the training set against which it was optimized. To answer this question and to assess the quality of the network optimization method, we proceeded in the following way (see Fig. 4):

1. As described above, the gold standard network (Fig. 4a) was used to generate an *in silico* PKN (Fig. 4b) and an *in silico* training set (Fig. 4c).
2. Multiple independent runs (500 if not otherwise specified) of the network optimization method were performed, each with the same *in silico* PKN and training set as input (Fig. 4d), using a population of 50 replicas for the genetic algorithm. Each run was halted if the best value of the fitness function did not decrease during more than 10 iterations, and the network with the best fitness function obtained during the run was retained. Among the 500 networks generated in this way only the best 50 networks (according to fitness function) were kept as model networks (Fig. 4e).
3. For each model network and the gold standard network we performed *in silico* experiments to find the attractors reached after all possible single node perturbations of essential nodes, starting from each attractor of the unperturbed gold standard network (Fig. 4f and g).
4. The predictions for each model network were then compared to the gold standard network predictions by measuring the *s*_*all*_ score defined previously (Fig. 4h). These scores, which measure the predictive power of the model networks, were then interpreted as a measure of the optimization method quality.

This procedure was repeated with different training sets and PKNs to study the behaviour of the network optimization method in different conditions.

#### Random networks

The distribution of *s*_*all*_ scores indicates how close the model networks predictions are to the gold standard network predictions. Another important question is whether a score *s*_*all*_ obtained with a given model network is significantly better than a score obtained with a random network, i.e. is the optimization method better than a random network generator? To answer this question, random sub-networks of the PKN were generated by randomly keeping (with probability 0.5) each interaction in the PKN. The randomized network *s*_*all*_ scores were then evaluated and compared to the model network *s*_*all*_ score.

## Results and discussion

### Network optimization: applied example

We illustrate how our optimization method behaves by following its evolution when applied on a sample data set. We performed 500 independent runs of the optimization method using training set 1 (Additional file 2), PKN 1 (Additional file 4) and the 14 essential nodes discussed in the method section (grey nodes in Fig. 3).

**Fig. 5:**
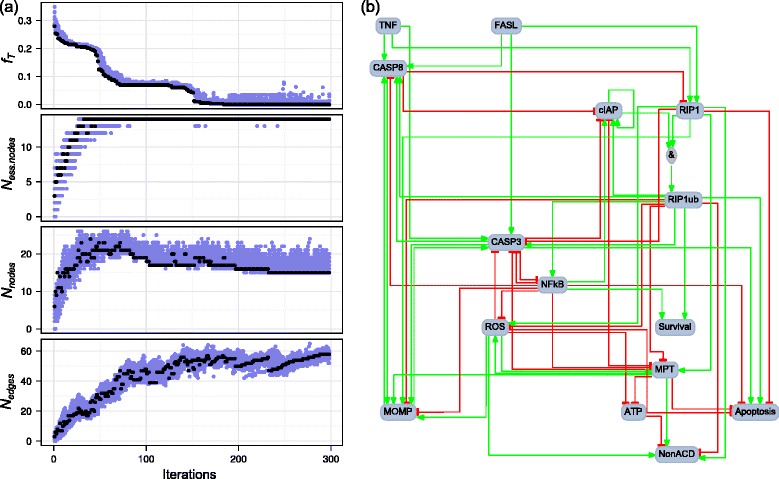
Example of network optimization: best run. **a** Evolution of the fitness function **F** = (*f*_*T*_, − *N*_*ess. nodes*_, *N*_*nodes*_, − *N*_*edges*_) during one run of the optimization method using the training set 1 given in Additional file 2, the PKN given in Additional file 4 (PKN 1) and the 14 essential nodes from the reduced cell-fate decision model proposed by Calzone and co-authors [11]. For each given iteration, *blue dots* correspond to individual replicas and *black dot* to the replica with best value of the fitness function. **b** Network with best value of the fitness function obtained during this run. The run shown in this figure is the one that produced the network with minimal value of the fitness function among 500 independent runs

Figure 5a illustrates how the individual components of the fitness function **F** = (*f*_*T*_, − *N*_*ess. nodes*_, *N*_*nodes*_, − *N*_*edges*_) evolve during the run giving the network with the minimal fitness function value. Following an initialization of all replicas to the empty network, the optimization process starts with an initial phase where *f*_*T*_ decreases while *N*_*ess. nodes*_, *N*_*nodes*_ and *N*_*edges*_ all increase as the networks are populated with nodes and edges. The number of nodes and edges is large enough to include all essential nodes (*N*_*ess. nodes*_ = 14) for most networks after the 50th iteration, and when *N*_*ess. nodes*_ saturates the number of nodes starts to decrease. The number of edges tends to increase steadily from the first to the last iteration, but increases faster whenever the number of nodes increases or saturates. This is a consequence of the lexicographical ordering of multi-dimensional fitness functions. The optimization is stopped when the best value of the fitness function fails to decrease during 10 consecutive iterations, and the network with best value of the fitness function is retained as the output of this optimization run.

Figure 5b presents the best network obtained during this optimization run. It has a first fitness function component *f*_*T*_ = 0, which means that it perfectly reproduces all the experiments in the training set. The second component (*N*_*ess. nodes*_ = 14) is also optimal. The third component *N*_*nodes*_ = 15 is very close to the minimal value 14. Although it may not be optimal, the fact that no network was found with *N*_*nodes*_ = 14 and *f*_*T*_ = 0 in any of the 500 runs suggest that *N*_*nodes*_ = 15 is the minimum number of nodes compatible with *f*_*T*_ = 0. It is not clear whether the fourth component (*N*_*edges*_ = 58) is optimal since it is much smaller than the 99 edges from the PKN connecting the 15 nodes of this network. Therefore, while this network may not be an exact global minimum of the fitness function, it seems to be very close, which suggests that our implementation of the genetic algorithm is appropriate to handle this minimization problem.

The goal of our network optimization method is not only to find networks that can reproduce all experiments in the training set (*f*_*T*_ = 0), but also, and most importantly to find networks that can predict the outcome of experiments against which they were not trained. Since the gold standard network used to generate the PKN and the training set is known, this can be quantified by evaluating the *s*_*all*_ score (defined in the Methods section) which measures the similarity between the average attractors reached by the model network and the average attractors reached by the gold standard network after all possible single node perturbations. Note that contrary to *f*_*T*_, which only takes into account a subset of the attractors that best match the training set, the *s*_*all*_ score takes into account all attractors reached after perturbation, thus penalizing the situation where only a subset of attractors behave properly. For the model network given in Fig. 5b, *s*_*all*_ ≃ 0.974, so approximately 97.4 % of the predicted node states are correct following single node perturbations. Although only one attractor of the unperturbed gold standard network was part of the training set, the model network nevertheless recovered the same 4 attractors as the gold standard network, with only one error (node CASP8 = 1 instead of 0 in one attractor).

**Fig. 6:**
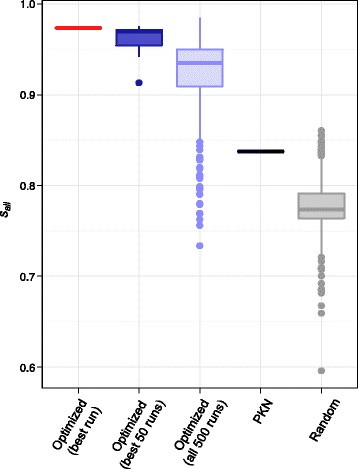
Example of network optimization: *s*_*all*_ scores distribution. Distribution of *s*_*all*_ scores obtained with 500 independent runs of the optimization method using the same input data as in Fig. 5. For each run, only the network with minimal value of the fitness function was kept. This figure shows the *s*_*all*_ scores for the best network (*red line*), for the 50 best networks (*dark blue boxplot*) and for all 500 networks (*light blue boxplot*). In addition, *s*_*all*_ scores for the PKN (*black line*) and 350 random sub-networks of the PKN (*grey boxplot*) are shown

We next examined the diversity of model networks that can be obtained by running the optimization method multiple times. For each of the 500 independent runs we selected the single best network, and from these 500 optimized networks then selected the 50 networks with the lowest fitness function, which were retained as model networks. While none of these networks necessarily represents an exact global minimum of the fitness function, all networks had optimal values for the first and second components (*f*_*T*_ = 0 and *N*_*ess. nodes*_ = 14). The best 50 model networks were compared to the gold standard model by measuring the *s*_*all*_ scores and the resulting distribution of *s*_*all*_ scores is shown in Fig. 6 (dark blue) along with the score for the best network (red). The median *s*_*all*_ of the 50 best networks is high, with about 97 % of node states correctly predicted after all single node perturbations, and significantly higher than the distribution of *s*_*all*_ scores for all the 500 networks (light blue). This shows that at least for this sample input data set, networks with small values of the fitness function tend to have high *s*_*all*_ scores, i.e. their predictions are close to the gold standard network predictions.

To check that the good scores obtained by the model networks were not only due to a very informative PKN, which by construction contains all the functional interactions of the gold standard network, we also measured the *s*_*all*_ score of the PKN (black line in Fig. 6). As shown in Fig. 6, scores of the best 50 model networks (dark blue) are significantly higher than the score of the PKN (black), indicating that the optimization method indeed generates models with much improved predictive power compared to the PKN used as input. The *s*_*all*_ scores for random sub-networks of the PKN (shown in grey) are also significantly lower than those for the 50 model networks. This result is not entirely trivial, since these random networks are built with edges of the PKN that correspond to functional interactions of the gold standard network.

### Genetic algorithm

The quality of the optimization method, as measured by the *s*_*all*_ score, is determined by the combination of the optimization algorithm (genetic algorithm) and the choice of fitness function. In the previous section, we showed that the optimization method was able to generate model networks with good predictive power (high *s*_*all*_ scores). This result strongly suggests that the choice of fitness function was adequate and that the optimization algorithm was able to find sufficiently good solutions to the minimization problem. However, it does not say anything about the efficiency of the optimization algorithm, and indeed a simple random network generator could also produce optimal solutions in principle, simply by generating a sufficiently large number of networks.

**Fig. 7:**
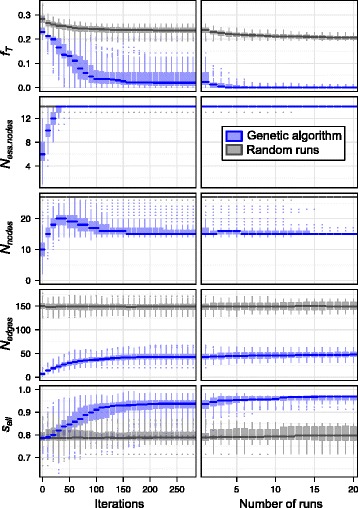
Genetic algorithm evaluation. *Left panel*: evolution of fitness function **F** = (*f*_*T*_, − *N*_*ess. nodes*_, *N*_*nodes*_, − *N*_*edges*_) and *s*_*all*_ score for the best network (i.e. with minimal fitness function) obtained during a run of the optimization method using the same input data as in Fig. 5. *Blue boxplots* summarize values obtained with 200 independent runs of the optimization method. *Grey boxplots* show corresponding results obtained with 200 “random runs”. Note that each *box* summarizes values obtained during 10 iterations (2000 data points). *Right panel*: fitness function and *s*_*all*_ score for the best network (according to the fitness function) obtained after a specified number of runs (horizontal axis). Each *box* summarizes 500 values, obtained by randomly sampling the given number of runs among 200 independent runs of the optimization method

To evaluate our implementation of the genetic algorithm, we compared the evolution of best fitness function values obtained by our algorithm to best fitness function values obtained on a population of random networks. We started 200 independent runs of the optimization method, using the same input as in the previous section (training set 1 in Additional file 2, PKN 1 in Additional file 4 and 14 grey nodes in Fig. 3 as essential nodes). During each run, the fitness function value and *s*_*all*_ score of the best network (i.e. with minimal fitness function value) obtained were stored for each iteration of the genetic algorithm (black dots in Fig. 5a). The resulting distributions of best fitness function values and corresponding *s*_*all*_ scores obtained with the 200 runs are shown in Fig. 7 (blue boxplots, left panel) as a function of the number of iterations of the genetic algorithm. In parallel, for each run of the optimization method, a corresponding “random run” was created by generating, for each iteration, exactly the same number of random networks (random sub-networks of the PKN) as the number of networks generated by the genetic algorithm. For each iteration of the random run, we stored the fitness function value and *s*_*all*_ score of the best random network (i.e. with minimal fitness function value) obtained since the beginning of the run. The resulting distributions of best fitness function values and corresponding *s*_*all*_ scores obtained with the 200 “random runs” are shown in Fig. 7 (grey boxplots, left panel) as a function of the number of iterations. Note that although Fig. 7 presents data for iterations up to 285, the average run length was 171 iterations. Missing values from the end of each run until iteration 285 were replaced by the fitness function value and *s*_*all*_ score obtained in the last iteration of the run.

When comparing results obtained with the genetic algorithm (blue) and random runs (grey), it is clear that the genetic algorithm is much more efficient at generating networks with low values of the fitness function. This is particularly apparent for the first component of the fitness function (*f*_*T*_ in upper left panel of Fig. 7), where optimized networks reach a median value *f*_*T*_ ≃ 0.020, while random runs reach only a median *f*_*T*_ ≃ 0.236. In addition, while the other components of the fitness function (*N*_*ess. nodes*_, *N*_*nodes*_ and *N*_*edges*_) tend to approach their optimal values in a similar way as in Fig. 5a using the genetic algorithm, this is not the case for the random runs. More importantly, the *s*_*all*_ score (lower left panel) increases quickly for optimized networks (blue) to reach a median value *s*_*all*_ ≃ 0.938, while it only marginally increases for random runs to reach a median *s*_*all*_ ≃ 0.788.

In addition to the distribution of best fitness function values obtained during one run discussed above, we also characterized the distribution of best fitness function values obtained after multiple runs (right panel of Fig. 7). This is particularly relevant in the context of optimization based on genetic algorithms, where it is common practice to start multiple independent runs in parallel. We randomly sampled the specified number of runs (horizontal axis) among the 200 runs, storing the fitness function value and *s*_*all*_ score of the best network (according to the fitness function) found in the sampled runs. This process was repeated 500 times for each number of runs, so that each boxplot summarizes 500 values. Using multiple runs has a dramatic impact on the best value of the fitness function obtained with the genetic algorithm (blue boxes): the median value of the first component (*f*_*T*_ in upper right panel) decreases from *f*_*T*_ ≃ 0.020 with one run to *f*_*T*_ = 0 with 20 runs, while its dispersion strongly reduces, with an interquartile range that decreases from 0.052 with one run to 0 with 20 runs. Similarly, while the median *s*_*all*_ score increases from *s*_*all*_ ≃ 0.938 with one run to *s*_*all*_ ≃ 0.970 with 20 runs (lower right panel), its dispersion is reduced, with an interquartile range that decreases from 0.034 to 0.017. The other components of the fitness function (*N*_*ess. nodes*_, *N*_*nodes*_ and *N*_*edges*_) are only marginally improved by increasing the number of runs.

The best value of the fitness function obtained with random runs (upper panel, grey boxes) also improves when increasing the number of runs, but it is still far from the best values obtained with the genetic algorithm. The *s*_*all*_ score obtained with random runs (lower panel, grey boxes) only slightly increases from *s*_*all*_ ≃ 0.788 with one run to *s*_*all*_ ≃ 0.797 with 20 runs.

To summarize, Fig. 7 shows that our implementation of the genetic algorithm is significantly more efficient than random sampling in finding solutions to the fitness function minimization problem.

### PKN quality

In the previous sections we used an ideal PKN which contained only (direct or indirect) interactions observed after *in silico* perturbation experiments of the gold standard network. However, a PKN built from the literature will usually not be perfect; it may contain “noise” in the form of interactions that are not relevant to the particular biological context under study, while some relevant interactions may also be missing.

**Fig. 8:**
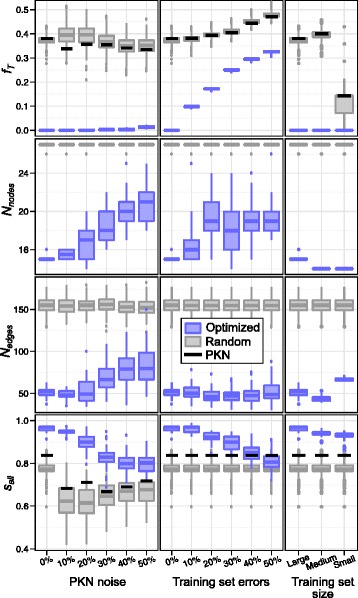
PKN quality and training set size. Fitness function **F** = (*f*_*T*_, − *N*_*ess. nodes*_, *N*_*nodes*_, − *N*_*edges*_) and *s*_*all*_ score for model networks (*blue boxplots*), random sub-networks of the PKN (*grey boxplots*) and PKN (*black line*) as a function of noise in the input PKN (*left panel*), errors in the training set (*centre panel*) and size of the training set (*right panel*). *Left panel*: results based on the training set 1 given in Additional file 4 and PKNs 1 to 6 (Additional file 3). *Centre panel*: results based on training set 1 with 0 to 50 % error (Additional file 2) and PKN 1 (Additional file 4). *Right panel*: results based on training sets 1, 2 and 3 (Additional file 2) and PKN 1 (Additional file 4). Each *blue boxplot* summarizes measurements on 50 model networks obtained out of 500 independent runs of the optimization method (more details in main text). Each *grey boxplot* summarizes measurements on 350 random sub-networks of the PKN used as input for the optimization method. Component *N*_*ess. nodes*_ of the fitness function was always optimal (*N*_*ess. nodes*_ = 14) and is not shown in this figure

To investigate the effect of noise in the PKN on the optimization method, we generated five PKNs (PKN 2 to 6 in Additional file 4) by adding 10, 20, 30, 40, and 50 % of noise to the ideal PKN, as defined in Methods section. For each PKN, we started 500 independent runs of the optimization method and kept the best network (with minimal value of the fitness function) obtained with each run. Among the 500 resulting networks, only the 50 with minimal values of the fitness function were kept as model networks. The left panel of Fig. 8 presents the distribution of fitness function values and *s*_*all*_ scores as a function of the PKN used as input (horizontal axis) for the resulting model networks (blue boxes), for random sub-networks of the PKNs used as input (grey boxes) and for the PKNs (black lines). The values of the fitness function obtained with model networks increase (worsen) as the fraction of noise in the PKN increases, with the median *f*_*T*_ reaching *f*_*T*_ ≃ 0.015 with 50 % noise. The number of nodes and edges also increases with noise, suggesting that it is not possible to find simple networks that are able to reproduce training set experiments when too many interactions are missing in the PKN.

As expected, the predictive power of model networks (*s*_*all*_ score, lower left panel) decreases with increasing noise in the PKN. However, the median *s*_*all*_ score is always above 0.798 even with 50 % noise in the PKN. In addition, the distribution of *s*_*all*_ scores is always significantly higher for model networks (blue) than for random networks (grey) and PKN (black). The decrease of *s*_*all*_ scores with increasing PKN noise suggests that our method makes good use of the information contained in the PKN, and that a carefully constructed PKN can increase the quality of the resulting model networks. The fact that model networks always have better *s*_*all*_ scores than PKNs also shows that our method is rather tolerant to errors in the PKN. Indeed, even with 50 % noise in the PKN, our method is able to output model networks that have more predictive power than the input PKN. However, if the PKN is known to be very noisy, it could be worth considering other methods which are not based on prior knowledge.

### Training set quality

In the previous section we used an ideal training set obtained by measuring the response of the gold standard network to perturbations. To evaluate the robustness of the optimization method to errors in the training set, we generated five training sets by adding 10, 20, 30, 40, and 50 % of errors to the ideal training set (training sets 1 with 10 to 50 % error in Additional file 2), as described in the Methods section.

For each training set, we generated 50 model networks from 500 independent runs by following the procedure described in the previous sections. The centre panel of Fig. 8 presents the distribution of fitness function values and *s*_*all*_ scores as a function of the fraction of errors in the input training set for the resulting model networks (blue boxes), for random sub-networks of the PKN (grey boxes) and for the PKN (black lines).

The values of the fitness function dramatically increase (worsen) when the number of errors in the training set is increased. In particular, while model networks obtained with the ideal training set always perfectly reproduce all experiments in the training set (*f*_*T*_ = 0), about 9.9 % of the training set cannot be reproduced by model networks when the training set contains 10 % of errors (median *f*_*T*_ ≃ 0.099). When the training set contains 50 % of errors, *f*_*T*_ reaches a median value of 0.327, which means that model networks fail to reproduce about 33 % of the training set. This suggests that it is not possible to find a model network, built as a sub-graph of the PKN, which is able to reproduce all errors in our training set.

As expected, the predictive power of model networks (*s*_*all*_ score, lower centre panel) decreases when increasing the fraction of errors in the training set, but this decrease is moderate. For instance, *s*_*all*_ scores are not significantly lower with 10 % of errors in the training set (median *s*_*all*_ ≃ 0.969) than with the ideal training set (median *s*_*all*_ ≃ 0.970). Moreover, for all training sets with up to 30 % of errors, *s*_*all*_ is clearly higher for model networks (blue) than for random sub-networks of the PKN (grey) and for the PKN (black). Only when the training set contains 50 % of errors does the median *s*_*all*_ score of model networks become lower than the *s*_*all*_ score of the PKN. However, even in this case, the median predictive power of model networks is still above 80 %. The moderate decrease of predictive power, together with the rapid increase of fitness function values when the number of errors in the training set increases, suggest that our optimization method is not greatly affected by overfitting. The use of a PKN contributes greatly to this result by drastically reducing the number of parameters in the model.

### Training set size

In the previous experiments we used a comprehensive training set that included the states of all 14 essential nodes measured before and after over-expression of TNF, FASL or the combination of TNF and FASL, for seven mutant versions of the gold standard network (training set 1 in Additional file 2). To study the effect of reducing the training set coverage on the optimization method, we generated two smaller training sets (see Methods section): a “medium” training set, which contains data for the wild-type mutant only (training set 2 in Additional file 2) and a “small” training set, containing only the initial physiological state (training set 3 in Additional file 2). Clearly, the small training set does not contain enough information to properly infer a model network, and it was used to study the behaviour of the optimization method in this limit. Following the same procedure as in the previous sections, for each training set we used the optimization method to generate 50 model networks out of 500 independent runs. The resulting distributions of fitness function values and *s*_*all*_ scores are shown in the right panel of Fig. 8 (blue boxes) as a function of the training set used as input (horizontal axis), together with fitness function values and *s*_*all*_ scores measured on random sub-networks of the PKN (grey boxes) and directly on the PKN (black lines). Although model networks always perfectly reproduce all experiments in the training sets (*f*_*T*_ = 0, upper right panel), other components of the fitness function tend to improve when the size of the training set decreases. In particular, the number of nodes reaches its optimal value (*N*_*nodes*_ = 14) for medium and small training sets, while networks obtained with the small training set have more edges than networks obtained with large training set. This is due to the smaller training sets imposing fewer constraints on the networks than larger training sets, which leave more freedom to optimize the remaining components of the fitness function.

Although the predictive power of model networks decreases with training set size (*s*_*all*_ score, lower right panel), it remains significantly higher than that of random sub-networks of the PKN (grey) and the complete PKN (black) for all training sets. Interestingly, while the small training set contains only the states of 14 essential nodes in one attractor of the unperturbed gold standard network, the resulting model networks still have a median *s*_*all*_ of 0.937, which means that approximately 94 % of the 1624 nodes states measured after all single nodes perturbations are predicted correctly.

To summarize, although the predictive power of model networks decreases with the quantity of information contained in the training set, our optimization method was still able to produce networks with significantly improved predictive power compared to the input PKN and random sub-networks of the PKN when using reduced training sets.

### Fitness function: maximizing versus minimizing number of edges

The choice of fitness function is an essential ingredient of network optimization methods. While the first two components of our fitness function (*f*_*T*_ and *N*_*ess. nodes*_) can be easily understood, our choice of third and fourth components (*N*_*nodes*_ and *N*_*edges*_) may not be so obvious. Indeed, these last two components were chosen to first minimize the number of nodes and then maximize the number of edges (among networks with same number of nodes), whereas a more usual choice of regularization consists in minimizing the number of edges.

**Fig. 9:**
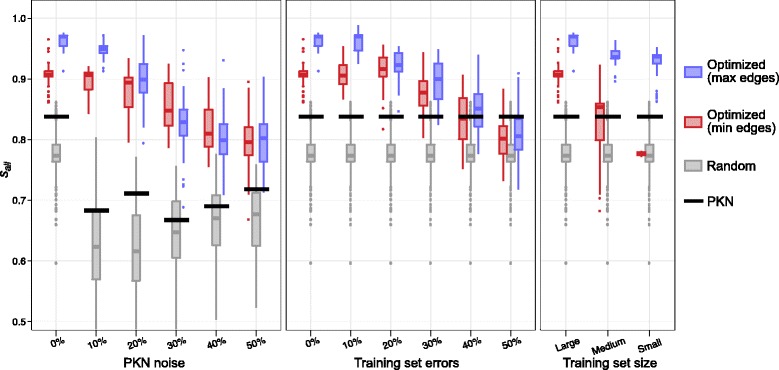
Fitness function: maximizing versus minimizing number of edges. Comparison of predictive power (*s*_*all*_ score) of model networks obtained with the fitness function **F** = (*f*_*T*_, − *N*_*ess. nodes*_, *N*_*nodes*_, − *N*_*edges*_) which minimize number of nodes and subsequently maximize number of edges (*blue boxplots*), and fitness function **F** = (*f*_*T*_, − *N*_*ess. nodes*_, *N*_*edges*_) which minimize number of edges (*red boxplots*). Except for *s*_*all*_ scores obtained by minimizing the number of edges (*red*), all results were taken from Fig. 8. Model networks optimized with minimization of the number of edges (*red*) used the same input data sets and the optimization procedure as in Fig. 8, except for the change of fitness function, and the use of 100 instead of 50 replicas

To motivate our choice of fitness function, we used our optimization method with the modified fitness function **F** = (*f*_*T*_, − *N*_*ess. nodes*_, *N*_*edges*_) which minimizes the number of edges and does not constrain the number of nodes. For each input data set discussed in the previous sections, 50 model networks were generated out of 500 independent runs. Due to slower convergence of the genetic algorithm with this fitness function, we had to use 100 replicas instead of 50 to obtain model networks with comparable fitness function values. The resulting distributions of *s*_*all*_ scores are shown in Fig. 9 (red boxes), together with *s*_*all*_ scores obtained previously by minimizing number of nodes and maximizing number of edges (blue boxes). In addition, *s*_*all*_ scores measured on random sub-networks of the PKN (grey boxes) and directly on the PKN (black lines) are also shown for comparison. In the following, we will abbreviate the approach of “maximizing the number of edges while minimizing the number of nodes” as simply “maximizing the number of edges”. The additional constraint on the node number has to be kept in mind.

Clearly, the predictive power of model networks obtained by minimizing the number of edges is less sensitive to noise in the PKN used as input (left panel). With no or low noise, maximizing the number of edges is clearly a good strategy, as the *s*_*all*_ scores are significantly lower when minimizing the number of edges. When PKN noise increases, both approaches give comparable results, with slightly better, although not significantly, *s*_*all*_ scores obtained by minimizing number of edges with 30 and 40 % noise.

Similarly, when the training set contain errors (centre panel), model networks obtained by maximizing the number of edges tend to have higher predictive power. Although the difference is more pronounced for low fractions of errors in training sets, median *s*_*all*_ scores obtained by maximizing the number of edges are always higher than scores obtained by minimizing the number of edges.

The advantage of maximizing the number of edges becomes more striking when decreasing the size of the training set used as input (right panel). Indeed, while networks obtained by maximizing the number of edges have a median *s*_*all*_ score that slowly decreases from 0.970 to 0.937, networks obtained by minimizing the number of edges always have significantly lower *s*_*all*_ scores, reaching a median of 0.778 with small training set. More importantly, although networks obtained by minimizing the number of edges are significantly better than random sub-networks of the PKN and the PKN itself when using a large training set, they are not much better than the PKN when using a medium training set, and significantly worse than the PKN when using a small training set, with a median *s*_*all*_ score 0.778 barely better than the median *s*_*all*_ score 0.774 of random sub-networks of the PKN.

These results can be understood by realizing that maximizing the number of edges generates networks which are as close as possible to the PKN, only removing edges that are in contradiction with the training set, and therefore use a maximum of information contained in the PKN. This is particularly interesting in the limit of small training sets, when the information contained in the training set is not sufficient to properly infer a model network. In this limit, in addition to the training set, our method heavily uses the information contained in the PKN, and therefore generates model networks with reasonably good predictive power. By contrast, minimizing the number of edges within the limit of small training sets results in over-simplified model networks that are not able to properly predict anything else than what they were trained for.

This reasoning also explains why maximizing the number of edges is a better strategy when the quality of PKN is good, and becomes less attractive in the limit of very noisy PKN. What is interesting, and somewhat unexpected, is the fact that maximizing edges does not give significantly worse results than minimizing edges in the limit of very noisy PKNs.

The main motivation behind minimizing the number of edges is usually based on Occam’s razor, i.e. the principle of parsimony. However, this approach can lead to oversimplification of the resulting model, for instance by removing alternative pathways that are necessary to ensure the known robustness of regulatory networks. The idea behind maximizing the number of edges is to keep this robustness and to reflect the complexity of biological networks. For a node with various documented biological functions, it seems artificial to reduce that node to only one of them, assuming that the input PKN was built carefully.

### Combined predictions

In general, one should not expect the optimization problem discussed here to have a unique solution (network). If the training set does not contain enough information to completely constrain the problem then multiple solutions may be possible. If essential interactions are missing from the PKN then no sub-network of the PKN (model network) may be able to exactly reproduce all experiments in the training set (*f*_*T*_ = 0) and a potentially large number of solutions with equally good *f*_*T*_ > 0 may be possible. Even when the optimization problem does have a unique optimal solution, heuristic optimization methods, like the genetic algorithm that we use, might be unable to find it, and instead output multiple sub-optimal networks with similar fitness function values. For all these reasons, a large set of model networks may be equally good solutions to the optimization problem.

This multiplicity of model networks is not in contradiction with a unique network describing the underlying biological system (in this work: the gold standard network), but only reflects our lack of knowledge on the system. To summarize all these solutions, a common practice is to generate an average network as the union of all models networks, with a weight attached to each edge, such as the number of model networks in which it appears. However, unless all model networks are very similar, this consensus network is of limited interest since it does not retain the topology (feed-back loops, connectivity), and more importantly, the dynamical behaviour (attractor reachability graph) of the original model networks. Instead, we propose to summarize our results at the level of the predictions by measuring averages but also variability of node states predicted by all model networks. Intuitively, if a node state prediction varies a lot across networks, this could be due to a lack of information in the PKN and training set, and therefore could be correlated to prediction errors.

To check whether prediction variability was correlated to prediction errors, we summarized the predictions of the 50 model networks obtained previously with each input data set (PKN and training set). We used the same predictions that were used to measure the *s*_*all*_ score (see Methods section), i.e. the average states reached by a network after each single node perturbation of essential nodes, starting from each attractor of the gold standard network. For each input data set (PKN and training set), single node perturbation and initial state (gold standard network attractor), we evaluated the average and variance of the 50 average states predicted by the 50 model networks. We also evaluated the corresponding prediction error by measuring the absolute difference between average model networks’ predictions and average states reached by the gold standard network. This procedure led to 21,112 error versus variance measurements, one for each of the 14 essential nodes, with 29 single node perturbations (unperturbed, 14 nodes forced to 1, 14 nodes forced to 0), 4 attractors of the gold standard network and 13 input data sets (more details on this procedure are given in Additional file 1).

**Fig. 10:**
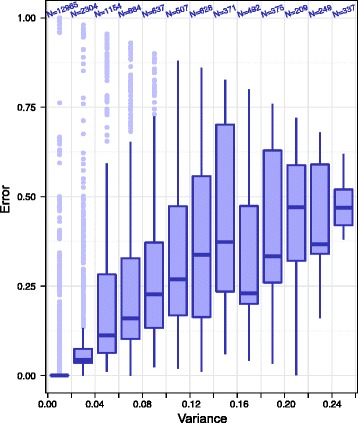
Combined predictions: error versus variance. Distribution of errors as a function variance obtained by summarizing the predictions of the 50 model networks obtained which each input data set (more details in main text). The number *N* above each *boxplot* denotes the number of measurements in the boxplot

The resulting distribution of error versus variance is shown in Fig. 10. This figure shows a strong correlation between error and variance, confirmed by a Spearman’s rank correlation coefficient of ≃ 0.96. This result suggests that the prediction error, which is usually unknown, can be (approximately) estimated by measuring the prediction variance provided that not only one, but multiple model networks are kept as solutions of the optimization problem.

Ideally, variance should be estimated based on the full population of networks which are solutions of our optimization problem, or at least on a uniformly sampled subset of the solutions. However, due to the heuristic approach used here, our optimization method should in principle be unable to generate an exhaustive list of solutions, or to uniformly sample the set of solutions. The strong correlation observed in Fig. 10 is therefore an interesting and non-trivial result, which shows that despite these limitations, our optimization method is able to sample sufficiently well the space of solutions.

### Related work

A large amount of methods focusing on the problem of Boolean network inference have been published. In the following we will consider only methods that are similar to our method in the sense that they combine the use of experimental data against which Boolean networks are trained, together with prior knowledge to reduce the size of the search space. These methods are classified according to the type of data used in the training set.

Several methods require training sets in the form of time series of experimental measurements. REACT [13] and CellNopt [14] use evolutionary algorithms to train networks against time series obtained with various network perturbations. Both methods only consider networks with synchronous dynamics. By assuming that the consecutive measurements in the time series corresponds to successive states of the network with synchronous dynamics, Breindl et al. [4] can use linear programming to solve the network optimization problem. BoolNet [15] implements the Best-Fit Extension [16] and REVEAL [17] algorithms to reconstruct networks from time series obtained with various network perturbations, and can use synchronous as well as asynchronous dynamics. RE:IN [18] uses a Satisfiability Modulo Theories (SMT) solver to exhaustively find all networks that can exactly reproduce the training set, which consists of time series obtained under various network perturbations. Only synchronous dynamics is implemented in this method.

Other methods focus on early responses to perturbations and only consider a small subset of two time points in each time series. Only the initial measurement and a second carefully chosen time point reflecting the initial response to the perturbation are kept in the training set. In addition to time series discussed above, CellNopt [14] also implements a method based on a genetic algorithm to train networks on the early response to perturbations, assuming synchronous dynamics of the networks. Guziolowski et al. [19] and Videla et al. [5] use answer set programming to enumerate all networks that exactly reproduce the initial response to perturbations using synchronous dynamics, but also all suboptimal networks within a user defined tolerance.

Another popular approach is to use a training set containing stable phenotypes assumed to be steady states (attractors with one state) of the network. These training sets contain only equilibrium properties of the network and lack information on the dynamics of the network. XPRED [9] and PRUNET [10] use evolutionary algorithms to find networks that have steady states as close as possible to the phenotypes given in the training set. Knapp and Kaderali [3] reformulated the problem of finding networks with specific steady states obtained under various perturbations as a linear programming problem. A runtime comparison with these methods is given in Additional file 5.

Our optimization method contrasts with the aforementioned approaches in a number of ways. First, our method combines information on both the dynamics (time series) and equilibrium properties (steady states) of the networks, while the aforementioned approaches use only one of these types of information. Indeed our training set can contain measurements performed on stable phenotypes that are assumed to correspond to attractors of the networks, together with measurements on their reachability upon perturbation of the network. Second, our method uses asynchronous dynamics, which is usually considered as more relevant for the description of biological networks than synchronous dynamics [20]. By contrast, all methods discussed above, except BoolNet, use synchronous dynamics. Finally, our method attempts to maximize the number of edges in order to be as close as possible to the input PKN, while only Videla et al. [9] suggest that minimizing the number of edges may not be the optimal approach.

## Conclusions

Within the last years the wealth of experimental data from high-throughput technologies in different areas of biology has popularized the construction of a PKN to summarize and visualize the knowledge derived from this data. Unfortunately, although it is technically possible to directly transform such a network as a dynamical Boolean model, it may not behave as expected for a specific biological process because it usually includes interactions described in different biological contexts and/or experimental conditions, some of which could be absent in the biological process under study. The presence of these “wrong” or “inactive” interactions in the model may dramatically change its dynamical behaviour with consequent lack of reliability of its predictions.

Here we propose a method to generate and optimize dynamical Boolean models by training a given PKN against experimental data describing either stable states or response to perturbation or both. The output of our method is a set of sub-networks of the PKN contextualized to the experimental conditions used to train the model. Simulations performed on such a network should yield more reliable predictions, helping researchers in hypothesis generation and experimental design. The general applicability of the method in a variety of biological contexts will make this approach of interest to biological and medical researchers.

Future developments will include the implementation of the same strategy on a multi-valued discrete system (i.e. non-Boolean), which should allow a more precise description of gene activities and network dynamics. The current method could also be adapted to deal with the cyclic behaviour and time series of oscillatory processes.

## Additional files

Additional file 1: Methods. Detailed description of the methods. (PDF 1329 kb)
Additional file 2: Cell-fate decision model: *in silico* training sets. Training sets used to evaluate the optimization method. Each training set is given in the form of a transition graph, where each node contains a perturbation with the corresponding observation (stable phenotype) and edges denote a transition between stable phenotype upon change of condition (perturbation). Perturbations are specified using a combination of node names prefixed by – or + sign to specify knock-out (node state fixed to 0) or over-expression (node state fixed to 1). When multiple edges connect the same source node to different target nodes, it means that all transitions start from the same phenotype (same attractor of the network). Node states that were intentionally reversed to generate errors in training sets are shown in red. (PDF 9015 kb)
Additional file 3: Cell-fate decision model: single node perturbations. List of transitions between attractors from unperturbed to single node perturbations. Each line corresponds to one edge in the attractor reachability graph. Attractors with more than one state are replaced by the average over all states in the attractor. (PDF 63 kb)
Additional file 4: Cell-fate decision model: *in silico* PKNs. In silico PKNs for the cell-fate decision model. For each PKN, a graphical representation as well as the list of interactions is given. Interactions that were changed to introduce noise in the PKNs are shown in blue in the list of interactions. In the graphical representation, interactions that were changed to introduce noise in the PKNs are shown in magenta (negative interactions) and cyan (positive interactions). (PDF 314 kb)
Additional file 5: Comparison with related methods. Runtime comparison between our implementation of the method (optimusqual) and related methods. (PDF 1190 kb)
