# Supplementary material for: Boolean regulatory network reconstruction using literature based knowledge with a genetic algorithm optimization method
Source: BMC Bioinformatics. 2016 Oct 6;17:410. doi: 10.1186/s12859-016-1287-z (PMC5053080; doi:10.1186/s12859-016-1287-z)
Supplement: Additional file 1: — Methods. Detailed description of the methods. (PDF 1329 kb) [file 12859_2016_1287_MOESM1_ESM.pdf]

Methods

Julien Dorier*[1] , Isaac Crespo[1] , Anne Niknejad[1] , Robin Liechti[1] , Martin Ebeling[2] and Ioannis Xenarios*[1]

[1]Vital-IT, SIB Swiss Institute of Bioinformatics, 1015 Lausanne, Switzerland

[2]Pharmaceutical Sciences / Translational Technologies and Bionformatics, Roche Innovation Center Basel, 124 Grenzacherstrasse, 4070 Basel, Switzerland

Email: Julien Dorier *- julien.dorier@isb-sib.ch; Isaac Crespo - isaac.crespo@isb-sib.ch; Anne Niknejad - Anne.Niknejad@isb-sib.ch; Robin Liechti - Robin.Liechti@isb-sib.ch; Martin Ebeling - martin.ebeling@roche.com; Ioannis Xenarios *- ioannis.xenarios@isb-sib.ch;

*Corresponding author

Description of the network optimization method

Evaluation of attractor reachability graphs

To evaluate the attractor reachability graph of a network, we use two methods.

- boolSim, a software developed by Garg and coauthors [1], which uses an implicit method based on reduced ordered binary decision diagrams to evaluate the attractors' reachability graph of a network. This method is exact and exhaustively finds all attractors but quickly becomes too computationally expensive for large networks. In this work, it is used only to compare the resulting model networks to the original gold standard network (score s_{all}).
- The second method is a simple algorithm based on a stochastic exploration of the state transition graph. It has the advantage of being faster and scaling better than boolSim with system size, at the cost of completeness since there is no guarantee to find all attractors.

The stochastic method is based on the following algorithm used to find the attractors of a network with perturbation P, starting from a specified set of initial states. In addition to the network, the perturbation and the set of initial states, this algorithm takes as input a number of paths N_{paths}, a maximal number of iterations $N_{\max}$ and a number of iterations N_{stored} used to estimate the approximate state transition graph $\tilde{G}$. We describe the algorithm for asynchronous updates, but it can be trivially generalized to synchronous updates:

1. Randomly choose one network state x_{init} from the set of initial states. Set $x = x_{\text{init}}$ and clear the approximate state transition graph $\tilde{G}$.
2. Perform $(N_{\max} - N_{\text{stored}})$ iterations of a random walk to reach a state closer to an attractor. At each iteration of the random walk, evaluate all successors $\{x'_0, x'_1, \cdots\}$ of state x using asynchronous updates and the constraints given by the perturbation P (nodes states fixed to 0 or 1). If the set of successor states is empty, then x is a steady state: add an edge $x \to x$ to the approximate state transition graph $\tilde{G}$ and stop the exploration of this path and go to step (4). Otherwise, randomly choose the next state $x \in \{x'_0, x'_1, \cdots\}$ (with uniform probability) and continue the random walk.
3. Perform a depth first search of the state transition graph, starting from the last state obtained during the random walk (denoted x_{root}). At each iteration, store the current state x and all transitions to successor states $\{x \to x'_0, x \to x'_1, \cdots\}$ as edges in the approximate state transition graph $\tilde{G}$. If the set of successor states is empty, then x is a steady state: add an edge $x \to x$ to the approximate state transition graph $\tilde{G}$ and stop the exploration of this path and go to step (4). Stop the depth first search

and go to (4) whenever all states downstream of $x_{\rm root}$ were discovered or if the number of iterations reaches $N_{\rm stored}$.

4. The resulting graph $\tilde{G}$ approximates the exact state transition graph in the limit of long term evolution of the network. Attractors of the network are obtained as the set of terminal strongly connected components [2] of $\tilde{G}$ that contain at least one feedback loop. This constraint on the presence of feedback loops is used to distinguish between a strongly connected component with a single node without feedback loops, which corresponds to an unfinished path (not necessarily an attractor), and a single node with feedback loop which corresponds to a steady state. If an attractor was found, keep the information on the initial state $x_{\rm init}$ which is necessary to recover the reachability from initial states to attractors.

5. Store attractors found and the corresponding initial state $x_{\rm init}$. Repeat this procedure $N_{\rm paths}$ times from step (1). Output all attractors found, together with information on reachability from inital states to attractors.

The attractor reachability graph of the network can be easily found by applying this algorithm to all transitions from perturbation P_n to perturbation P_m, using attractors found with perturbation P_n as initial state, and evaluating attractors and reachability from initial states for the network with perturbation P_m. If a perturbation P_n does not have any incoming transition from other perturbations, the initial states are taken as the set of all network states compatible with perturbation P_n and with the constraint that network nodes without regulators are fixed to state 0.

The advantage of this method is that it will output only exact attractors of the network. The disadvantage is that not all attractors will necessarily be found. As a consequence, when f_T (see below and in main text) is evaluated using the approximate attractor reachability graph obtained with this method, it will be larger than or equal to the exact f_T.

In this work, we used $N_{\rm paths} = 100$, $N_{\rm max} = 10 N_{\rm nodes}$ and $N_{\rm stored} = N_{\rm nodes}$ with $N_{\rm nodes}$ the number of nodes in the network.

Fitness function

For each network, we define a multi-dimensional fitness function

$$\boldsymbol{F} = (f_T, -N_{\rm ess.nodes}, N_{\rm nodes}, -N_{\rm edges})$$

Its components are defined as:

1. f_T measures how well a given model network reproduces experiments from the training set. In the following, the training set is assumed to be given in the form of a graph, with each node defined by a perturbation P of the system (any combination of nodes with states fixed to 0 or 1) and a corresponding observation O (a list of node states measured at equilibrium after the perturbation). The set of nodes is denoted as $\{(P_0, O_0), (P_1, O_1), \cdots\}$, where P_n and O_n are the perturbation and observation corresponding to node n. Edges in the training set graph correspond to transitions between stable phenotypes. An edge $(P_n, O_n) \to (P_m, O_m)$ means that under perturbation P_n, the system stabilized into a phenotype characterized by observation O_n and after perturbation by P_m, the system stabilized into a phenotype characterized by observation O_m. Given a network and a training set graph, f_T is evaluated as follows:

   - Using the stochastic search described above, evaluate the attractor reachability graph of the network for all perturbations and transitions given in the training set graph. More precisely, for each node (P_n, O_n) of the training set graph, the attractor reachability graph contains one node per attractor found after perturbation P_n. For clarity, we denote by $\{A_{n,0}, A_{n,1}, \cdots\}$ the set of attractors of the network obtained with perturbation P_n. For each edge $(P_n, O_n) \to (P_m, O_m)$ in the training set graph, the attractor reachability graph contains edges connecting attractors $\{A_{n,0}, A_{n,1}, \cdots\}$ to attractors $\{A_{m,0}, A_{m,1}, \cdots\}$. That is, an edge $A_{n,k} \to A_{m,p}$ will be in the

attractor reachability graph if and only if at least one state in attractor $A_{n,k}$ obtained with perturbation P_n will stabilize into attractor $A_{m,p}$ after perturbation P_m.

- For each attractor $A_{n,k}$ obtained with perturbation P_n, measure its Manhattan distance to the observed phenotype O_n as

$$d_{n,k} = \sum_{i=1}^{N_n} |x_i^{(n,k)} - \tilde{x}_i^{(n)}|$$

  where the sum runs over all N_n nodes appearing in observation O_n, $\tilde{x}_i^{(n)}$ is the state of the node i in observation O_n, $x_i^{(n,k)}$ is the average state of the network node i in attractor $A_{n,k}$ (if attractor $A_{n,k}$ contains more than one state, $x_i^{(n,k)}$ is the average node state over all states in $A_{n,k}$). If a node appears in observation O_n but is not in the network, it is considered as isolated and its state is set to $x_i^{(n,k)} = 0$. An illustration is given in Figure S1.

- Next, we want to consider all subgraphs of the attractor reachability graph which have the same structure as the training set graph. More precisely, we consider all subgraphs g satisfying the following conditions:
  - For each node (P_n, O_n) of the training set graph, the subgraph has exactly one node (chosen among the nodes $\{A_{n,0}, A_{n,1}, \cdots\}$)
  - For each edge $(P_n, O_n) \to (P_m, O_m)$ in the training set graph, the subgraph has exactly one edge (chosen among the edges of the attractor reachability graph connecting nodes $\{A_{n,0}, A_{n,1}, \cdots\}$ to nodes $\{A_{m,0}, A_{m,1}, \cdots\}$.

  Note that this is not an isomorphism, since one node or edge from the attractor reachability graph may correspond to more than one node or edge in the training set graph. For each subgraph g satisfying these conditions, evaluate the total distance to the training set as the sum of distances $d_{n,k}$ over all nodes (attractors) in the subgraph:

$$d_g = \sum_{(n,k)\in\mathcal{V}} d_{n,k}$$

  where $\mathcal{V}$ denotes the set of nodes in g. An illustration is given in Figure S2. Among all possible subgraphs, find the one with minimal total distance to the training set:

$$d = \min_g d_g$$

  For the following, we denote by g_{best} the subgraph that minimizes d_g.

- Finally, f_T is obtained by normalizing this minimal total distance d by the total number of observations in the training set

$$f_T = \frac{d}{N_{obs}}$$

  with

$$N_{obs} = \sum_n N_n$$

With this normalization, f_T is between 0 (best) and 1 (worst) and can be interpreted as the fraction of observations in the training set that are not reproduced correctly by the network.

Notes:

- Our choice of using only subgraphs of the attractor reachability graph with the same structure as the training set graph has some important consequences. Firstly, if observation O_n obtained with perturbation P_n is linked to observation O_m obtained with perturbation P_m in the training set graph (edge $(P_n, O_n) \to (P_m, O_m)$), the attractor associated to O_n with perturbation P_n must stabilize into the attractor associated to O_m after perturbation P_m. This reachability condition is strictly enforced, i.e. two attractors which are not connected in the attractor reachability graph

will not be considered for the evaluation of f_T, even if they are closer to their corresponding observations O_n and O_m (smaller distances $d_{n,k}$ and $d_{m,p}$). Secondly, one node (P_n, O_n) in the training set graph must correspond to exactly one attractor of the network after perturbation P_n, that is if a node (P_n, O_n) in the training set graph has two outgoing edges $(P_n, O_n) \to (P_{m_1}, O_{m_1})$ and $(P_n, O_n) \to (P_{m_2}, O_{m_2})$, then the same attractor of the network obtained with perturbation P_n and associated to O_n must stabilize into the attractor associated to O_{m_1} after perturbation P_{m_1} and into the attractor associated to O_{m_2} after perturbation P_{m_2}.

  - Since the attractor reachability graph of the network is obtained by a stochastic search, it may result in an incomplete graph. That is, for a node (P_n, O_n) of the training set graph, the stochastic search may not be able to find any attractor. Similarly, for one edge $(P_n, O_n) \to (P_m, O_m)$ in the training set graph, the stochastic search may not be able to find any transition from attractors obtained with perturbation P_n to attractors obtained with P_m. If the attractor reachability graph is incomplete, it may not be possible to find any subgraph with same structure as the training set graph. In this case, f_T is flagged as invalid.

2. $N_{\text{ess.nodes}}$ is the number of essential nodes which are in the network. The set of essential nodes is a predefined set of nodes that should be included in the final model network.

3. N_{node} is the number of nodes in the network.

4. N_{edges} is the number of edges in the network.

Multi-dimensional fitness functions are compared using lexicographical ordering:

$$\boldsymbol{F} = (f_1, f_2, .., f_n) < \boldsymbol{F}' = (f'_1, f'_2, .., f'_n)$$

$$\Leftrightarrow \exists i \in \{1, \cdots, n\} \text{ s.t. } f_j = f'_j \ \forall j < i \text{ and } f_i < f'_i.$$

Genetic algorithm

The genetic algorithm is implemented as summarized here, with details provided in the following paragraphs:

1. Start with a population of N_r empty model networks, also called replicas in the following (unless specified otherwise, we used $N_r = 50$).

2. Create a new generation of replicas, starting from an empty population:

   - Add the n_1 best replicas from the last generation (we used $n_1 = 0.1 N_r$).
   - Randomly choose n_2 replicas with probability linearly decreasing from the best to the worst replica. Mutate each of these replicas and add them to the new generation (we used $n_2 = N_r$).
   - Randomly choose n_3 pairs of replicas, with each replica chosen with probability linearly decreasing from best to worst replica. For each pair, generate two new replicas by cross-over and add them to the new generation (we used $n_3 = 0.3 N_r$).

   Note that this new generation has $n_1 + n_2 + 2n_3$ replicas.

3. Evaluate the fitness function for each replica. If a replica has an *invalid* f_T (due to a problem with the stochastic method used to estimate the attractor reachability graph), it is removed from the population. Sort the replicas by increasing fitness function, and keep only the first N_r replicas.

4. If the best value of the fitness function did not improve during the last 10 iterations, output the best replica and stop. Otherwise, go to step 2.

Note that the PKN can contain interactions combining multiple input nodes in a Boolean expression with AND and NOT Boolean operators (e.g. gene1 AND NOT gene2 $\to$ gene3). In the following, each of these Boolean expressions is considered as one entity (hyperedge) like any other edge and they are added to or removed from the network as a whole.

Mutation:
Replicas can undergo three types of mutation:

- Addition/removal of edges (priority $p_0 = 10$): randomly remove up to 5 edges from the replica and add up to 5 edges taken from one of the these lists:
  - Edges in PKN but not in replica (probability 0.8).
  - Edges in PKN but not in replica and connecting at least an essential node not yet connected to the network (probability 0.2). This mutation should help to increase the number of essential nodes.
- Addition of paths (priority $p_1 = 5$): Create a list of possible paths in the following way. For each attractor appearing in the subgraph g_{best} (see description of f_T evaluation), split the network nodes in two groups G_1 and G_2 by comparing each node state (average over all states in the attractor) to the state expected from the training set. Group G_1 contains nodes that behaves as expected or do not appear in the training set while group G_2 contains the nodes that do not behave as expected. For each pair of nodes $(n_1, n_2) \in G_1 \times G_2$, add (n_1, n_2, s) to the list of possible paths, with $s = 1$ if n_2 state is lower (respectively higher) than expected and n_1 state is higher (respectively lower) than 0.5 and $s = -1$ if n_2 state is lower (respectively higher) than expected and n_1 state is lower (respectively higher) than 0.5.

  Randomly choose one element (n_1, n_2, s) in the list of possible paths (with uniform probability distribution). Randomly choose one path (with uniform probability distribution) among all elementary paths in the PKN connecting n_1 to n_2 with sign s and length smaller than or equal to the length of the shortest path from n_1 to n_2 in the PKN + 4. Add this path to the network. To search for elementary paths in the PKN, we use a slightly modified version of the algorithm proposed by Klamt and von Kamp [3] (Supplementary Information: algorithm 4).
- Remove node (priority $p_2 = 1$): Randomly remove one non-essential node from the network.

The type of mutation is randomly chosen with probability $p_i / \sum_j p_j$. After the mutation, isolated nodes (neither incoming nor outgoing edges) are removed from the network.

Cross-over:
Given two replicas, each edge appearing in at least one of the replicas is exchanged between the replicas with probability 0.5. When edges are exchanged, nodes are added if needed.

Evaluation of the network optimization method

Comparing a model network to the gold standard

To compare predictions of a model network and the gold standard network, a score (s_{all}) is defined in the following way:

1. Starting from each attractor of the gold standard network, we use boolSim [1] to evaluate the attractors reached by both the model network and the gold standard network after all single node perturbation of essential nodes (node state fixed to 0, node state fixed to 1 as well as unperturbed network). For an initial attractor a of the gold standard network and a single node perturbation p, we denote by $y_i^{(a,p)}$ and $\tilde{y}_i^{(a,p)}$ the average states of node i in the attractors reached by the model network and the gold standard network respectively. If the attractor reached by the model or the gold standard network contains more than one state, $y_i^{(n,k)}$ or $\tilde{y}_i^{(a,p)}$ contain the average node state over all states in the attractor. If more than one attractor is reached, $y_i^{(a,p)}$ or $\tilde{y}_i^{(a,p)}$ contain the average over all reached attractors.

2. For a given initial attractor a and perturbation p, we measure the Manhattan distance between the average states reached by the gold standard network and the model network:

$$\Delta_{a,p} = \sum_i |y_i^{(a,p)} - \tilde{y}_i^{(a,p)}|$$

where the sum runs over all essential nodes. If a node is missing in the model network, it is considered as isolated and its state is set to $y_i^{(a,p)} = 0$.

3. The total distance Δ between gold standard and model network predictions is then obtained as the sum of the distances obtained for all initial attractors and all perturbations:

$$\Delta = \sum_a \sum_p \Delta_{a,p}$$

This distance can then be transformed into a score

$$s_{all} = 1 - \frac{\Delta}{M \cdot N_{\text{ess.nodes}} \cdot (2N_{\text{ess.nodes}} + 1)}$$

where M is the number of attractors of the unperturbed gold standard network, $N_{\text{ess.nodes}}$ is the number of essential nodes and $(2N_{\text{ess.nodes}} + 1)$ is the number of single node perturbations ($N_{\text{ess.nodes}}$ perturbations with node state fixed to 0, $N_{\text{ess.nodes}}$ perturbations with node state fixed to 1 and 1 perturbation corresponding to unperturbed network).

Using this normalization, the resulting score s_{all} is between 0 (worst) and 1 (best). The score s_{all} is interpreted as a measure of the predictive power of the model network.

Combined predictions: variance and error

In the main paper, we summarize the predictions of multiple model networks by measuring their averages and variances, and we compare variances with errors. In this section, we will define these quantities more precisely. As in the previous section, we consider the average states reached by model networks and the gold standard network after all single node perturbations of essential nodes, starting from each attractor of the gold standard network. The same notations as above are used, except that since we have multiple model networks, the average state of node i in the attractors reached by n-th model network is denoted by $y_i^{(n,a,p)}$. Note that exact attractor reachability graphs are used here (evaluated with boolSim). For each initial attractor a, perturbation p and node i, we measure the average ($\mu_i^{(a,p)}$) and sample variance ($v_i^{(a,p)}$) of all model network predictions as:

$$\begin{aligned}
\mu_i^{(a,p)} &= \frac{1}{N_m} \sum_n y_i^{(n,a,p)} \\
v_i^{(a,p)} &= \frac{1}{N_m - 1} \sum_n \left(y_i^{(n,a,p)} - \mu_i^{(a,p)} \right)
\end{aligned}$$

where N_m is the number of model networks. The corresponding prediction error is defined as the absolute difference between average prediction of the model networks ($\mu_i^{(a,p)}$) and average state reached by the gold standard network ($\tilde{y}_i^{(a,p)}$):

$$\epsilon_i^{(a,p)} = \left| \mu_i^{(a,p)} - \tilde{y}_i^{(a,p)} \right|$$

Figure 10 in the main text presents the distribution of all 21112 points ($\epsilon_i^{(a,p)}, v_i^{(a,p)}$) obtained with all 4 initial attractors a, 29 perturbations p (14 nodes fixed to 0, 14 nodes fixed to 1, unperturbed network), 14 essential nodes i, and 13 input data sets (training set and PKN).

References

1. Garg A, Di Cara A, Xenarios I, Mendoza L, De Micheli G: **Synchronous versus asynchronous modeling of gene regulatory networks**. *Bioinformatics* 2008, **24**(17):1917–1925.

2. Weisstein EW: **Strongly Connected Component. From MathWorld—A Wolfram Web Resource**[http://mathworld.wolfram.com/StronglyConnectedComponent.html]. [Visited on 28/1/2016].

3. Klamt S, von Kamp A: **Computing paths and cycles in biological interaction graphs**. *BMC Bioinformatics* 2009, **10**:181.

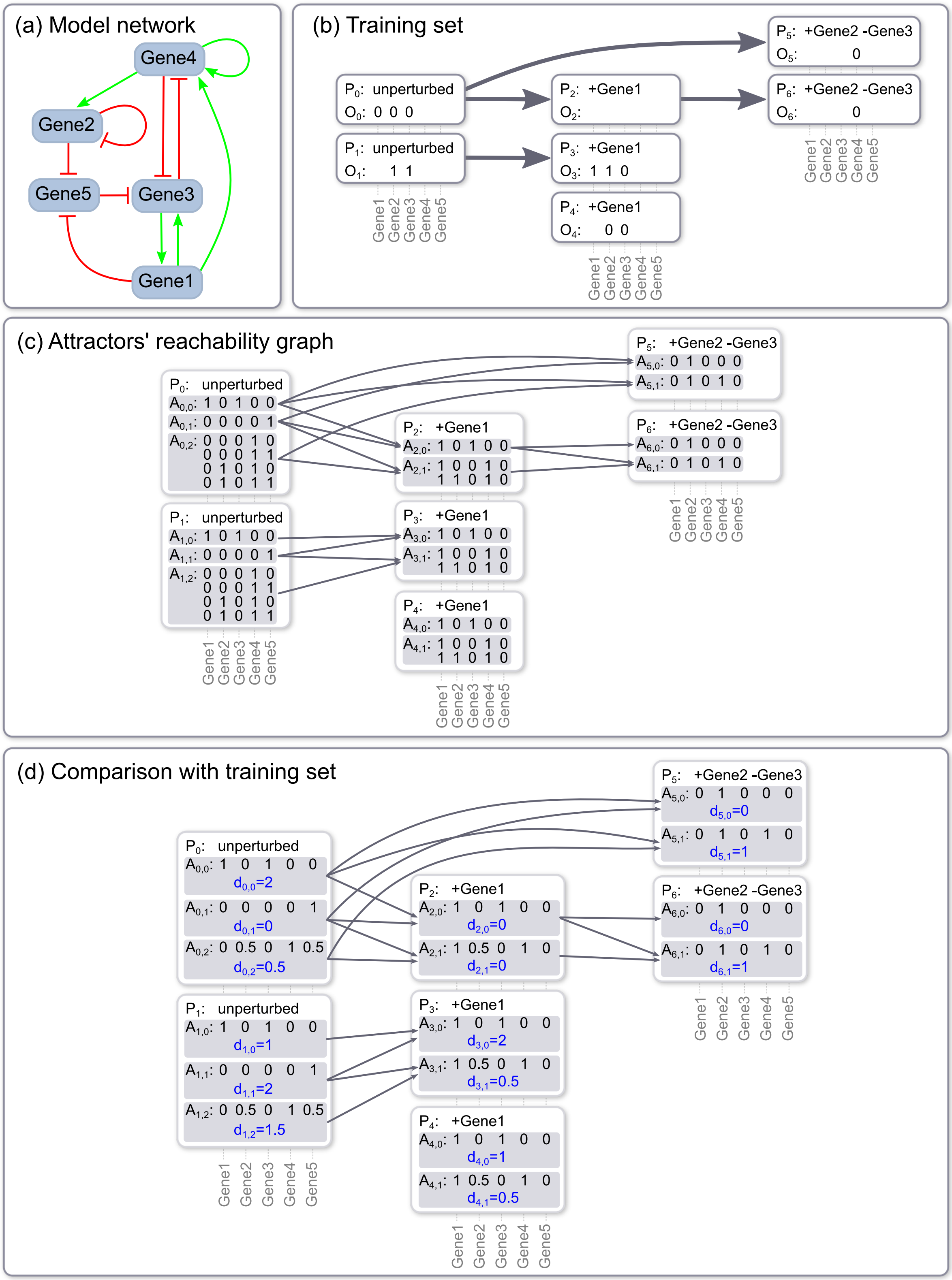

Figure S1: Sample model network (a) and training set graph (b) used to illustrate the evaluation of the first fitness function component f_T. (c) Asynchronous attractor reachability graph of the model network, with perturbations and transitions corresponding to the training set graph. (d) Each attractor with multiple states ($A_{0,2}$, $A_{1,2}$, $A_{2,1}$, $A_{3,1}$ and , $A_{4,1}$) is replaced by the average over all its states. Distances $d_{n,k}$ between each attractor and the corresponding observation from the training set are shown in blue.

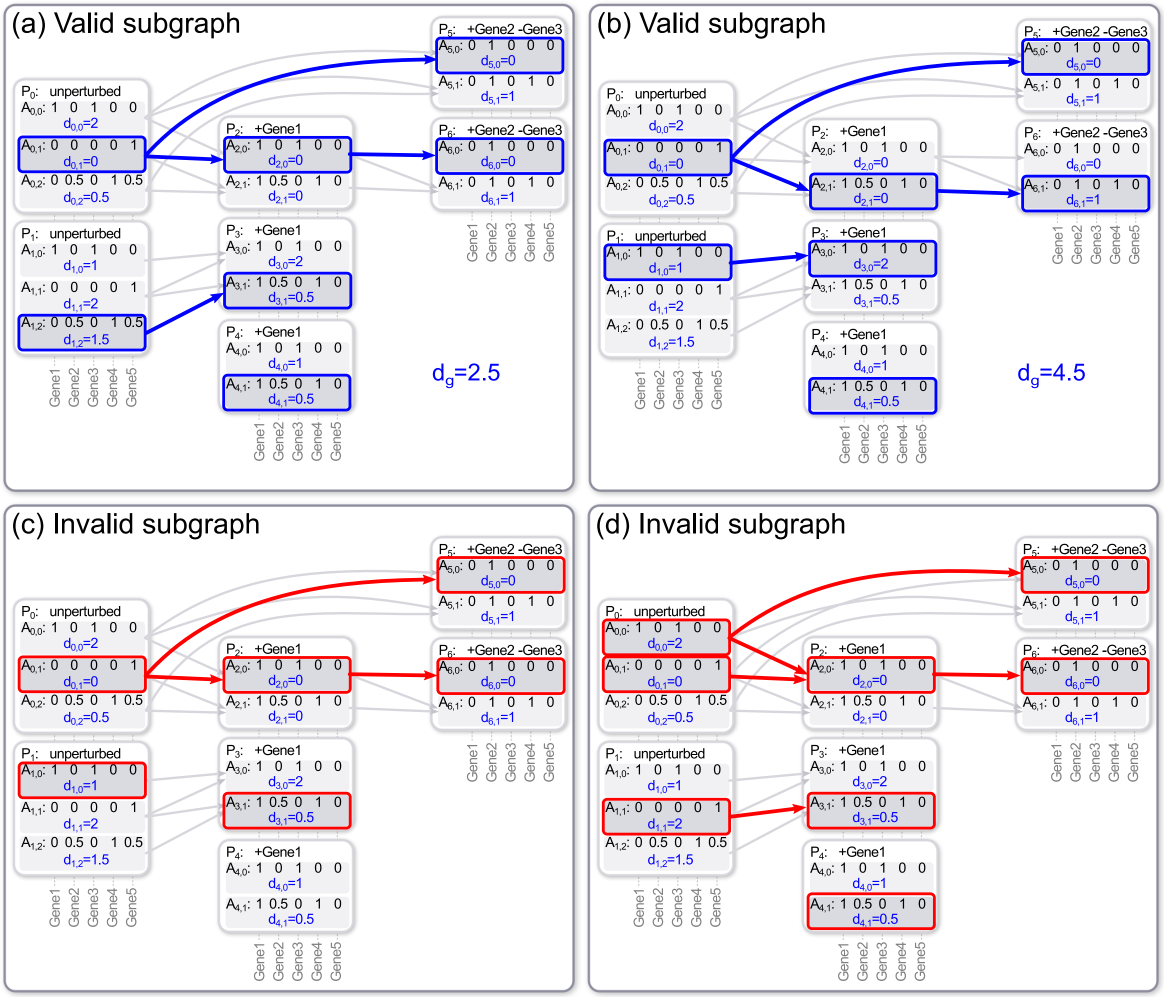

Figure S2: (a) and (b): Sample subgraphs (blue) of the attractor reachability graph with the same structure as the training set graph. The total distance to training set d_g is given in blue for each subgraph. With $d_g = 2.5$, this subgraph minimizes the total distance to the training set. Therefore in this example $f_T = d_g/N_{obs} = 2.5/12$. (c) and (d): Sample subgraphs (red) of the attractor reachability graph which do not have the same structure as the reachability graph. Subgraph (c) is not valid for two reasons: (i) Node (P_4, O_4) from the training set graph does not have a corresponding node in the subgraph. (ii) Edge $(P_1, O_1) \rightarrow (P_3, O_3)$ from the training set graph does not have a corresponding edge in the subgraph. Subgraph (d) is not valid for two reasons: (i) Node (P_0, O_0) from the training set graph has two (instead of one) corresponding nodes in the subgraph ($A_{0,0}$ and $A_{0,1}$). (ii) Edge $(P_0, O_0) \rightarrow (P_2, O_2)$ from the training set graph has two (instead of one) corresponding edges in the subgraph ($A_{0,0} \rightarrow A_{2,0}$ and $A_{0,1} \rightarrow A_{2,0}$).
