# Supplementary material for: Boolean regulatory network reconstruction using literature based knowledge with a genetic algorithm optimization method
Source: BMC Bioinformatics. 2016 Oct 6;17:410. doi: 10.1186/s12859-016-1287-z (PMC5053080; doi:10.1186/s12859-016-1287-z)
Supplement: Additional file 2: — Cell-fate decision model: in silico training sets. Training sets used to evaluate the optimization method. Each training set is given in the form of a transition graph, where each node contains a perturbation with the corresponding observation (stable phenotype) and edges denote a transition between stable phenotype upon change of condition (perturbation). Perturbations are specified using a combination of node names prefixed by – or + sign to specify knock-out (node state fixed to 0) or over-expression (node state fixed to 1). When multiple edges connect the same source node to different target nodes, it means that all transitions start from the same phenotype (same attractor of the network). Node states that were intentionally reversed to generate errors in training sets are shown in red. (PDF 9015 kb) [file 12859_2016_1287_MOESM2_ESM.pdf]

Cell-fate decision model: training set 1 (Large)

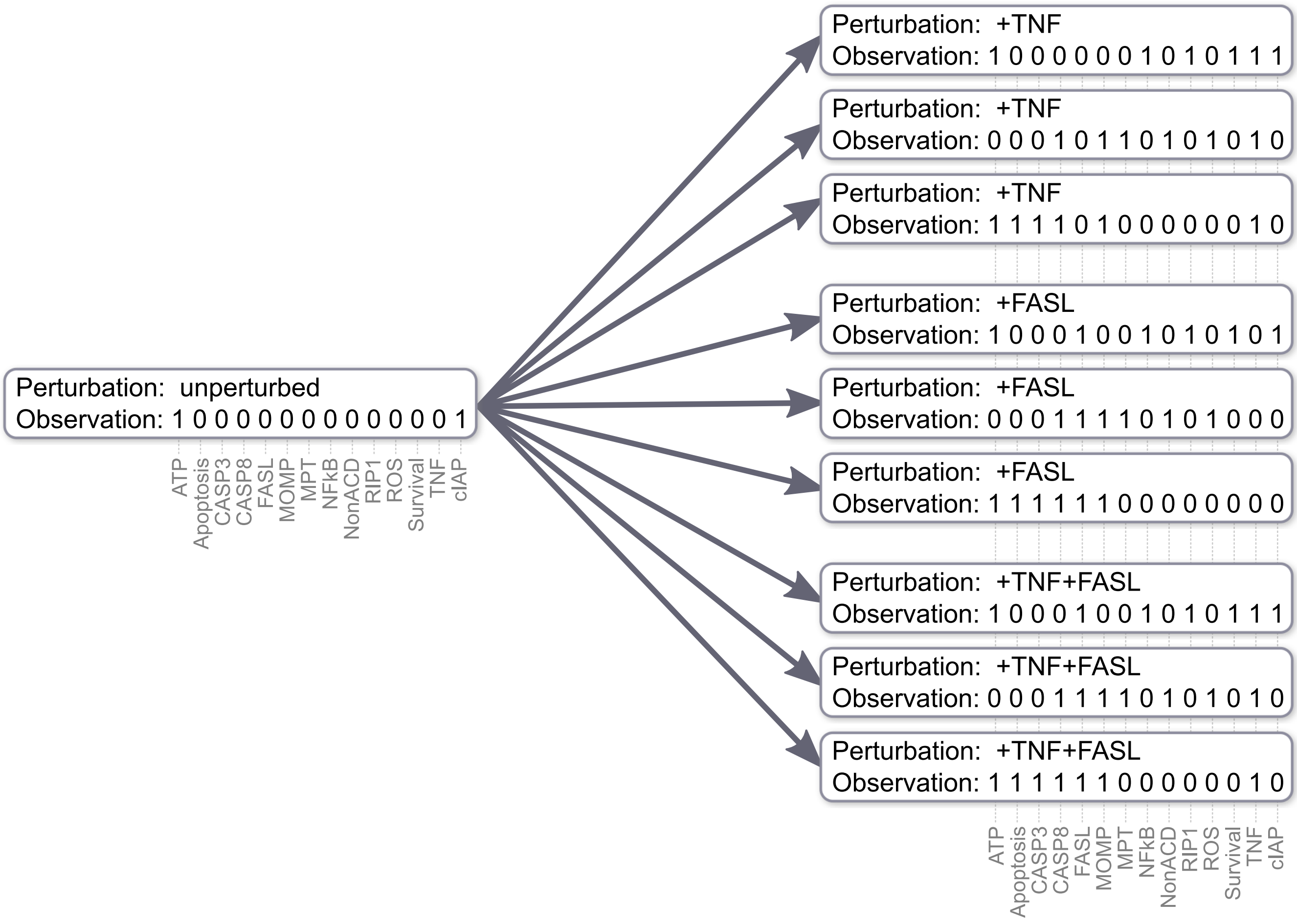

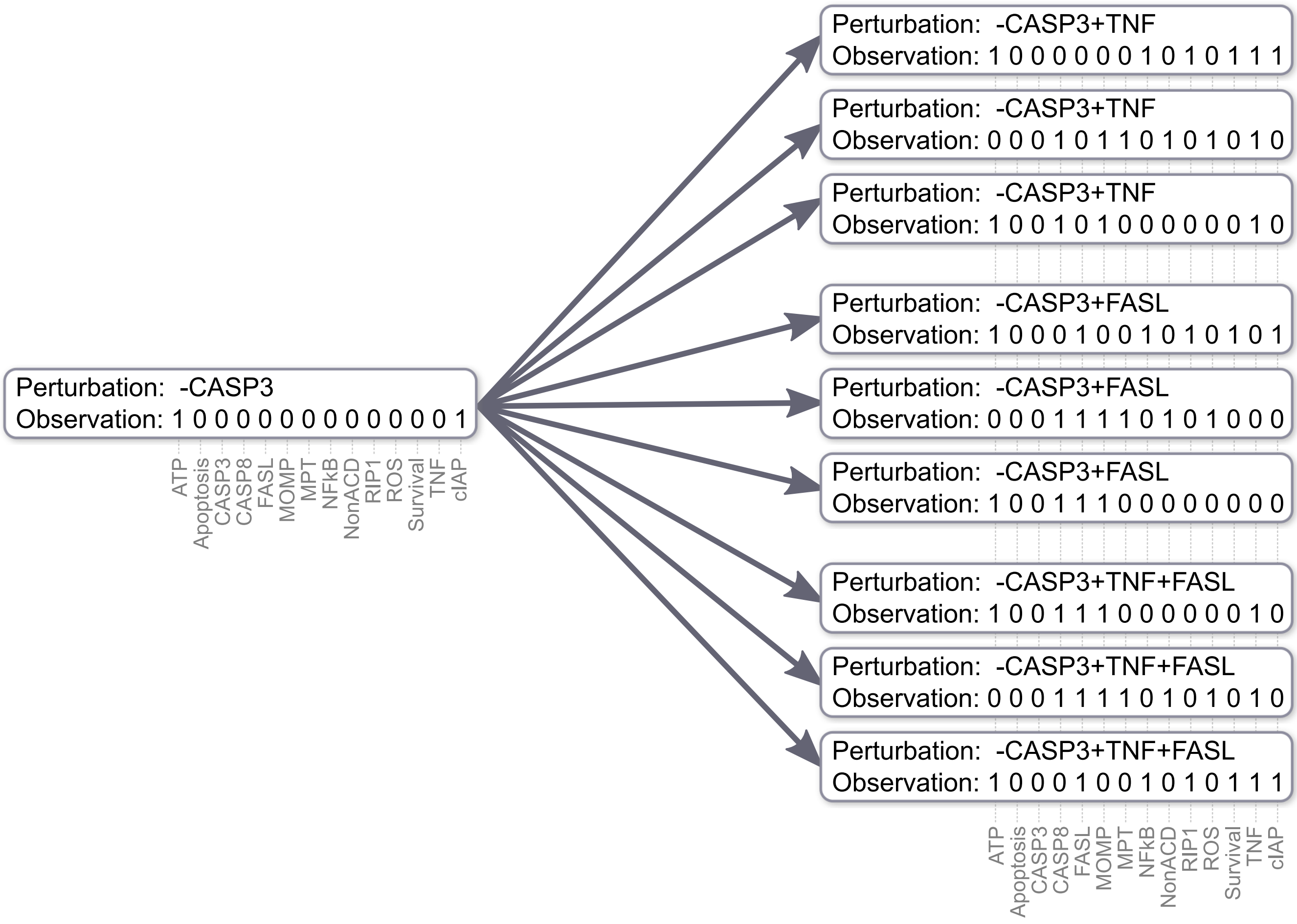

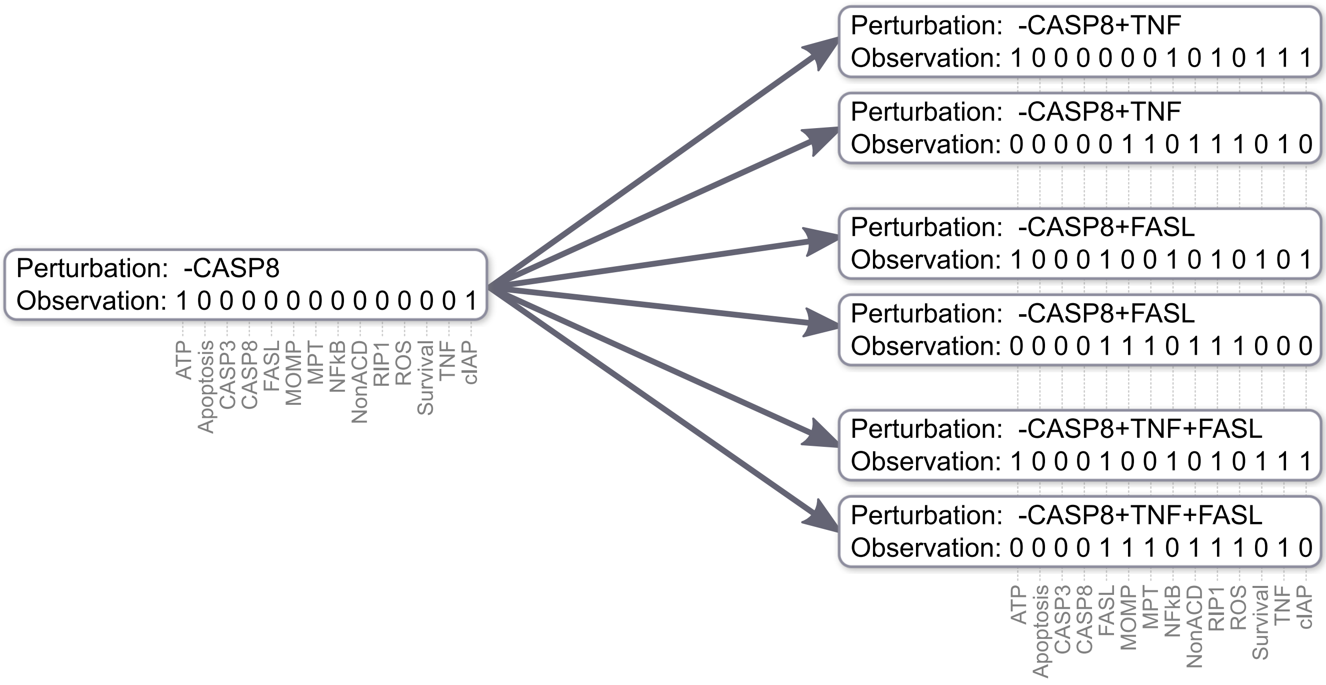
Perturbation: -CASP8
Observation: 1 0 0 0 0 0 0 0 0 0 0 0 0 0 1
ATP Apoptosis CASP3 CASP8 FASL MOMP MPT NFkB NonACD RIP1 ROS Survival TNF cIAP
Perturbation: -CASP8+TNF
Observation: 1 0 0 0 0 0 0 1 0 1 0 1 1 1
Perturbation: -CASP8+TNF
Observation: 0 0 0 0 0 1 1 0 1 1 1 0 1 0
Perturbation: -CASP8+FASL
Observation: 1 0 0 0 1 0 0 1 0 1 0 1 0 1
Perturbation: -CASP8+FASL
Observation: 0 0 0 0 1 1 1 0 1 1 1 0 0 0
Perturbation: -CASP8+TNF+FASL
Observation: 1 0 0 0 1 0 0 1 0 1 0 1 1 1
Perturbation: -CASP8+TNF+FASL
Observation: 0 0 0 0 1 1 1 0 1 1 1 0 1 0
ATP Apoptosis CASP3 CASP8 FASL MOMP MPT NFkB NonACD RIP1 ROS Survival TNF cIAP

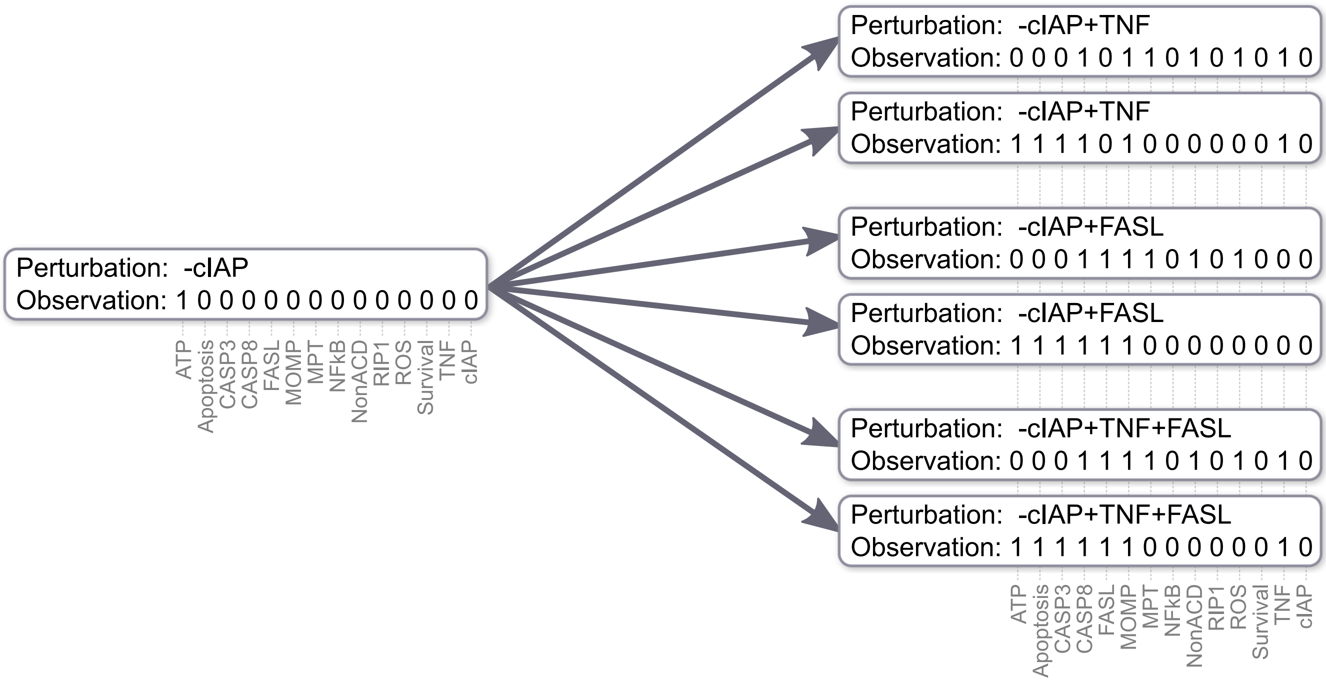
Perturbation: -cIAP
Observation: 1 0 0 0 0 0 0 0 0 0 0 0 0 0 0
ATP Apoptosis CASP3 CASP8 FASL MOMP MPT NFkB NonACD RIP1 ROS Survival TNF cIAP
Perturbation: -cIAP+TNF
Observation: 0 0 0 1 0 1 1 0 1 0 1 0 1 0
Perturbation: -cIAP+TNF
Observation: 1 1 1 1 0 1 0 0 0 0 0 0 1 0
Perturbation: -cIAP+FASL
Observation: 0 0 0 1 1 1 1 0 1 0 1 0 0 0
Perturbation: -cIAP+FASL
Observation: 1 1 1 1 1 1 0 0 0 0 0 0 0 0
Perturbation: -cIAP+TNF+FASL
Observation: 0 0 0 1 1 1 1 0 1 0 1 0 1 0
Perturbation: -cIAP+TNF+FASL
Observation: 1 1 1 1 1 1 0 0 0 0 0 0 1 0
ATP Apoptosis CASP3 CASP8 FASL MOMP MPT NFkB NonACD RIP1 ROS Survival TNF cIAP

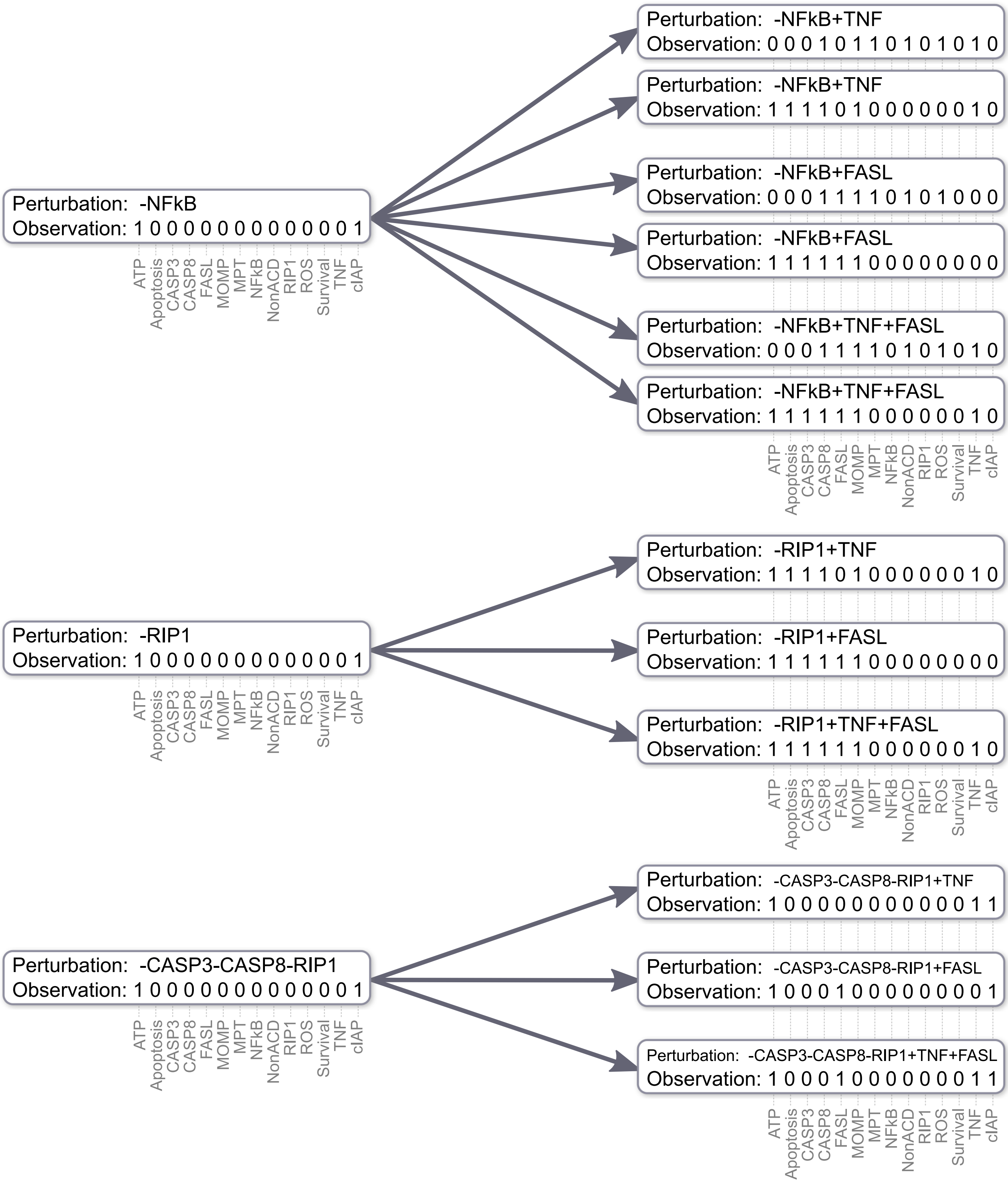

Perturbation: -NFkB
Observation: 1 0 0 0 0 0 0 0 0 0 0 0 0 1
ATP Apoptosis CASP3 CASP8 FASL MOMP MPT NFkB NonACD RIP1 ROS Survival TNF cIAP
Perturbation: -NFkB+TNF
Observation: 0 0 0 1 0 1 1 0 1 0 1 0 1 0
Perturbation: -NFkB+TNF
Observation: 1 1 1 1 0 1 0 0 0 0 0 0 1 0
Perturbation: -NFkB+FASL
Observation: 0 0 0 1 1 1 1 0 1 0 1 0 0 0
Perturbation: -NFkB+FASL
Observation: 1 1 1 1 1 1 0 0 0 0 0 0 0 0
Perturbation: -NFkB+TNF+FASL
Observation: 0 0 0 1 1 1 1 0 1 0 1 0 1 0
Perturbation: -NFkB+TNF+FASL
Observation: 1 1 1 1 1 1 0 0 0 0 0 0 1 0
ATP Apoptosis CASP3 CASP8 FASL MOMP MPT NFkB NonACD RIP1 ROS Survival TNF cIAP
Perturbation: -RIP1
Observation: 1 0 0 0 0 0 0 0 0 0 0 0 0 1
ATP Apoptosis CASP3 CASP8 FASL MOMP MPT NFkB NonACD RIP1 ROS Survival TNF cIAP
Perturbation: -RIP1+TNF
Observation: 1 1 1 1 0 1 0 0 0 0 0 0 1 0
Perturbation: -RIP1+FASL
Observation: 1 1 1 1 1 1 0 0 0 0 0 0 0 0
Perturbation: -RIP1+TNF+FASL
Observation: 1 1 1 1 1 1 0 0 0 0 0 0 1 0
ATP Apoptosis CASP3 CASP8 FASL MOMP MPT NFkB NonACD RIP1 ROS Survival TNF cIAP
Perturbation: -CASP3-CASP8-RIP1
Observation: 1 0 0 0 0 0 0 0 0 0 0 0 0 1
ATP Apoptosis CASP3 CASP8 FASL MOMP MPT NFkB NonACD RIP1 ROS Survival TNF cIAP
Perturbation: -CASP3-CASP8-RIP1+TNF
Observation: 1 0 0 0 0 0 0 0 0 0 0 0 1 1
Perturbation: -CASP3-CASP8-RIP1+FASL
Observation: 1 0 0 0 1 0 0 0 0 0 0 0 0 1
Perturbation: -CASP3-CASP8-RIP1+TNF+FASL
Observation: 1 0 0 0 1 0 0 0 0 0 0 0 1 1
ATP Apoptosis CASP3 CASP8 FASL MOMP MPT NFkB NonACD RIP1 ROS Survival TNF cIAP

Cell-fate decision model: training set 2 (Medium)

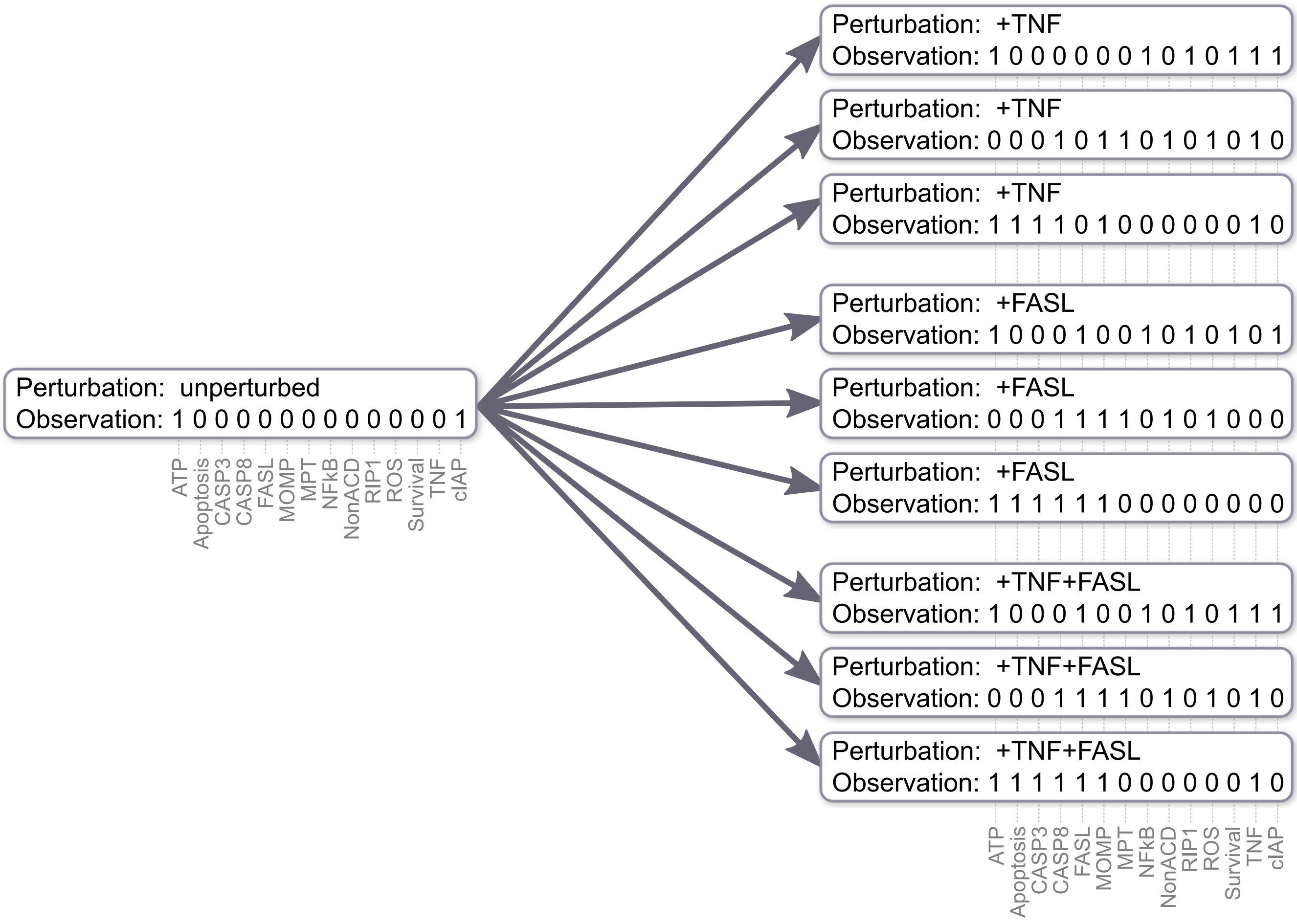

Cell-fate decision model: training set 3 (Small)

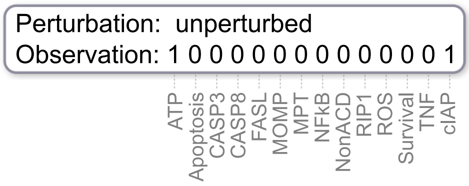

Cell-fate decision model: training set 1 (Large) with 10% error

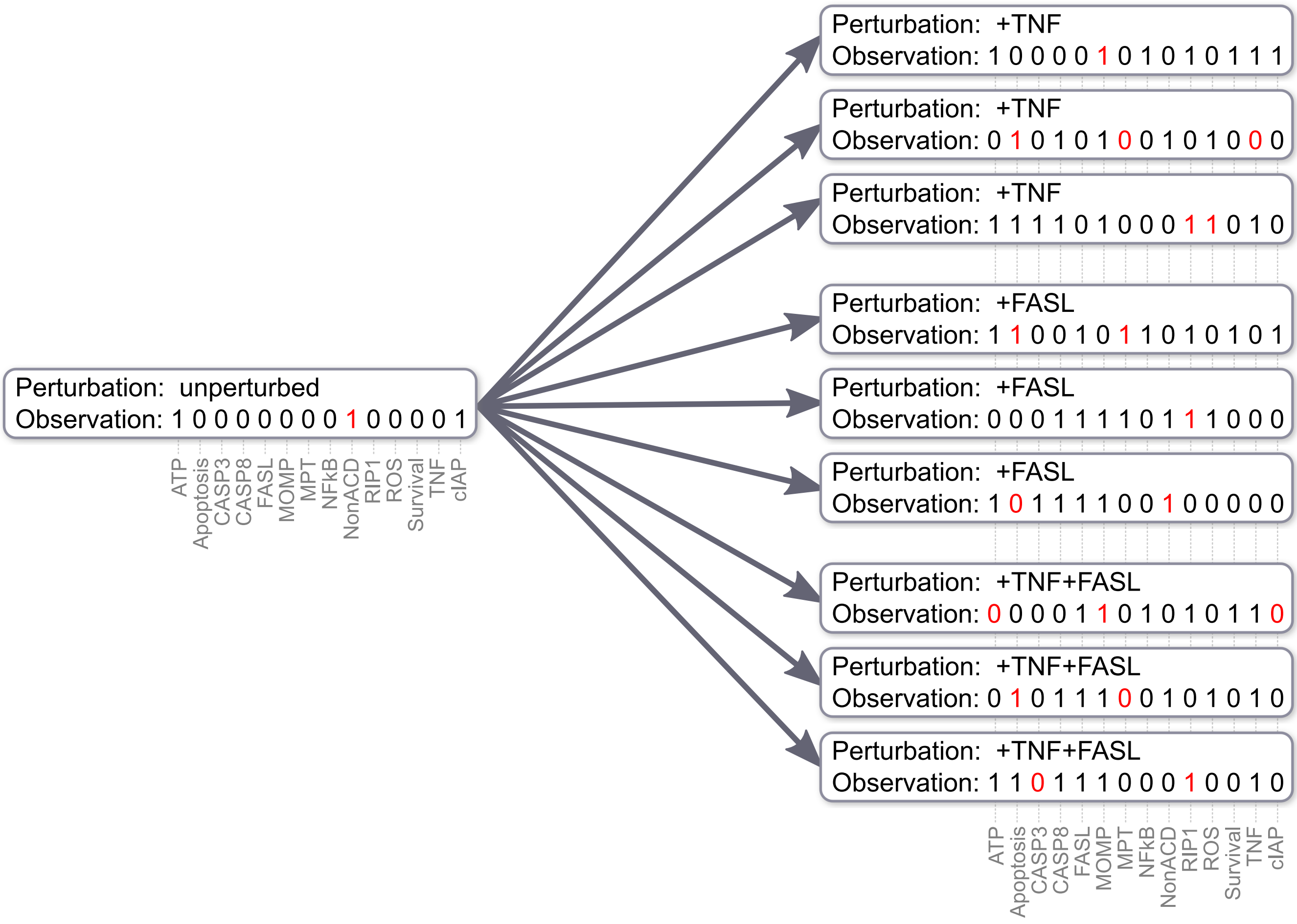

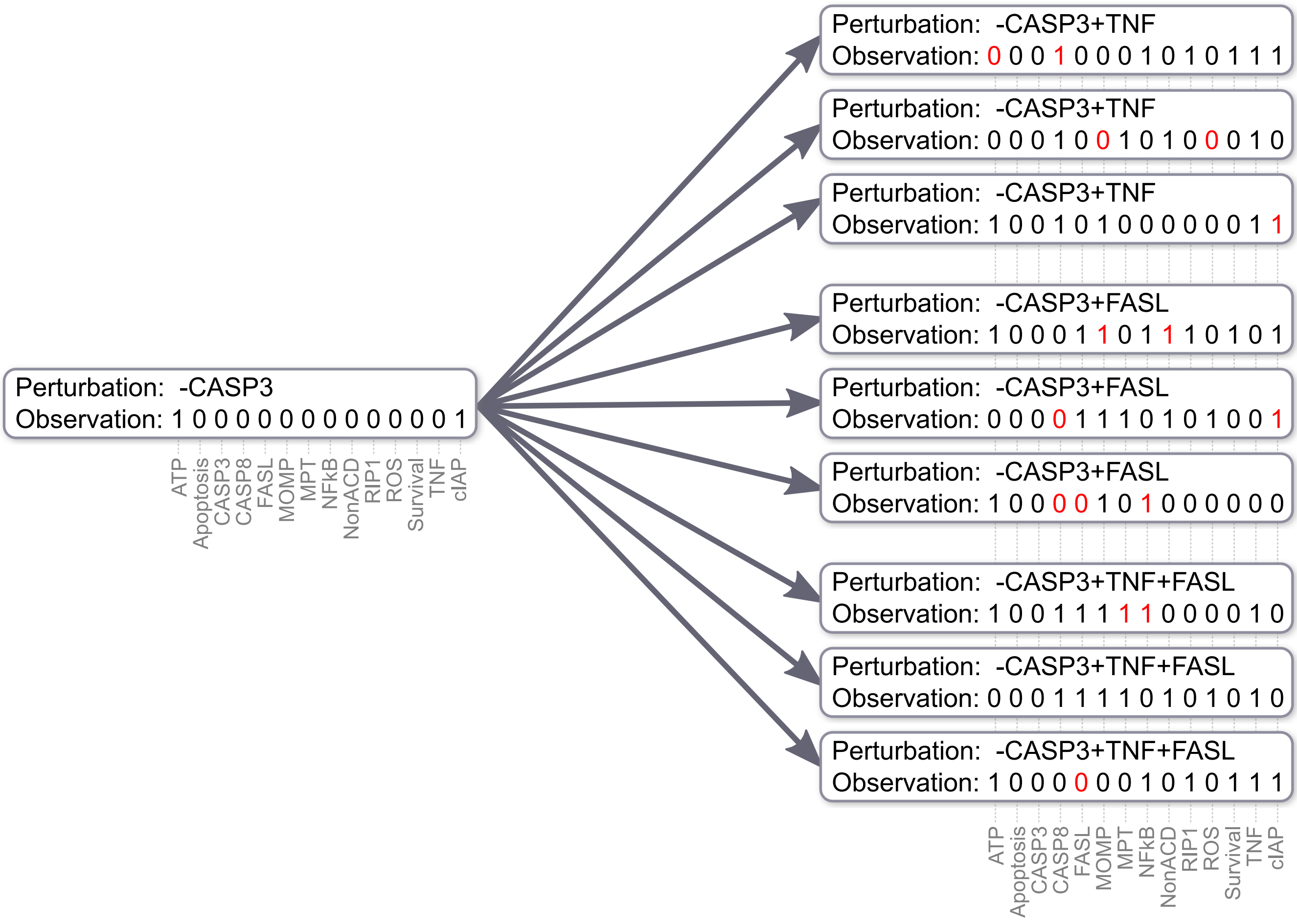

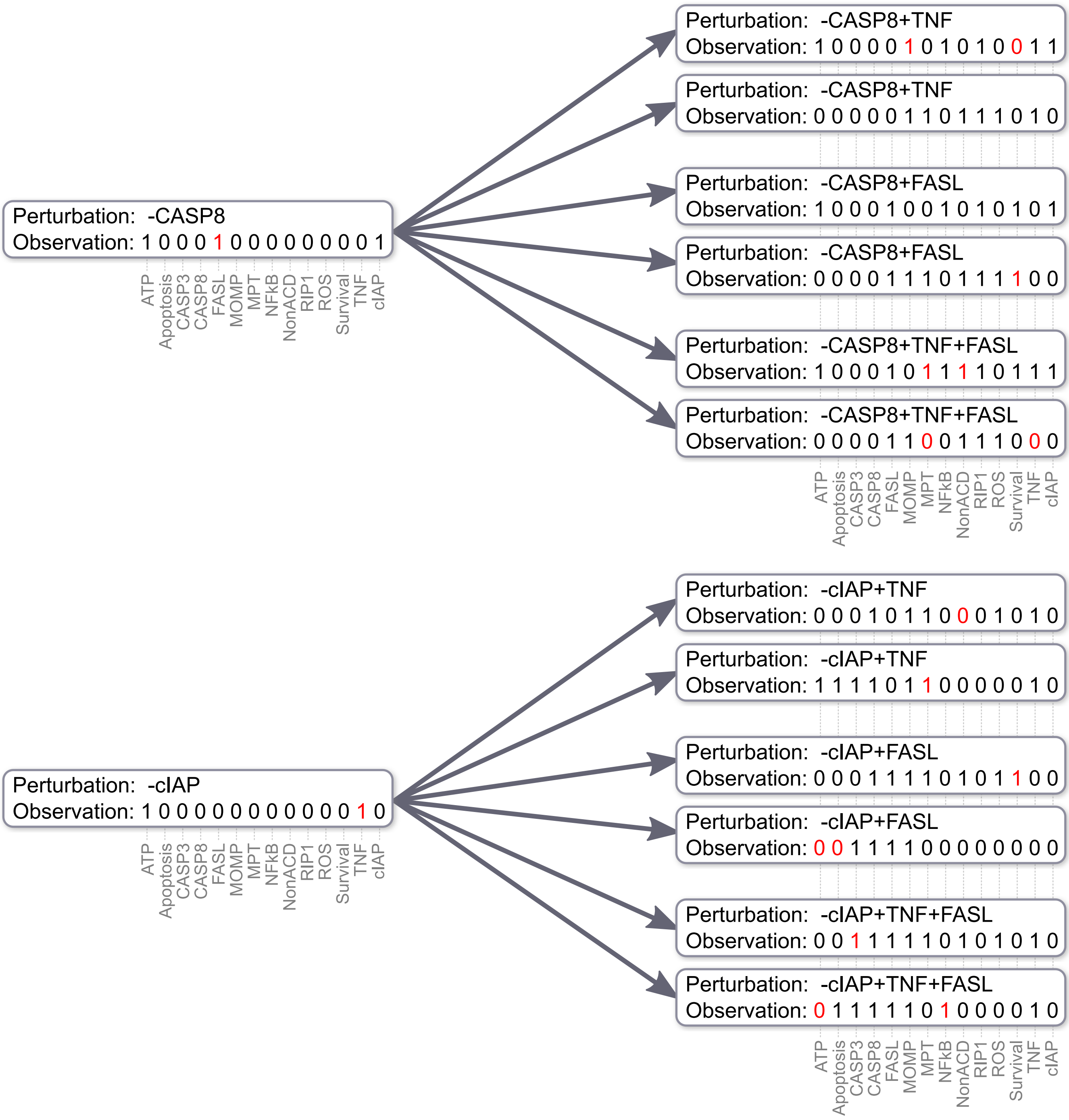

Perturbation: -CASP8
Observation: 1 0 0 0 1 0 0 0 0 0 0 0 0 1
ATP Apoptosis CASP3 CASP8 FASL MOMP MPT NFkB NonACD RIP1 ROS Survival TNF cIAP
Perturbation: -CASP8+TNF
Observation: 1 0 0 0 0 1 0 1 0 1 0 0 1 1
Perturbation: -CASP8+TNF
Observation: 0 0 0 0 0 1 1 0 1 1 1 0 1 0
Perturbation: -CASP8+FASL
Observation: 1 0 0 0 1 0 0 1 0 1 0 1 0 1
Perturbation: -CASP8+FASL
Observation: 0 0 0 0 1 1 1 0 1 1 1 1 0 0
Perturbation: -CASP8+TNF+FASL
Observation: 1 0 0 0 1 0 1 1 1 1 0 1 1 1
Perturbation: -CASP8+TNF+FASL
Observation: 0 0 0 0 1 1 0 0 1 1 1 0 0 0
ATP Apoptosis CASP3 CASP8 FASL MOMP MPT NFkB NonACD RIP1 ROS Survival TNF cIAP
Perturbation: -cIAP
Observation: 1 0 0 0 0 0 0 0 0 0 0 0 1 0
ATP Apoptosis CASP3 CASP8 FASL MOMP MPT NFkB NonACD RIP1 ROS Survival TNF cIAP
Perturbation: -cIAP+TNF
Observation: 0 0 0 1 0 1 1 0 0 0 1 0 1 0
Perturbation: -cIAP+TNF
Observation: 1 1 1 1 0 1 1 0 0 0 0 0 1 0
Perturbation: -cIAP+FASL
Observation: 0 0 0 1 1 1 1 0 1 0 1 1 0 0
Perturbation: -cIAP+FASL
Observation: 0 0 1 1 1 1 0 0 0 0 0 0 0 0
Perturbation: -cIAP+TNF+FASL
Observation: 0 0 1 1 1 1 1 0 1 0 1 0 1 0
Perturbation: -cIAP+TNF+FASL
Observation: 0 1 1 1 1 1 0 1 0 0 0 0 1 0
ATP Apoptosis CASP3 CASP8 FASL MOMP MPT NFkB NonACD RIP1 ROS Survival TNF cIAP

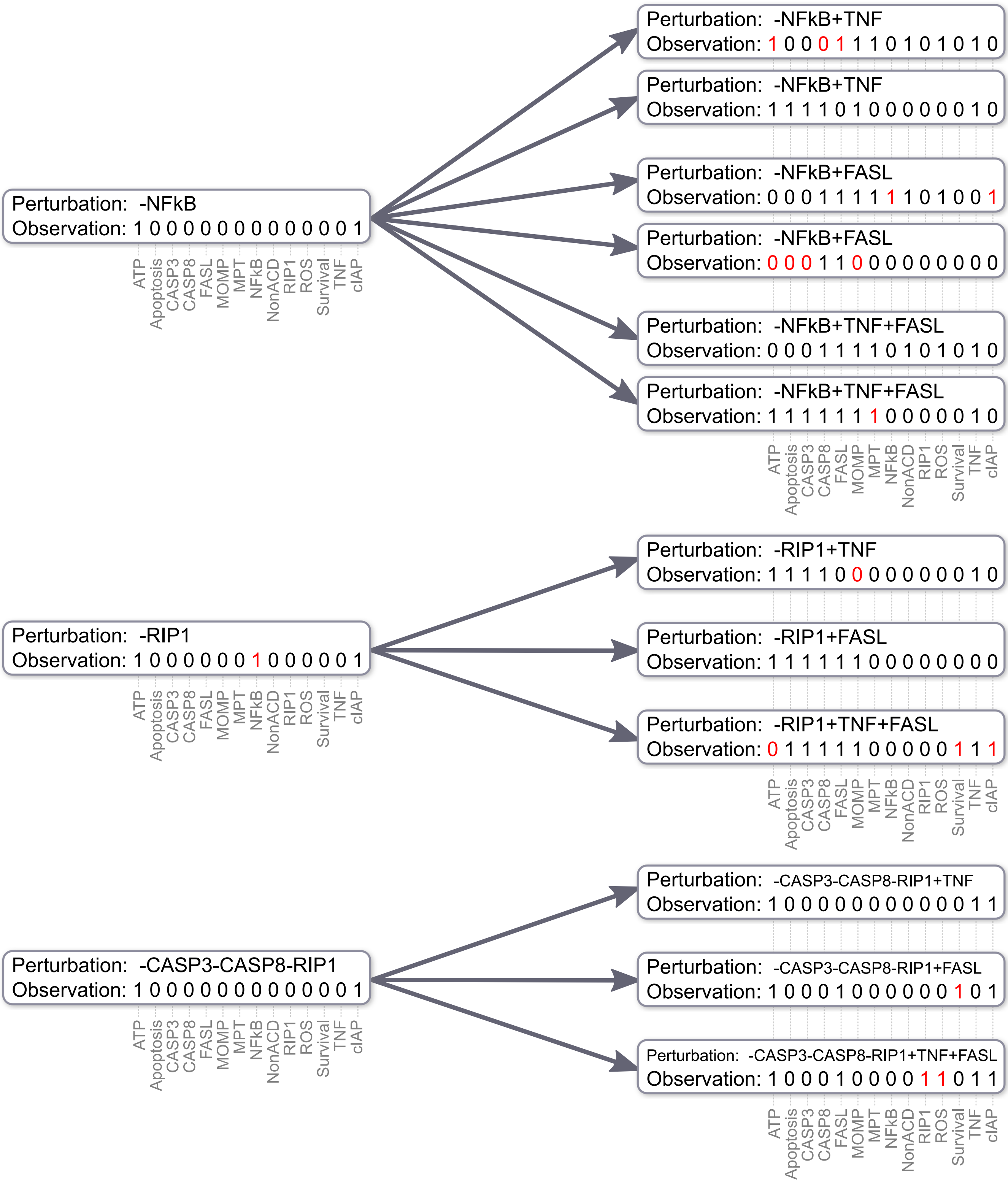

Perturbation: -NFkB
Observation: 1 0 0 0 0 0 0 0 0 0 0 0 0 1
ATP Apoptosis CASP3 CASP8 FASL MOMP MPT NFkB NonACD RIP1 ROS Survival TNF cIAP
Perturbation: -NFkB+TNF
Observation: 1 0 0 0 1 1 1 0 1 0 1 0 1 0
Perturbation: -NFkB+TNF
Observation: 1 1 1 1 0 1 0 0 0 0 0 0 1 0
Perturbation: -NFkB+FASL
Observation: 0 0 0 1 1 1 1 1 1 0 1 0 0 1
Perturbation: -NFkB+FASL
Observation: 0 0 0 1 1 0 0 0 0 0 0 0 0 0
Perturbation: -NFkB+TNF+FASL
Observation: 0 0 0 1 1 1 1 0 1 0 1 0 1 0
Perturbation: -NFkB+TNF+FASL
Observation: 1 1 1 1 1 1 1 0 0 0 0 0 1 0
ATP Apoptosis CASP3 CASP8 FASL MOMP MPT NFkB NonACD RIP1 ROS Survival TNF cIAP
Perturbation: -RIP1
Observation: 1 0 0 0 0 0 0 1 0 0 0 0 0 1
ATP Apoptosis CASP3 CASP8 FASL MOMP MPT NFkB NonACD RIP1 ROS Survival TNF cIAP
Perturbation: -RIP1+TNF
Observation: 1 1 1 1 0 0 0 0 0 0 0 0 1 0
Perturbation: -RIP1+FASL
Observation: 1 1 1 1 1 1 0 0 0 0 0 0 0 0
Perturbation: -RIP1+TNF+FASL
Observation: 0 1 1 1 1 1 0 0 0 0 0 1 1 1
ATP Apoptosis CASP3 CASP8 FASL MOMP MPT NFkB NonACD RIP1 ROS Survival TNF cIAP
Perturbation: -CASP3-CASP8-RIP1
Observation: 1 0 0 0 0 0 0 0 0 0 0 0 0 1
ATP Apoptosis CASP3 CASP8 FASL MOMP MPT NFkB NonACD RIP1 ROS Survival TNF cIAP
Perturbation: -CASP3-CASP8-RIP1+TNF
Observation: 1 0 0 0 0 0 0 0 0 0 0 0 1 1
Perturbation: -CASP3-CASP8-RIP1+FASL
Observation: 1 0 0 0 1 0 0 0 0 0 0 1 0 1
Perturbation: -CASP3-CASP8-RIP1+TNF+FASL
Observation: 1 0 0 0 1 0 0 0 0 1 1 0 1 1
ATP Apoptosis CASP3 CASP8 FASL MOMP MPT NFkB NonACD RIP1 ROS Survival TNF cIAP

Cell-fate decision model: training set 1 (Large) with 20% error

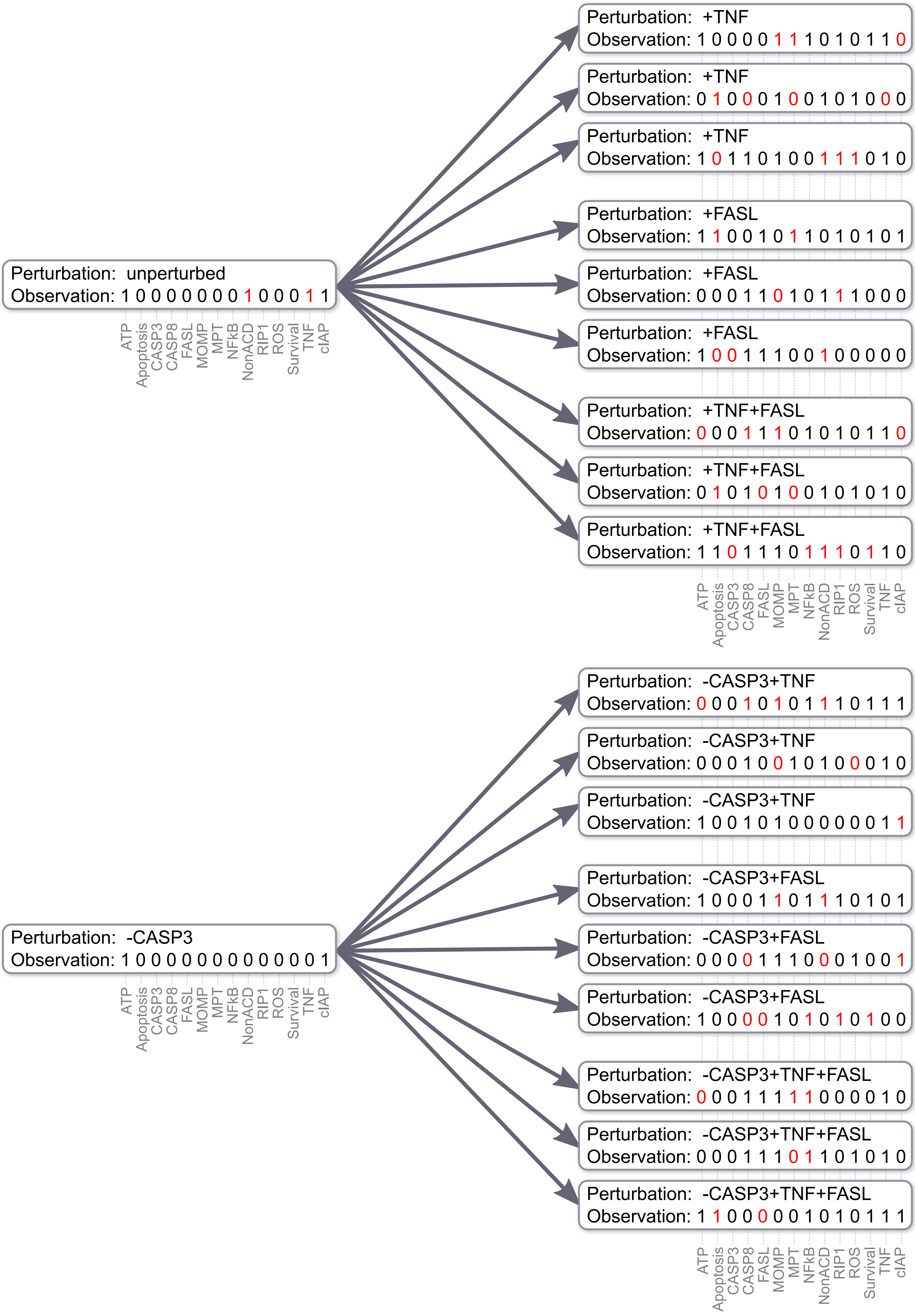

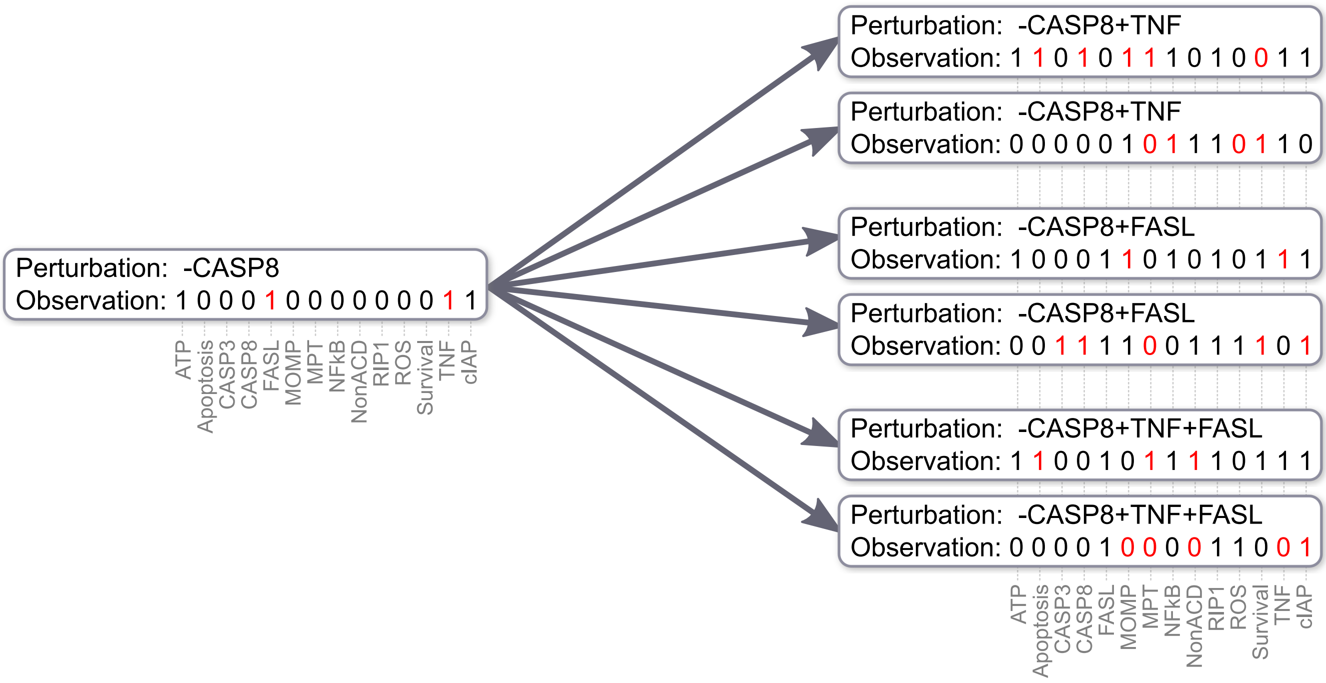
Perturbation: -CASP8
Observation: 1 0 0 0 1 0 0 0 0 0 0 0 1 1
ATP Apoptosis CASP3 CASP8 FASL MOMP MPT NFkB NonACD RIP1 ROS Survival TNF cIAP
Perturbation: -CASP8+TNF
Observation: 1 1 0 1 0 1 1 1 0 1 0 0 1 1
Perturbation: -CASP8+TNF
Observation: 0 0 0 0 0 1 0 1 1 1 0 1 1 0
Perturbation: -CASP8+FASL
Observation: 1 0 0 0 1 1 0 1 0 1 0 1 1 1
Perturbation: -CASP8+FASL
Observation: 0 0 1 1 1 1 0 0 1 1 1 1 0 1
Perturbation: -CASP8+TNF+FASL
Observation: 1 1 0 0 1 0 1 1 1 1 0 1 1 1
Perturbation: -CASP8+TNF+FASL
Observation: 0 0 0 0 1 0 0 0 0 1 1 0 0 1
ATP Apoptosis CASP3 CASP8 FASL MOMP MPT NFkB NonACD RIP1 ROS Survival TNF cIAP

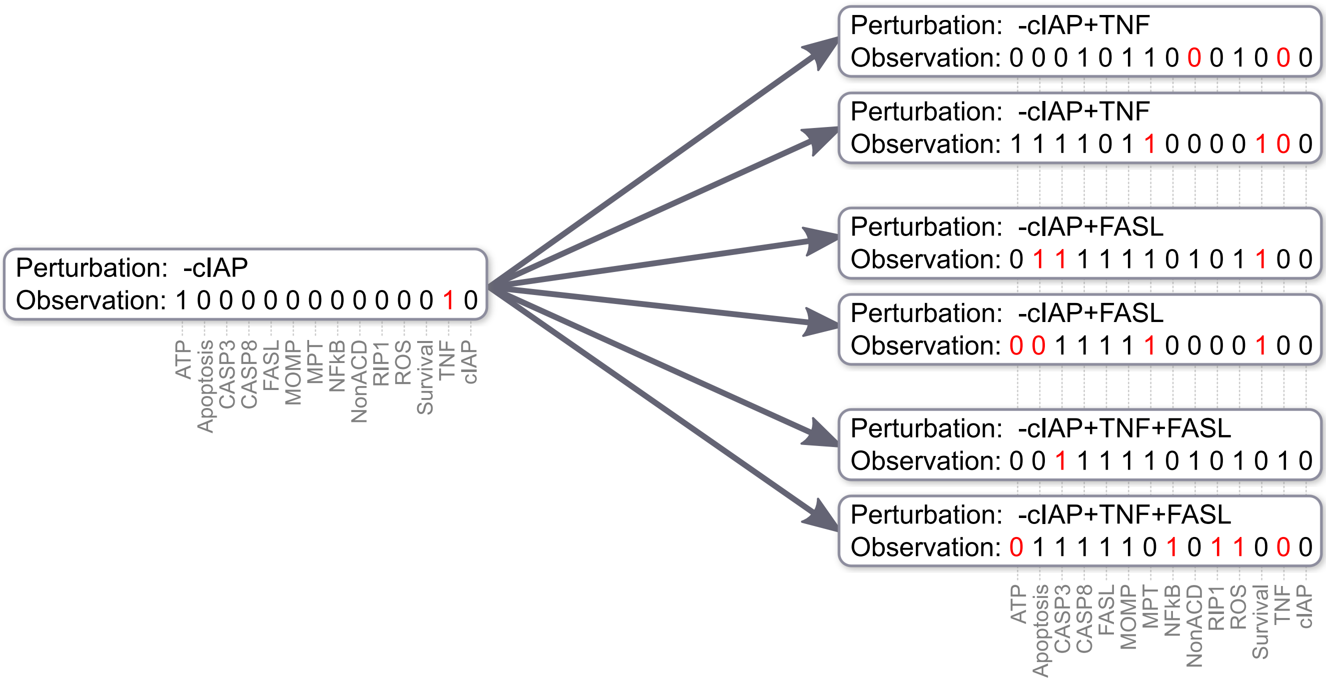
Perturbation: -cIAP
Observation: 1 0 0 0 0 0 0 0 0 0 0 0 1 0
ATP Apoptosis CASP3 CASP8 FASL MOMP MPT NFkB NonACD RIP1 ROS Survival TNF cIAP
Perturbation: -cIAP+TNF
Observation: 0 0 0 1 0 1 1 0 0 0 1 0 0 0
Perturbation: -cIAP+TNF
Observation: 1 1 1 1 0 1 1 0 0 0 0 1 0 0
Perturbation: -cIAP+FASL
Observation: 0 1 1 1 1 1 1 0 1 0 1 1 0 0
Perturbation: -cIAP+FASL
Observation: 0 0 1 1 1 1 1 0 0 0 0 1 0 0
Perturbation: -cIAP+TNF+FASL
Observation: 0 0 1 1 1 1 1 0 1 0 1 0 1 0
Perturbation: -cIAP+TNF+FASL
Observation: 0 1 1 1 1 1 0 1 0 1 1 0 0 0
ATP Apoptosis CASP3 CASP8 FASL MOMP MPT NFkB NonACD RIP1 ROS Survival TNF cIAP

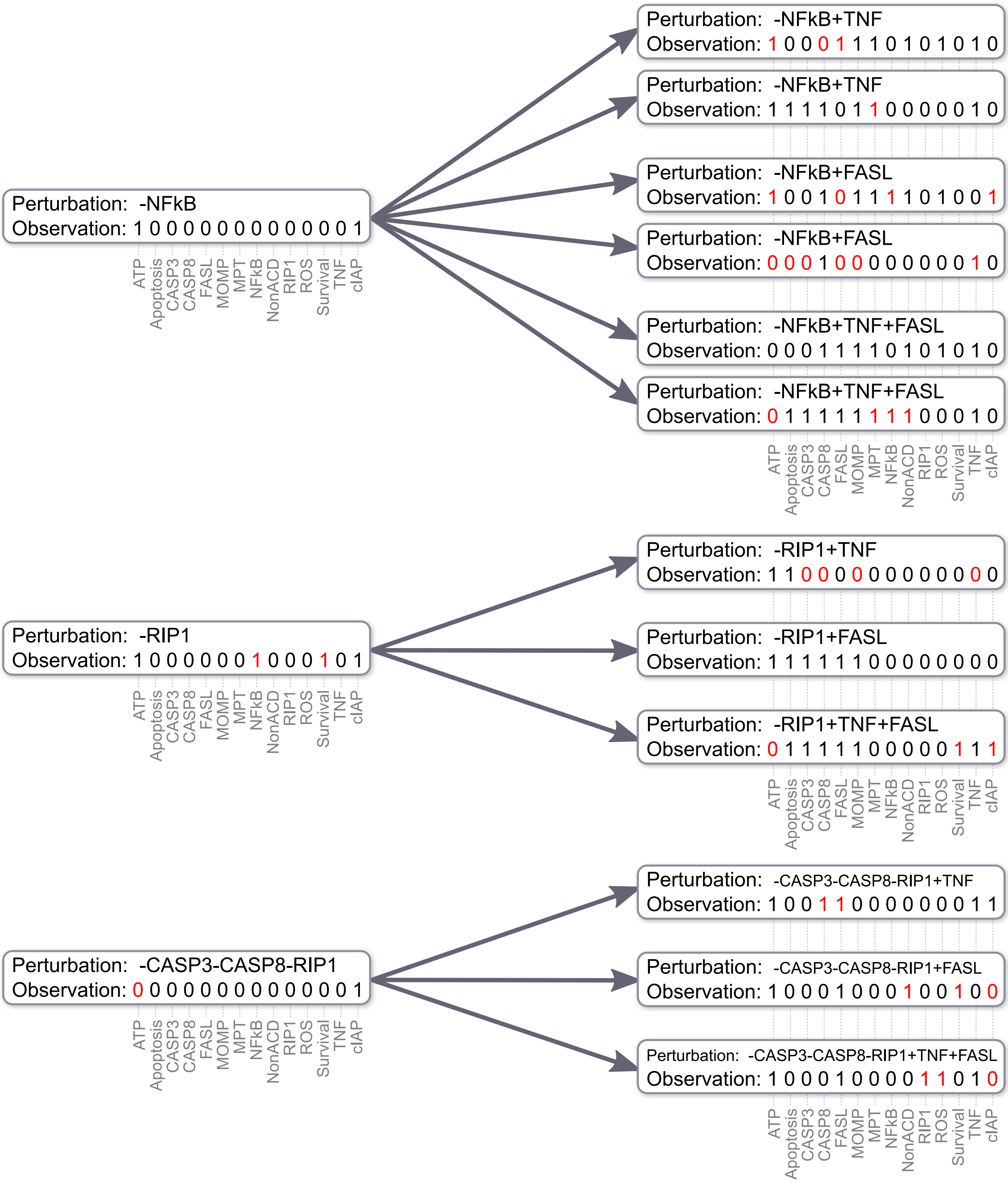
Perturbation: -NFkB
Observation: 1 0 0 0 0 0 0 0 0 0 0 0 0 1
Perturbation: -NFkB+TNF
Observation: 1 0 0 0 1 1 1 0 1 0 1 0 1 0
Perturbation: -NFkB+TNF
Observation: 1 1 1 1 0 1 1 0 0 0 0 0 1 0
Perturbation: -NFkB+FASL
Observation: 1 0 0 1 0 1 1 1 1 0 1 0 0 1
Perturbation: -NFkB+FASL
Observation: 0 0 0 1 0 0 0 0 0 0 0 0 1 0
Perturbation: -NFkB+TNF+FASL
Observation: 0 0 0 1 1 1 1 0 1 0 1 0 1 0
Perturbation: -NFkB+TNF+FASL
Observation: 0 1 1 1 1 1 1 1 1 0 0 0 1 0
Perturbation: -RIP1
Observation: 1 0 0 0 0 0 0 1 0 0 0 1 0 1
Perturbation: -RIP1+TNF
Observation: 1 1 0 0 0 0 0 0 0 0 0 0 0 0
Perturbation: -RIP1+FASL
Observation: 1 1 1 1 1 1 0 0 0 0 0 0 0 0
Perturbation: -RIP1+TNF+FASL
Observation: 0 1 1 1 1 1 0 0 0 0 0 1 1 1
Perturbation: -CASP3-CASP8-RIP1
Observation: 0 0 0 0 0 0 0 0 0 0 0 0 0 1
Perturbation: -CASP3-CASP8-RIP1+TNF
Observation: 1 0 0 1 1 0 0 0 0 0 0 0 1 1
Perturbation: -CASP3-CASP8-RIP1+FASL
Observation: 1 0 0 0 1 0 0 0 1 0 0 1 0 0
Perturbation: -CASP3-CASP8-RIP1+TNF+FASL
Observation: 1 0 0 0 1 0 0 0 0 1 1 0 1 0
ATP
Apoptosis
CASP3
CASP8
FASL
MOMP
MPT
NFkB
NonACD
RIP1
ROS
Survival
TNF
cIAP

Cell-fate decision model: training set 1 (Large) with 30% error

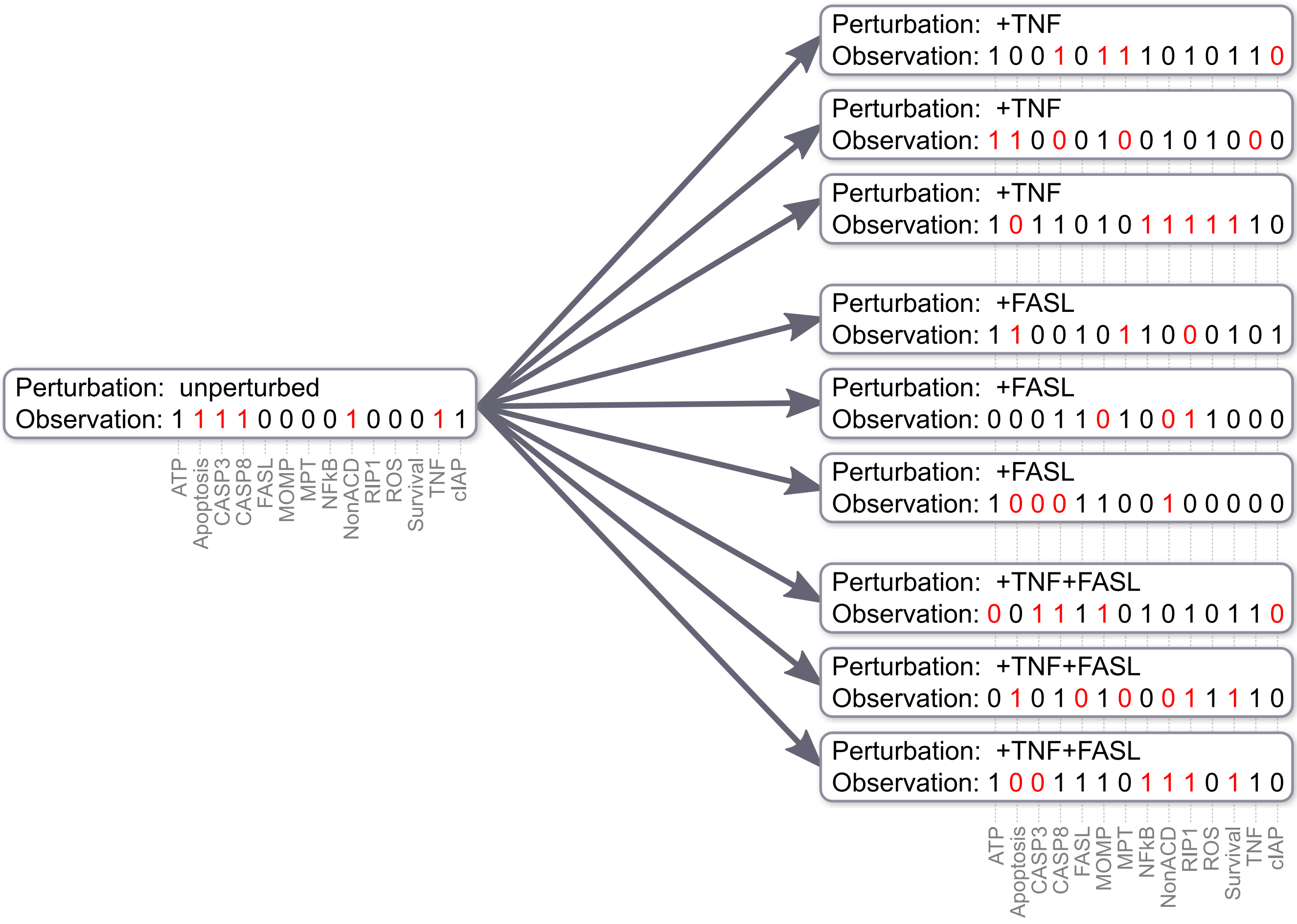

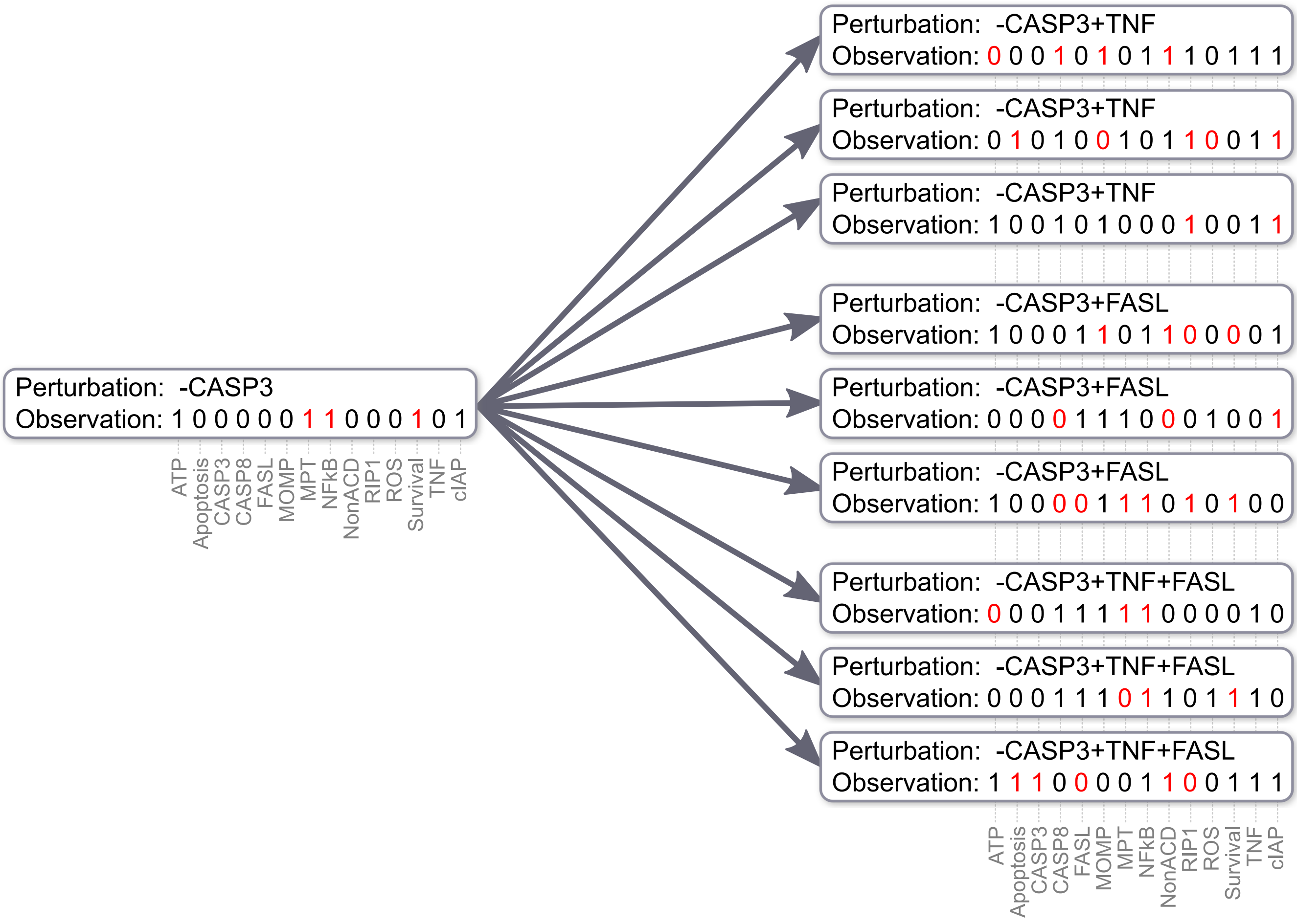

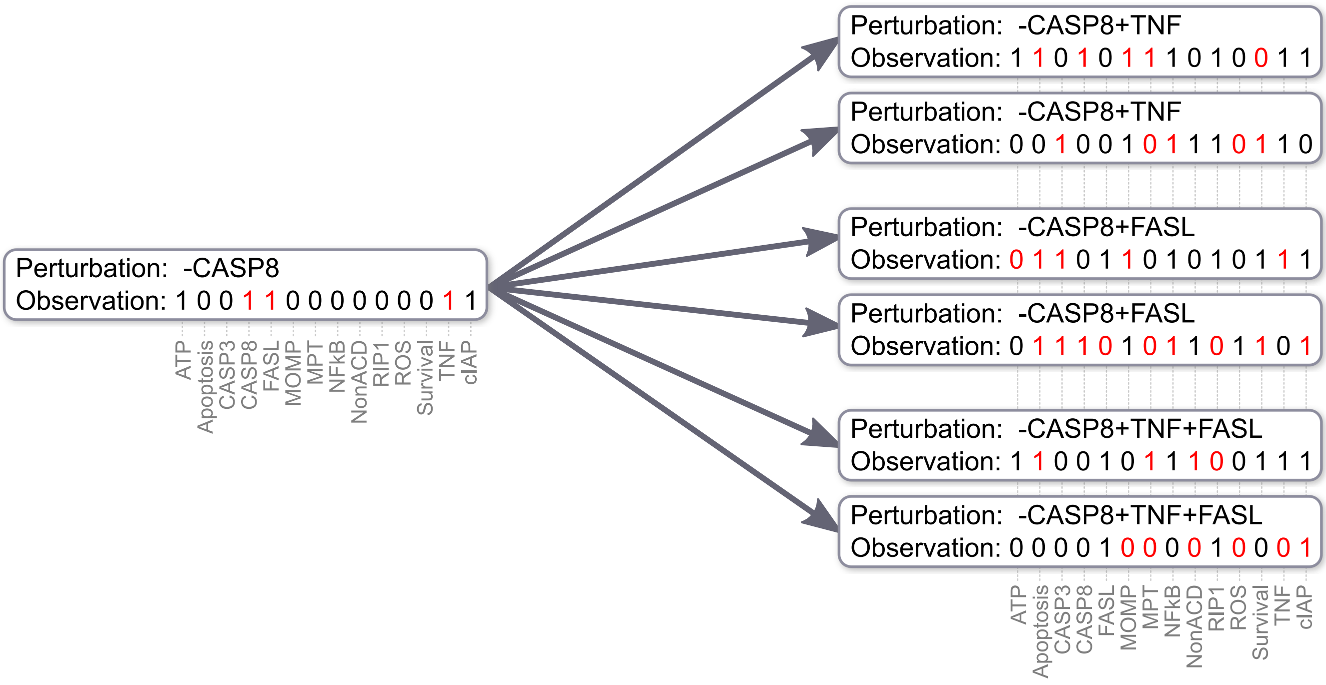
Perturbation: -CASP8
Observation: 1 0 0 1 1 0 0 0 0 0 0 0 1 1
ATP Apoptosis CASP3 CASP8 FASL MOMP MPT NFkB NonACD RIP1 ROS Survival TNF cIAP
Perturbation: -CASP8+TNF
Observation: 1 1 0 1 0 1 1 1 0 1 0 0 1 1
Perturbation: -CASP8+TNF
Observation: 0 0 1 0 0 1 0 1 1 1 0 1 1 0
Perturbation: -CASP8+FASL
Observation: 0 1 1 0 1 1 0 1 0 1 0 1 1 1
Perturbation: -CASP8+FASL
Observation: 0 1 1 1 0 1 0 1 1 0 1 1 0 1
Perturbation: -CASP8+TNF+FASL
Observation: 1 1 0 0 1 0 1 1 1 0 0 1 1 1
Perturbation: -CASP8+TNF+FASL
Observation: 0 0 0 0 1 0 0 0 0 1 0 0 0 1
ATP Apoptosis CASP3 CASP8 FASL MOMP MPT NFkB NonACD RIP1 ROS Survival TNF cIAP

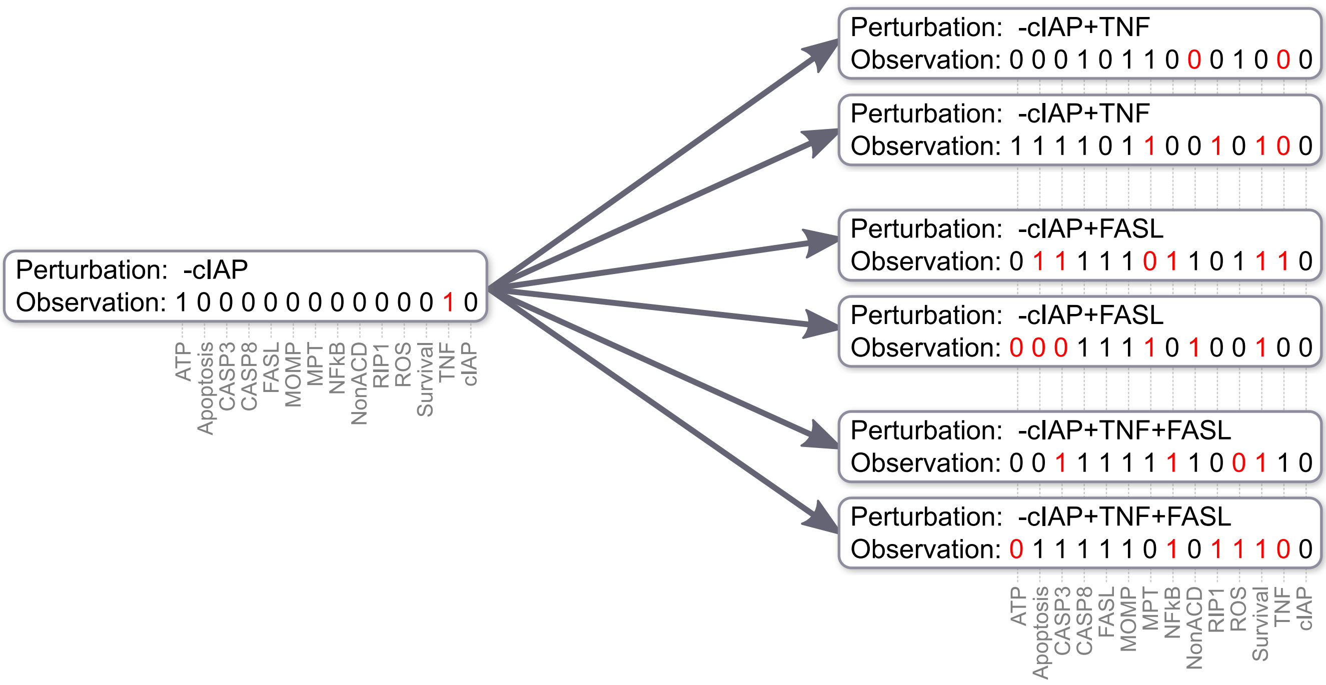
Perturbation: -cIAP
Observation: 1 0 0 0 0 0 0 0 0 0 0 0 1 0
ATP Apoptosis CASP3 CASP8 FASL MOMP MPT NFkB NonACD RIP1 ROS Survival TNF cIAP
Perturbation: -cIAP+TNF
Observation: 0 0 0 1 0 1 1 0 0 0 1 0 0 0
Perturbation: -cIAP+TNF
Observation: 1 1 1 1 0 1 1 0 0 1 0 1 0 0
Perturbation: -cIAP+FASL
Observation: 0 1 1 1 1 1 0 1 1 0 1 1 1 0
Perturbation: -cIAP+FASL
Observation: 0 0 0 1 1 1 1 0 1 0 0 1 0 0
Perturbation: -cIAP+TNF+FASL
Observation: 0 0 1 1 1 1 1 1 1 0 0 1 1 0
Perturbation: -cIAP+TNF+FASL
Observation: 0 1 1 1 1 1 0 1 0 1 1 1 0 0
ATP Apoptosis CASP3 CASP8 FASL MOMP MPT NFkB NonACD RIP1 ROS Survival TNF cIAP

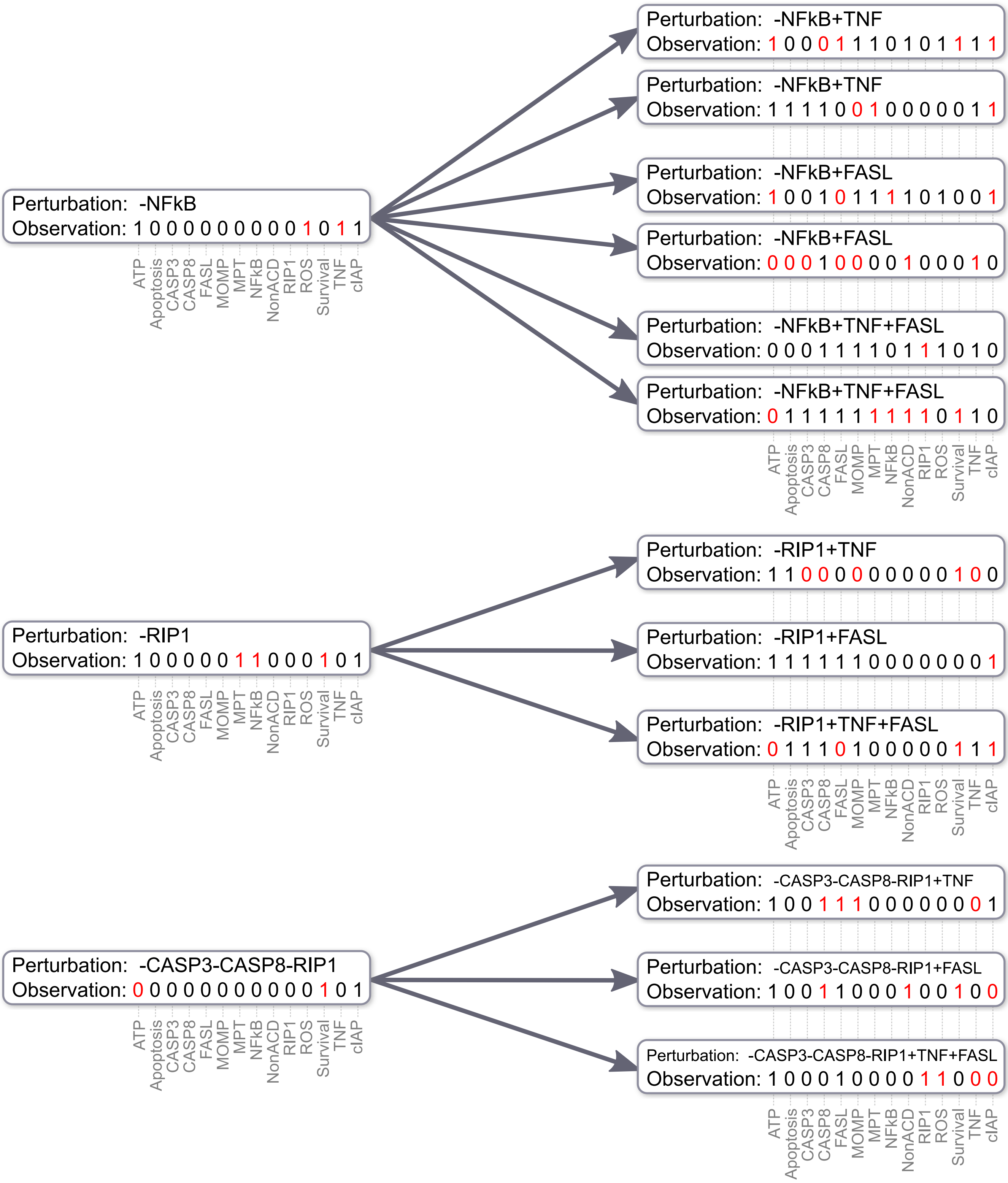
Perturbation: -NFkB
Observation: 1 0 0 0 0 0 0 0 0 0 1 0 1 1
Perturbation: -NFkB+TNF
Observation: 1 0 0 0 1 1 1 0 1 0 1 1 1 1
Perturbation: -NFkB+TNF
Observation: 1 1 1 1 0 0 1 0 0 0 0 0 1 1
Perturbation: -NFkB+FASL
Observation: 1 0 0 1 0 1 1 1 1 0 1 0 0 1
Perturbation: -NFkB+FASL
Observation: 0 0 0 1 0 0 0 0 1 0 0 0 1 0
Perturbation: -NFkB+TNF+FASL
Observation: 0 0 0 1 1 1 1 0 1 1 1 0 1 0
Perturbation: -NFkB+TNF+FASL
Observation: 0 1 1 1 1 1 1 1 1 1 0 1 1 0
Perturbation: -RIP1
Observation: 1 0 0 0 0 0 1 1 0 0 0 1 0 1
Perturbation: -RIP1+TNF
Observation: 1 1 0 0 0 0 0 0 0 0 0 1 0 0
Perturbation: -RIP1+FASL
Observation: 1 1 1 1 1 1 0 0 0 0 0 0 0 1
Perturbation: -RIP1+TNF+FASL
Observation: 0 1 1 1 0 1 0 0 0 0 0 1 1 1
Perturbation: -CASP3-CASP8-RIP1
Observation: 0 0 0 0 0 0 0 0 0 0 0 1 0 1
Perturbation: -CASP3-CASP8-RIP1+TNF
Observation: 1 0 0 1 1 1 0 0 0 0 0 0 0 1
Perturbation: -CASP3-CASP8-RIP1+FASL
Observation: 1 0 0 1 1 0 0 0 1 0 0 1 0 0
Perturbation: -CASP3-CASP8-RIP1+TNF+FASL
Observation: 1 0 0 0 1 0 0 0 0 1 1 0 0 0
ATP Apoptosis CASP3 CASP8 FASL MOMP MPT NFkB NonACD RIP1 ROS Survival TNF cIAP

Cell-fate decision model: training set 1 (Large) with 40% error

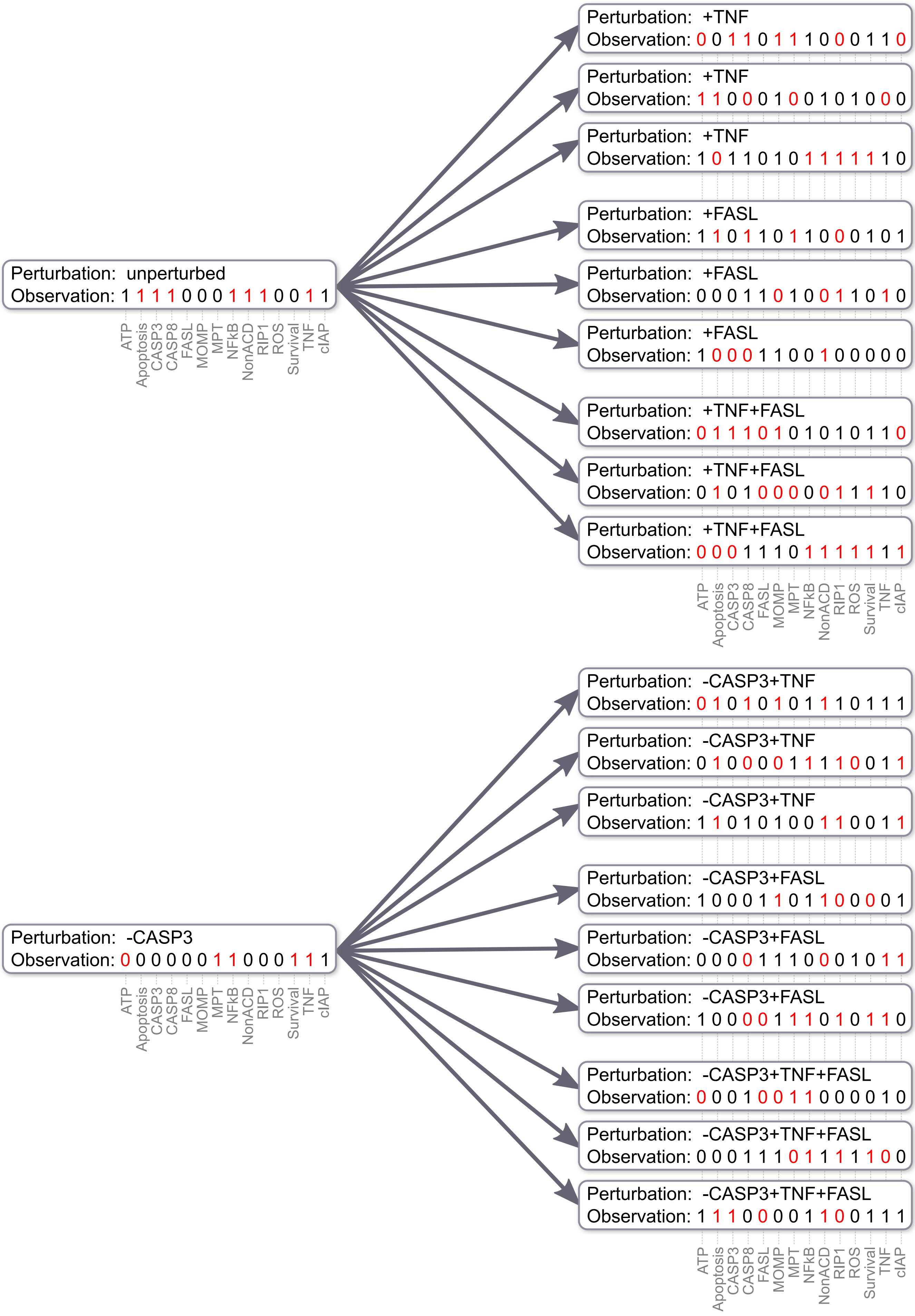

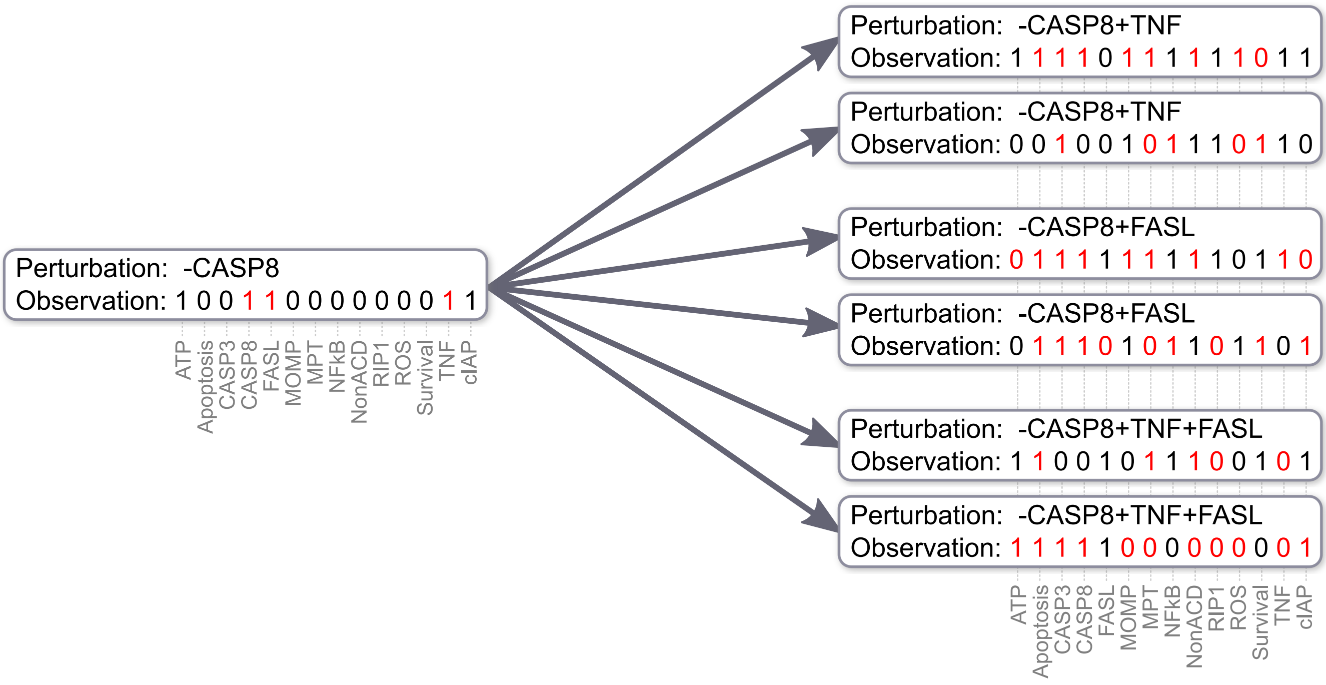
Perturbation: -CASP8
Observation: 1 0 0 1 1 0 0 0 0 0 0 0 1 1
ATP Apoptosis CASP3 CASP8 FASL MOMP MPT NFkB NonACD RIP1 ROS Survival TNF cIAP
Perturbation: -CASP8+TNF
Observation: 1 1 1 1 0 1 1 1 1 1 1 0 1 1
Perturbation: -CASP8+TNF
Observation: 0 0 1 0 0 1 0 1 1 1 0 1 1 0
Perturbation: -CASP8+FASL
Observation: 0 1 1 1 1 1 1 1 1 1 0 1 1 0
Perturbation: -CASP8+FASL
Observation: 0 1 1 1 0 1 0 1 1 0 1 1 0 1
Perturbation: -CASP8+TNF+FASL
Observation: 1 1 0 0 1 0 1 1 1 0 0 1 0 1
Perturbation: -CASP8+TNF+FASL
Observation: 1 1 1 1 1 0 0 0 0 0 0 0 0 1
ATP Apoptosis CASP3 CASP8 FASL MOMP MPT NFkB NonACD RIP1 ROS Survival TNF cIAP

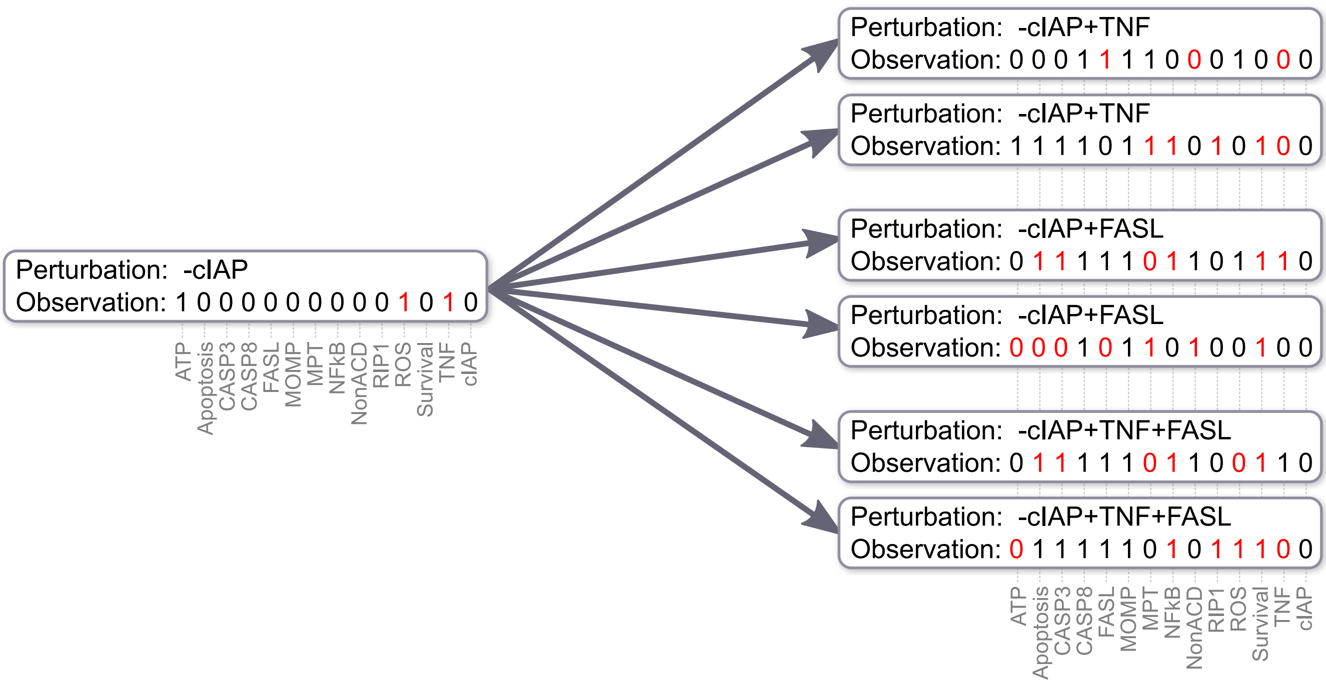
Perturbation: -cIAP
Observation: 1 0 0 0 0 0 0 0 0 0 1 0 1 0
ATP Apoptosis CASP3 CASP8 FASL MOMP MPT NFkB NonACD RIP1 ROS Survival TNF cIAP
Perturbation: -cIAP+TNF
Observation: 0 0 0 1 1 1 1 0 0 0 1 0 0 0
Perturbation: -cIAP+TNF
Observation: 1 1 1 1 0 1 1 1 0 1 0 1 0 0
Perturbation: -cIAP+FASL
Observation: 0 1 1 1 1 1 0 1 1 0 1 1 1 0
Perturbation: -cIAP+FASL
Observation: 0 0 0 1 0 1 1 0 1 0 0 1 0 0
Perturbation: -cIAP+TNF+FASL
Observation: 0 1 1 1 1 1 0 1 1 0 0 1 1 0
Perturbation: -cIAP+TNF+FASL
Observation: 0 1 1 1 1 1 0 1 0 1 1 1 0 0
ATP Apoptosis CASP3 CASP8 FASL MOMP MPT NFkB NonACD RIP1 ROS Survival TNF cIAP

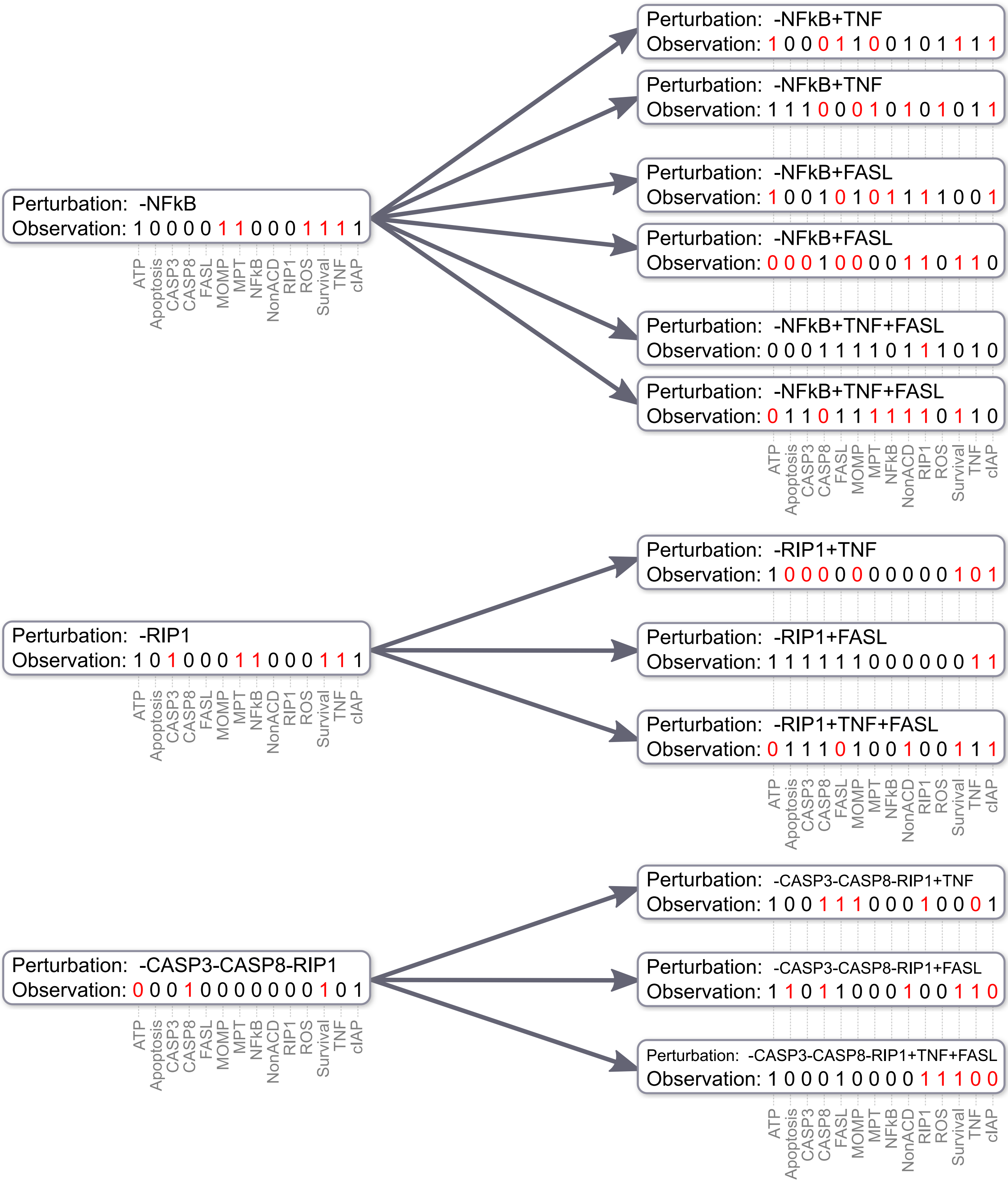
Perturbation: -NFkB
Observation: 1 0 0 0 0 1 1 0 0 0 1 1 1 1
Perturbation: -NFkB+TNF
Observation: 1 0 0 0 1 1 0 0 1 0 1 1 1 1
Perturbation: -NFkB+TNF
Observation: 1 1 1 0 0 0 1 0 1 0 1 0 1 1
Perturbation: -NFkB+FASL
Observation: 1 0 0 1 0 1 0 1 1 1 1 0 0 1
Perturbation: -NFkB+FASL
Observation: 0 0 0 1 0 0 0 0 1 1 0 1 1 0
Perturbation: -NFkB+TNF+FASL
Observation: 0 0 0 1 1 1 1 0 1 1 1 0 1 0
Perturbation: -NFkB+TNF+FASL
Observation: 0 1 1 0 1 1 1 1 1 1 0 1 1 0
Perturbation: -RIP1
Observation: 1 0 1 0 0 0 1 1 0 0 0 1 1 1
Perturbation: -RIP1+TNF
Observation: 1 0 0 0 0 0 0 0 0 0 0 1 0 1
Perturbation: -RIP1+FASL
Observation: 1 1 1 1 1 1 0 0 0 0 0 0 1 1
Perturbation: -RIP1+TNF+FASL
Observation: 0 1 1 1 0 1 0 0 1 0 0 1 1 1
Perturbation: -CASP3-CASP8-RIP1
Observation: 0 0 0 1 0 0 0 0 0 0 0 1 0 1
Perturbation: -CASP3-CASP8-RIP1+TNF
Observation: 1 0 0 1 1 1 0 0 0 1 0 0 0 1
Perturbation: -CASP3-CASP8-RIP1+FASL
Observation: 1 1 0 1 1 0 0 0 1 0 0 1 1 0
Perturbation: -CASP3-CASP8-RIP1+TNF+FASL
Observation: 1 0 0 0 1 0 0 0 0 1 1 1 0 0
ATP
Apoptosis
CASP3
CASP8
FASL
MOMP
MPT
NFkB
NonACD
RIP1
ROS
Survival
TNF
cIAP

Cell-fate decision model: training set 1 (Large) with 50% error

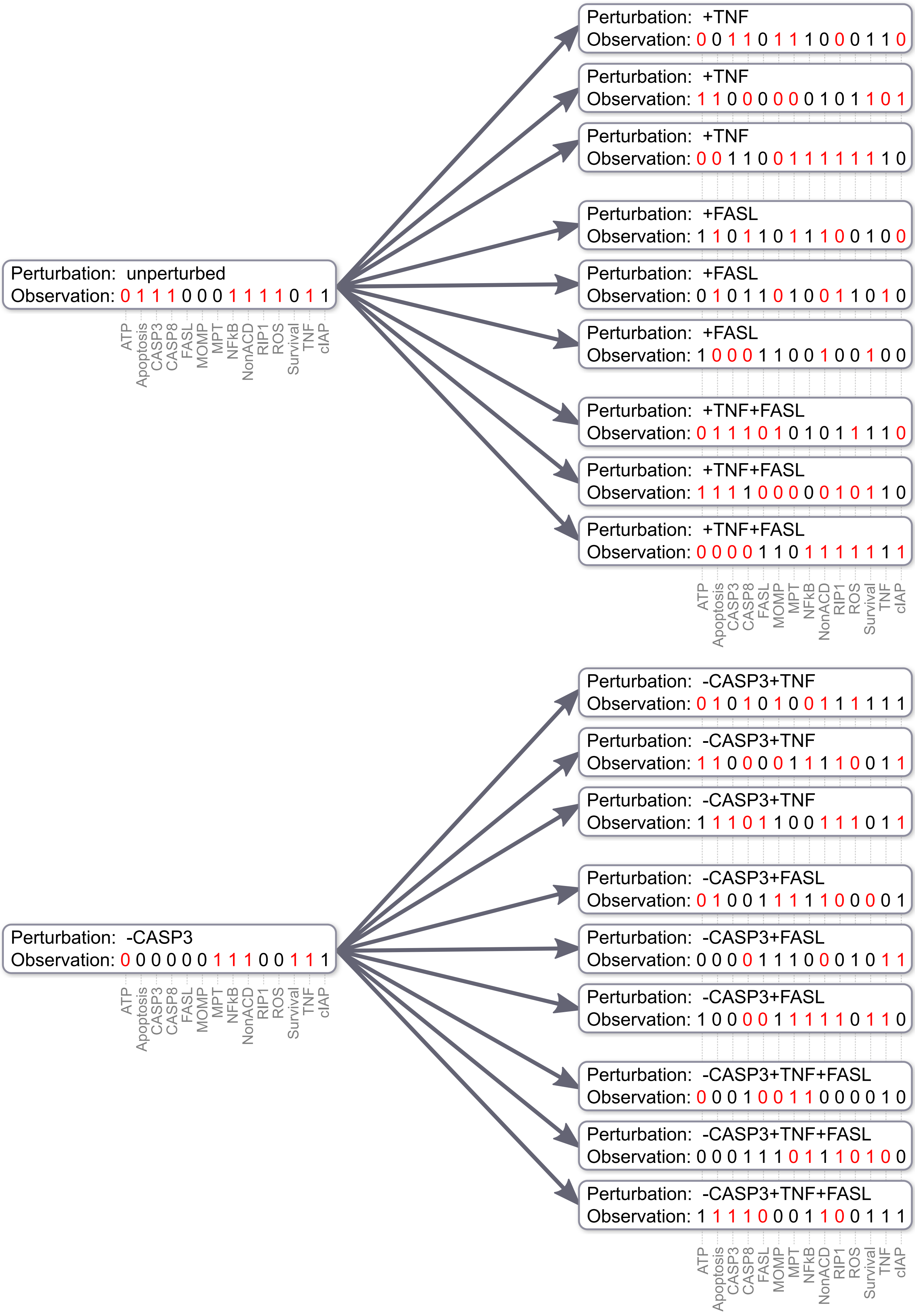

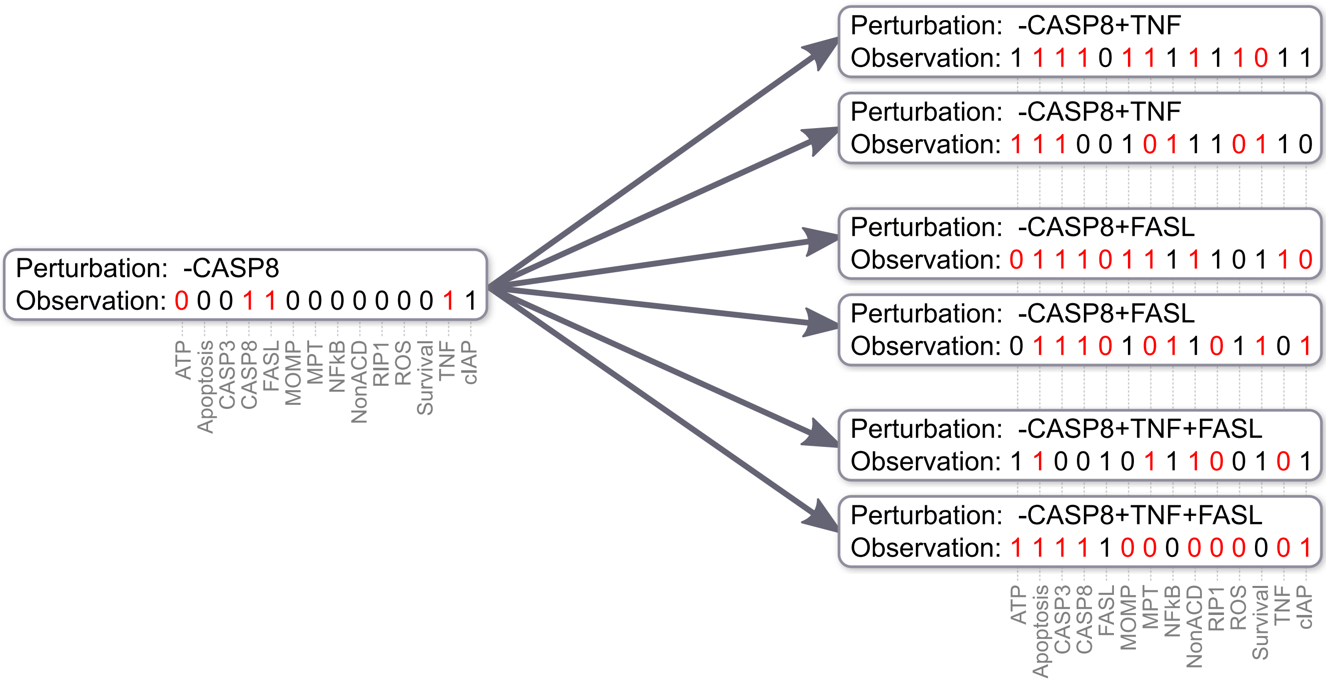
Perturbation: -CASP8
Observation: 0 0 0 1 1 0 0 0 0 0 0 0 1 1
ATP Apoptosis CASP3 CASP8 FASL MOMP MPT NFkB NonACD RIP1 ROS Survival TNF cIAP
Perturbation: -CASP8+TNF
Observation: 1 1 1 1 0 1 1 1 1 1 1 0 1 1
Perturbation: -CASP8+TNF
Observation: 1 1 1 0 0 1 0 1 1 1 0 1 1 0
Perturbation: -CASP8+FASL
Observation: 0 1 1 1 0 1 1 1 1 1 0 1 1 0
Perturbation: -CASP8+FASL
Observation: 0 1 1 1 0 1 0 1 1 0 1 1 0 1
Perturbation: -CASP8+TNF+FASL
Observation: 1 1 0 0 1 0 1 1 1 0 0 1 0 1
Perturbation: -CASP8+TNF+FASL
Observation: 1 1 1 1 1 0 0 0 0 0 0 0 0 1
ATP Apoptosis CASP3 CASP8 FASL MOMP MPT NFkB NonACD RIP1 ROS Survival TNF cIAP

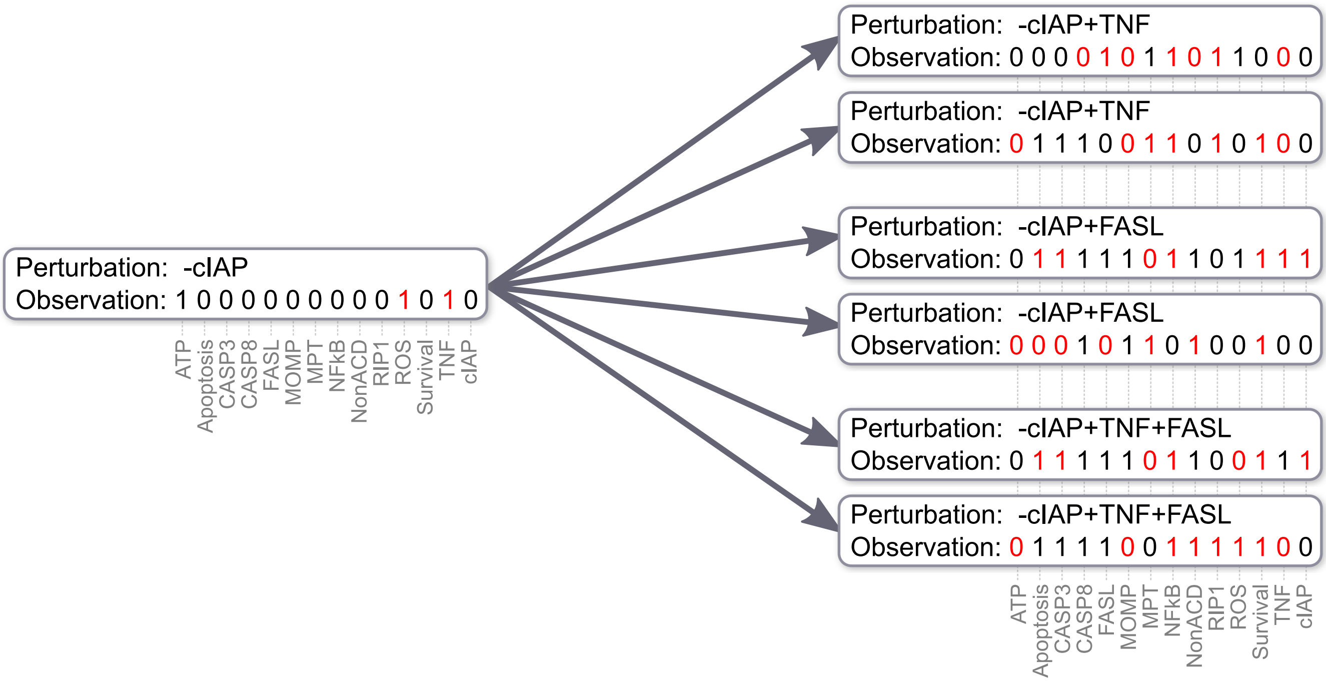
Perturbation: -cIAP
Observation: 1 0 0 0 0 0 0 0 0 0 1 0 1 0
ATP Apoptosis CASP3 CASP8 FASL MOMP MPT NFkB NonACD RIP1 ROS Survival TNF cIAP
Perturbation: -cIAP+TNF
Observation: 0 0 0 0 1 0 1 1 0 1 1 0 0 0
Perturbation: -cIAP+TNF
Observation: 0 1 1 1 0 0 1 1 0 1 0 1 0 0
Perturbation: -cIAP+FASL
Observation: 0 1 1 1 1 1 0 1 1 0 1 1 1 1
Perturbation: -cIAP+FASL
Observation: 0 0 0 1 0 1 1 0 1 0 0 1 0 0
Perturbation: -cIAP+TNF+FASL
Observation: 0 1 1 1 1 1 0 1 1 0 0 1 1 1
Perturbation: -cIAP+TNF+FASL
Observation: 0 1 1 1 1 0 0 1 1 1 1 1 0 0
ATP Apoptosis CASP3 CASP8 FASL MOMP MPT NFkB NonACD RIP1 ROS Survival TNF cIAP

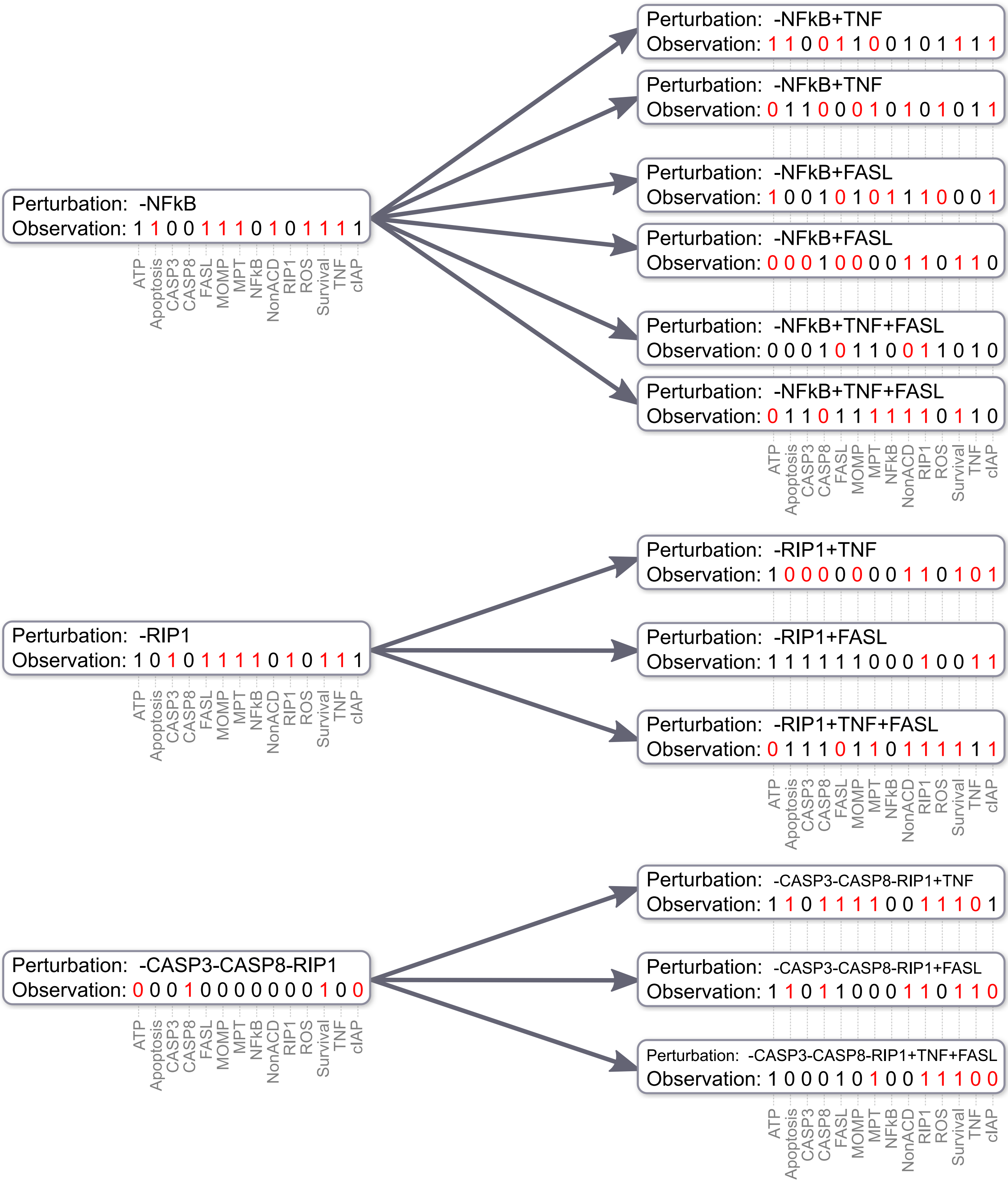
Perturbation: -NFkB
Observation: 1 1 0 0 1 1 1 0 1 0 1 1 1 1
Perturbation: -NFkB+TNF
Observation: 1 1 0 0 1 1 0 0 1 0 1 1 1 1
Perturbation: -NFkB+TNF
Observation: 0 1 1 0 0 0 1 0 1 0 1 0 1 1
Perturbation: -NFkB+FASL
Observation: 1 0 0 1 0 1 0 1 1 1 0 0 0 1
Perturbation: -NFkB+FASL
Observation: 0 0 0 1 0 0 0 0 1 1 0 1 1 0
Perturbation: -NFkB+TNF+FASL
Observation: 0 0 0 1 0 1 1 0 0 1 1 0 1 0
Perturbation: -NFkB+TNF+FASL
Observation: 0 1 1 0 1 1 1 1 1 1 0 1 1 0
Perturbation: -RIP1
Observation: 1 0 1 0 1 1 1 1 0 1 0 1 1 1
Perturbation: -RIP1+TNF
Observation: 1 0 0 0 0 0 0 0 1 1 0 1 0 1
Perturbation: -RIP1+FASL
Observation: 1 1 1 1 1 1 0 0 0 1 0 0 1 1
Perturbation: -RIP1+TNF+FASL
Observation: 0 1 1 1 0 1 1 0 1 1 1 1 1 1
Perturbation: -CASP3-CASP8-RIP1
Observation: 0 0 0 1 0 0 0 0 0 0 0 1 0 0
Perturbation: -CASP3-CASP8-RIP1+TNF
Observation: 1 1 0 1 1 1 1 0 0 1 1 1 0 1
Perturbation: -CASP3-CASP8-RIP1+FASL
Observation: 1 1 0 1 1 0 0 0 1 1 0 1 1 0
Perturbation: -CASP3-CASP8-RIP1+TNF+FASL
Observation: 1 0 0 0 1 0 1 0 0 1 1 1 0 0
ATP
Apoptosis
CASP3
CASP8
FASL
MOMP
MPT
NFkB
NonACD
RIP1
ROS
Survival
TNF
cIAP
