# Supplementary material for: Boolean regulatory network reconstruction using literature based knowledge with a genetic algorithm optimization method
Source: BMC Bioinformatics. 2016 Oct 6;17:410. doi: 10.1186/s12859-016-1287-z (PMC5053080; doi:10.1186/s12859-016-1287-z)
Supplement: Additional file 3: — Cell-fate decision model: single node perturbations. List of transitions between attractors from unperturbed to single node perturbations. Each line corresponds to one edge in the attractor reachability graph. Attractors with more than one state are replaced by the average over all states in the attractor. (PDF 63 kb) [file 12859_2016_1287_MOESM3_ESM.pdf]

Cell-fate decision model: in-silico experiments

Initial attractor																													Final attractor																											
Perturbation	ATP	Apoptosis	Apoptosome	BAX	BCL2	CASP3	CASP8	Cyt_c	DISC-FAS	DISC-TNF	FASL	IKK	MOMP	MPT	NFkB	NonACD	RIP1	RIP1K	RIP1ub	ROS	SMAC	Survival	TNF	TNFR	XIAP	cFLIP	cIAP		Perturbation	ATP	Apoptosis	Apoptosome	BAX	BCL2	CASP3	CASP8	Cyt_c	DISC-FAS	DISC-TNF	FASL	IKK	MOMP	MPT	NFkB	NonACD	RIP1	RIP1K	RIP1ub	ROS	SMAC	Survival	TNF	TNFR	XIAP	cFLIP	cIAP
Unperturbed	1	0	0	0	0	0	0	0	0	0	0	0	0	0	0	0	0	0	0	0	0	0	0	0	0	0	1	⟶	OE ATP	1	0	0	0	0	0	0	0	0	0	0	0	0	0	0	0	0	0	0	0	0	0	0	0	0	0	1
Unperturbed	1	0	0	0	0	0	0	0	0	0	0	0	0	0	0	0	0	0	0	0	0	0	0	0	0	0	1	⟶	OE Apoptosis	1	1	0	0	0	0	0	0	0	0	0	0	0	0	0	0	0	0	0	0	0	0	0	0	0	0	1
Unperturbed	1	0	0	0	0	0	0	0	0	0	0	0	0	0	0	0	0	0	0	0	0	0	0	0	0	0	1	⟶	OE Apoptosome	1	1	1	1	0	1	1	1	0	0	0	0	1	0	0	0	0	0	0	0	1	0	0	0	0	0	0
Unperturbed	1	0	0	0	0	0	0	0	0	0	0	0	0	0	0	0	0	0	0	0	0	0	0	0	0	0	1	⟶	OE BAX	1	1	1	1	0	1	1	1	0	0	0	0	1	0	0	0	0	0	0	0	1	0	0	0	0	0	0
Unperturbed	1	0	0	0	0	0	0	0	0	0	0	0	0	0	0	0	0	0	0	0	0	0	0	0	0	0	1	⟶	OE BCL2	1	0	0	0	1	0	0	0	0	0	0	0	0	0	0	0	0	0	0	0	0	0	0	0	0	0	1
Unperturbed	1	0	0	0	0	0	0	0	0	0	0	0	0	0	0	0	0	0	0	0	0	0	0	0	0	0	1	⟶	OE CASP3	1	1	1	1	0	1	1	1	0	0	0	0	1	0	0	0	0	0	0	0	1	0	0	0	0	0	0
Unperturbed	1	0	0	0	0	0	0	0	0	0	0	0	0	0	0	0	0	0	0	0	0	0	0	0	0	0	1	⟶	OE CASP8	1	1	1	1	0	1	1	1	0	0	0	0	1	0	0	0	0	0	0	0	1	0	0	0	0	0	0
Unperturbed	1	0	0	0	0	0	0	0	0	0	0	0	0	0	0	0	0	0	0	0	0	0	0	0	0	0	1	⟶	OE Cyt_c	1	1	1	1	0	1	1	1	0	0	0	0	1	0	0	0	0	0	0	0	1	0	0	0	0	0	0
Unperturbed	1	0	0	0	0	0	0	0	0	0	0	0	0	0	0	0	0	0	0	0	0	0	0	0	0	0	1	⟶	OE DISC-FAS	0	0	0	1	0	0	1	1	1	0	0	0	1	1	0	1	0	0	0	1	1	0	0	0	0	0	0
Unperturbed	1	0	0	0	0	0	0	0	0	0	0	0	0	0	0	0	0	0	0	0	0	0	0	0	0	0	1	⟶	OE DISC-FAS	1	0	0	0	1	0	0	0	1	0	0	1	0	0	1	0	1	1	1	0	0	1	0	0	1	1	1
Unperturbed	1	0	0	0	0	0	0	0	0	0	0	0	0	0	0	0	0	0	0	0	0	0	0	0	0	0	1	⟶	OE DISC-FAS	1	1	1	1	0	1	1	1	1	0	0	0	1	0	0	0	0	0	0	0	1	0	0	0	0	0	0
Unperturbed	1	0	0	0	0	0	0	0	0	0	0	0	0	0	0	0	0	0	0	0	0	0	0	0	0	0	1	⟶	OE DISC-TNF	1	1	1	1	0	1	1	1	0	1	0	0	1	0	0	0	0	0	0	0	1	0	0	0	0	0	0
Unperturbed	1	0	0	0	0	0	0	0	0	0	0	0	0	0	0	0	0	0	0	0	0	0	0	0	0	0	1	⟶	OE FASL	0	0	0	1	0	0	1	1	1	0	1	0	1	1	0	1	0	0	0	1	1	0	0	0	0	0	0
Unperturbed	1	0	0	0	0	0	0	0	0	0	0	0	0	0	0	0	0	0	0	0	0	0	0	0	0	0	1	⟶	OE FASL	1	0	0	0	1	0	0	0	1	0	1	1	0	0	1	0	1	1	1	0	0	1	0	0	1	1	1
Unperturbed	1	0	0	0	0	0	0	0	0	0	0	0	0	0	0	0	0	0	0	0	0	0	0	0	0	0	1	⟶	OE FASL	1	1	1	1	0	1	1	1	1	0	1	0	1	0	0	0	0	0	0	0	1	0	0	0	0	0	0
Unperturbed	1	0	0	0	0	0	0	0	0	0	0	0	0	0	0	0	0	0	0	0	0	0	0	0	0	0	1	⟶	OE IKK	1	0	0	0	1	0	0	0	0	0	0	1	0	0	1	0	0	0	0	0	0	1	0	0	1	1	1
Unperturbed	1	0	0	0	0	0	0	0	0	0	0	0	0	0	0	0	0	0	0	0	0	0	0	0	0	0	1	⟶	OE MOMP	1	1	1	1	0	1	1	1	0	0	0	0	1	0	0	0	0	0	0	0	1	0	0	0	0	0	0
Unperturbed	1	0	0	0	0	0	0	0	0	0	0	0	0	0	0	0	0	0	0	0	0	0	0	0	0	0	1	⟶	OE MPT	0	0	0	0	0	0	0	1	0	0	0	0	1	1	0	1	0	0	0	1	1	0	0	0	0	0	0
Unperturbed	1	0	0	0	0	0	0	0	0	0	0	0	0	0	0	0	0	0	0	0	0	0	0	0	0	0	1	⟶	OE NFkB	1	0	0	0	1	0	0	0	0	0	0	0	0	0	1	0	0	0	0	0	0	1	0	0	1	1	1
Unperturbed	1	0	0	0	0	0	0	0	0	0	0	0	0	0	0	0	0	0	0	0	0	0	0	0	0	0	1	⟶	OE NonACD	1	0	0	0	0	0	0	0	0	0	0	0	0	0	0	1	0	0	0	0	0	0	0	0	0	0	1
Unperturbed	1	0	0	0	0	0	0	0	0	0	0	0	0	0	0	0	0	0	0	0	0	0	0	0	0	0	1	⟶	OE RIP1	0	0	0	0	0	0	0	1	0	0	0	0	1	1	0	1	1	1	0	1	1	0	0	0	0	0	0
Unperturbed	1	0	0	0	0	0	0	0	0	0	0	0	0	0	0	0	0	0	0	0	0	0	0	0	0	0	1	⟶	OE RIP1	1	0	0	0	1	0	0	0	0	0	0	1	0	0	1	0	1	1	1	0	0	1	0	0	1	1	1
Unperturbed	1	0	0	0	0	0	0	0	0	0	0	0	0	0	0	0	0	0	0	0	0	0	0	0	0	0	1	⟶	OE RIP1K	0	0	0	0	0	0	0	1	0	0	0	0	1	1	0	1	0	1	0	1	1	0	0	0	0	0	0
Unperturbed	1	0	0	0	0	0	0	0	0	0	0	0	0	0	0	0	0	0	0	0	0	0	0	0	0	0	1	⟶	OE RIP1ub	1	0	0	0	1	0	0	0	0	0	0	1	0	0	1	0	0	0	1	0	0	1	0	0	1	1	1
Unperturbed	1	0	0	0	0	0	0	0	0	0	0	0	0	0	0	0	0	0	0	0	0	0	0	0	0	0	1	⟶	OE ROS	0	0	0	0	0	0	0	1	0	0	0	0	1	1	0	1	0	0	0	1	1	0	0	0	0	0	0
Unperturbed	1	0	0	0	0	0	0	0	0	0	0	0	0	0	0	0	0	0	0	0	0	0	0	0	0	0	1	⟶	OE SMAC	1	0	0	0	0	0	0	0	0	0	0	0	0	0	0	0	0	0	0	0	1	0	0	0	0	0	0
Unperturbed	1	0	0	0	0	0	0	0	0	0	0	0	0	0	0	0	0	0	0	0	0	0	0	0	0	0	1	⟶	OE Survival	1	0	0	0	0	0	0	0	0	0	0	0	0	0	0	0	0	0	0	0	0	1	0	0	0	0	1
Unperturbed	1	0	0	0	0	0	0	0	0	0	0	0	0	0	0	0	0	0	0	0	0	0	0	0	0	0	1	⟶	OE TNF	0	0	0	1	0	0	1	1	0	1	0	0	1	1	0	1	0	0	0	1	1	0	1	1	0	0	0
Unperturbed	1	0	0	0	0	0	0	0	0	0	0	0	0	0	0	0	0	0	0	0	0	0	0	0	0	0	1	⟶	OE TNF	1	0	0	0	1	0	0	0	0	1	0	1	0	0	1	0	1	1	1	0	0	1	1	1	1	1	1
Unperturbed	1	0	0	0	0	0	0	0	0	0	0	0	0	0	0	0	0	0	0	0	0	0	0	0	0	0	1	⟶	OE TNF	1	1	1	1	0	1	1	1	0	1	0	0	1	0	0	0	0	0	0	0	1	0	1	1	0	0	0
Unperturbed	1	0	0	0	0	0	0	0	0	0	0	0	0	0	0	0	0	0	0	0	0	0	0	0	0	0	1	⟶	OE TNFR	0	0	0	1	0	0	1	1	0	1	0	0	1	1	0	1	0	0	0	1	1	0	0	1	0	0	0
Unperturbed	1	0	0	0	0	0	0	0	0	0	0	0	0	0	0	0	0	0	0	0	0	0	0	0	0	0	1	⟶	OE TNFR	1	0	0	0	1	0	0	0	0	1	0	1	0	0	1	0	1	1	1	0	0	1	0	1	1	1	1
Unperturbed	1	0	0	0	0	0	0	0	0	0	0	0	0	0	0	0	0	0	0	0	0	0	0	0	0	0	1	⟶	OE TNFR	1	1	1	1	0	1	1	1	0	1	0	0	1	0	0	0	0	0	0	0	1	0	0	1	0	0	0
Unperturbed	1	0	0	0	0	0	0	0	0	0	0	0	0	0	0	0	0	0	0	0	0	0	0	0	0	0	1	⟶	OE XIAP	1	0	0	0	0	0	0	0	0	0	0	0	0	0	0	0	0	0	0	0	0	0	0	0	1	0	1
Unperturbed	1	0	0	0	0	0	0	0	0	0	0	0	0	0	0	0	0	0	0	0	0	0	0	0	0	0	1	⟶	OE cFLIP	1	0	0	0	0	0	0	0	0	0	0	0	0	0	0	0	0	0	0	0	0	0	0	0	0	1	1
Unperturbed	1	0	0	0	0	0	0	0	0	0	0	0	0	0	0	0	0	0	0	0	0	0	0	0	0	0	1	⟶	OE cIAP	1	0	0	0	0	0	0	0	0	0	0	0	0	0	0	0	0	0	0	0	0	0	0	0	0	0	1
Unperturbed	1	0	0	0	0	0	0	0	0	0	0	0	0	0	0	0	0	0	0	0	0	0	0	0	0	0	1	⟶	KO ATP	0	0	0	0	0	0	0	0	0	0	0	0	0	0	0	1	0	0	0	0	0	0	0	0	0	0	1
Unperturbed	1	0	0	0	0	0	0	0	0	0	0	0	0	0	0	0	0	0	0	0	0	0	0	0	0	0	1	⟶	KO Apoptosis	1	0	0	0	0	0	0	0	0	0	0	0	0	0	0	0	0	0	0	0	0	0	0	0	0	0	1
Unperturbed	1	0	0	0	0	0	0	0	0	0	0	0	0	0	0	0	0	0	0	0	0	0	0	0	0	0	1	⟶	KO Apoptosome	1	0	0	0	0	0	0	0	0	0	0	0	0	0	0	0	0	0	0	0	0	0	0	0	0	0	1
Unperturbed	1	0	0	0	0	0	0	0	0	0	0	0	0	0	0	0	0	0	0	0	0	0	0	0	0	0	1	⟶	KO BAX	1	0	0	0	0	0	0	0	0	0	0	0	0	0	0	0	0	0	0	0	0	0	0	0	0	0	1
Unperturbed	1	0	0	0	0	0	0	0	0	0	0	0	0	0	0	0	0	0	0	0	0	0	0	0	0	0	1	⟶	KO BCL2	1	0	0	0	0	0	0	0	0	0	0	0	0	0	0	0	0	0	0	0	0	0	0	0	0	0	1
Unperturbed	1	0	0	0	0	0	0	0	0	0	0	0	0	0	0	0	0	0	0	0	0	0	0	0	0	0	1	⟶	KO CASP3	1	0	0	0	0	0	0	0	0	0	0	0	0	0	0	0	0	0	0	0	0	0	0	0	0	0	1
Unperturbed	1	0	0	0	0	0	0	0	0	0	0	0	0	0	0	0	0	0	0	0	0	0	0	0	0	0	1	⟶	KO CASP8	1	0	0	0	0	0	0	0	0	0	0	0	0	0	0	0	0	0	0	0	0	0	0	0	0	0	1

Initial attractor																													Final attractor																											
Perturbation	ATP	Apoptosis	Apoptosome	BAX	BCL2	CASP3	CASP8	Cyt_c	DISC-FAS	DISC-TNF	FASL	IKK	MOMP	MPT	NFkB	NonACD	RIP1	RIP1K	RIP1ub	ROS	SMAC	Survival	TNF	TNFR	XIAP	cFLIP	cIAP		Perturbation	ATP	Apoptosis	Apoptosome	BAX	BCL2	CASP3	CASP8	Cyt_c	DISC-FAS	DISC-TNF	FASL	IKK	MOMP	MPT	NFkB	NonACD	RIP1	RIP1K	RIP1ub	ROS	SMAC	Survival	TNF	TNFR	XIAP	cFLIP	cIAP
Unperturbed	1	0	0	0	0	0	0	0	0	0	0	0	0	0	0	0	0	0	0	0	0	0	0	0	0	0	1	⟶	KO Cyt_c	1	0	0	0	0	0	0	0	0	0	0	0	0	0	0	0	0	0	0	0	0	0	0	0	0	0	1
Unperturbed	1	0	0	0	0	0	0	0	0	0	0	0	0	0	0	0	0	0	0	0	0	0	0	0	0	0	1	⟶	KO DISC-FAS	1	0	0	0	0	0	0	0	0	0	0	0	0	0	0	0	0	0	0	0	0	0	0	0	0	0	1
Unperturbed	1	0	0	0	0	0	0	0	0	0	0	0	0	0	0	0	0	0	0	0	0	0	0	0	0	0	1	⟶	KO DISC-TNF	1	0	0	0	0	0	0	0	0	0	0	0	0	0	0	0	0	0	0	0	0	0	0	0	0	0	1
Unperturbed	1	0	0	0	0	0	0	0	0	0	0	0	0	0	0	0	0	0	0	0	0	0	0	0	0	0	1	⟶	KO FASL	1	0	0	0	0	0	0	0	0	0	0	0	0	0	0	0	0	0	0	0	0	0	0	0	0	0	1
Unperturbed	1	0	0	0	0	0	0	0	0	0	0	0	0	0	0	0	0	0	0	0	0	0	0	0	0	0	1	⟶	KO IKK	1	0	0	0	0	0	0	0	0	0	0	0	0	0	0	0	0	0	0	0	0	0	0	0	0	0	1
Unperturbed	1	0	0	0	0	0	0	0	0	0	0	0	0	0	0	0	0	0	0	0	0	0	0	0	0	0	1	⟶	KO MOMP	1	0	0	0	0	0	0	0	0	0	0	0	0	0	0	0	0	0	0	0	0	0	0	0	0	0	1
Unperturbed	1	0	0	0	0	0	0	0	0	0	0	0	0	0	0	0	0	0	0	0	0	0	0	0	0	0	1	⟶	KO MPT	1	0	0	0	0	0	0	0	0	0	0	0	0	0	0	0	0	0	0	0	0	0	0	0	0	0	1
Unperturbed	1	0	0	0	0	0	0	0	0	0	0	0	0	0	0	0	0	0	0	0	0	0	0	0	0	0	1	⟶	KO NFkB	1	0	0	0	0	0	0	0	0	0	0	0	0	0	0	0	0	0	0	0	0	0	0	0	0	0	1
Unperturbed	1	0	0	0	0	0	0	0	0	0	0	0	0	0	0	0	0	0	0	0	0	0	0	0	0	0	1	⟶	KO NonACD	1	0	0	0	0	0	0	0	0	0	0	0	0	0	0	0	0	0	0	0	0	0	0	0	0	0	1
Unperturbed	1	0	0	0	0	0	0	0	0	0	0	0	0	0	0	0	0	0	0	0	0	0	0	0	0	0	1	⟶	KO RIP1	1	0	0	0	0	0	0	0	0	0	0	0	0	0	0	0	0	0	0	0	0	0	0	0	0	0	1
Unperturbed	1	0	0	0	0	0	0	0	0	0	0	0	0	0	0	0	0	0	0	0	0	0	0	0	0	0	1	⟶	KO RIP1K	1	0	0	0	0	0	0	0	0	0	0	0	0	0	0	0	0	0	0	0	0	0	0	0	0	0	1
Unperturbed	1	0	0	0	0	0	0	0	0	0	0	0	0	0	0	0	0	0	0	0	0	0	0	0	0	0	1	⟶	KO RIP1ub	1	0	0	0	0	0	0	0	0	0	0	0	0	0	0	0	0	0	0	0	0	0	0	0	0	0	1
Unperturbed	1	0	0	0	0	0	0	0	0	0	0	0	0	0	0	0	0	0	0	0	0	0	0	0	0	0	1	⟶	KO ROS	1	0	0	0	0	0	0	0	0	0	0	0	0	0	0	0	0	0	0	0	0	0	0	0	0	0	1
Unperturbed	1	0	0	0	0	0	0	0	0	0	0	0	0	0	0	0	0	0	0	0	0	0	0	0	0	0	1	⟶	KO SMAC	1	0	0	0	0	0	0	0	0	0	0	0	0	0	0	0	0	0	0	0	0	0	0	0	0	0	1
Unperturbed	1	0	0	0	0	0	0	0	0	0	0	0	0	0	0	0	0	0	0	0	0	0	0	0	0	0	1	⟶	KO Survival	1	0	0	0	0	0	0	0	0	0	0	0	0	0	0	0	0	0	0	0	0	0	0	0	0	0	1
Unperturbed	1	0	0	0	0	0	0	0	0	0	0	0	0	0	0	0	0	0	0	0	0	0	0	0	0	0	1	⟶	KO TNF	1	0	0	0	0	0	0	0	0	0	0	0	0	0	0	0	0	0	0	0	0	0	0	0	0	0	1
Unperturbed	1	0	0	0	0	0	0	0	0	0	0	0	0	0	0	0	0	0	0	0	0	0	0	0	0	0	1	⟶	KO TNFR	1	0	0	0	0	0	0	0	0	0	0	0	0	0	0	0	0	0	0	0	0	0	0	0	0	0	1
Unperturbed	1	0	0	0	0	0	0	0	0	0	0	0	0	0	0	0	0	0	0	0	0	0	0	0	0	0	1	⟶	KO XIAP	1	0	0	0	0	0	0	0	0	0	0	0	0	0	0	0	0	0	0	0	0	0	0	0	0	0	1
Unperturbed	1	0	0	0	0	0	0	0	0	0	0	0	0	0	0	0	0	0	0	0	0	0	0	0	0	0	1	⟶	KO cFLIP	1	0	0	0	0	0	0	0	0	0	0	0	0	0	0	0	0	0	0	0	0	0	0	0	0	0	1
Unperturbed	1	0	0	0	0	0	0	0	0	0	0	0	0	0	0	0	0	0	0	0	0	0	0	0	0	0	1	⟶	KO cIAP	1	0	0	0	0	0	0	0	0	0	0	0	0	0	0	0	0	0	0	0	0	0	0	0	0	0	0
Unperturbed	1	0	0	0	0	0	0	0	0	0	0	0	0	0	0	0	0	0	0	0	0	0	0	0	0	0	0	⟶	OE ATP	1	0	0	0	0	0	0	0	0	0	0	0	0	0	0	0	0	0	0	0	0	0	0	0	0	0	0
Unperturbed	1	0	0	0	0	0	0	0	0	0	0	0	0	0	0	0	0	0	0	0	0	0	0	0	0	0	0	⟶	OE Apoptosis	1	1	0	0	0	0	0	0	0	0	0	0	0	0	0	0	0	0	0	0	0	0	0	0	0	0	0
Unperturbed	1	0	0	0	0	0	0	0	0	0	0	0	0	0	0	0	0	0	0	0	0	0	0	0	0	0	0	⟶	OE Apoptosome	1	1	1	1	0	1	1	1	0	0	0	0	1	0	0	0	0	0	0	0	1	0	0	0	0	0	0
Unperturbed	1	0	0	0	0	0	0	0	0	0	0	0	0	0	0	0	0	0	0	0	0	0	0	0	0	0	0	⟶	OE BAX	1	1	1	1	0	1	1	1	0	0	0	0	1	0	0	0	0	0	0	0	1	0	0	0	0	0	0
Unperturbed	1	0	0	0	0	0	0	0	0	0	0	0	0	0	0	0	0	0	0	0	0	0	0	0	0	0	0	⟶	OE BCL2	1	0	0	0	1	0	0	0	0	0	0	0	0	0	0	0	0	0	0	0	0	0	0	0	0	0	0
Unperturbed	1	0	0	0	0	0	0	0	0	0	0	0	0	0	0	0	0	0	0	0	0	0	0	0	0	0	0	⟶	OE CASP3	1	1	1	1	0	1	1	1	0	0	0	0	1	0	0	0	0	0	0	0	1	0	0	0	0	0	0
Unperturbed	1	0	0	0	0	0	0	0	0	0	0	0	0	0	0	0	0	0	0	0	0	0	0	0	0	0	0	⟶	OE CASP8	1	1	1	1	0	1	1	1	0	0	0	0	1	0	0	0	0	0	0	0	1	0	0	0	0	0	0
Unperturbed	1	0	0	0	0	0	0	0	0	0	0	0	0	0	0	0	0	0	0	0	0	0	0	0	0	0	0	⟶	OE Cyt_c	1	1	1	1	0	1	1	1	0	0	0	0	1	0	0	0	0	0	0	0	1	0	0	0	0	0	0
Unperturbed	1	0	0	0	0	0	0	0	0	0	0	0	0	0	0	0	0	0	0	0	0	0	0	0	0	0	0	⟶	OE DISC-FAS	0	0	0	1	0	0	1	1	1	0	0	0	1	1	0	1	0	0	0	1	1	0	0	0	0	0	0
Unperturbed	1	0	0	0	0	0	0	0	0	0	0	0	0	0	0	0	0	0	0	0	0	0	0	0	0	0	0	⟶	OE DISC-FAS	1	1	1	1	0	1	1	1	1	0	0	0	1	0	0	0	0	0	0	0	1	0	0	0	0	0	0
Unperturbed	1	0	0	0	0	0	0	0	0	0	0	0	0	0	0	0	0	0	0	0	0	0	0	0	0	0	0	⟶	OE DISC-TNF	1	1	1	1	0	1	1	1	0	1	0	0	1	0	0	0	0	0	0	0	1	0	0	0	0	0	0
Unperturbed	1	0	0	0	0	0	0	0	0	0	0	0	0	0	0	0	0	0	0	0	0	0	0	0	0	0	0	⟶	OE FASL	0	0	0	1	0	0	1	1	1	0	1	0	1	1	0	1	0	0	0	1	1	0	0	0	0	0	0
Unperturbed	1	0	0	0	0	0	0	0	0	0	0	0	0	0	0	0	0	0	0	0	0	0	0	0	0	0	0	⟶	OE FASL	1	1	1	1	0	1	1	1	1	0	1	0	1	0	0	0	0	0	0	0	1	0	0	0	0	0	0
Unperturbed	1	0	0	0	0	0	0	0	0	0	0	0	0	0	0	0	0	0	0	0	0	0	0	0	0	0	0	⟶	OE IKK	1	0	0	0	1	0	0	0	0	0	0	1	0	0	1	0	0	0	0	0	0	1	0	0	1	1	1
Unperturbed	1	0	0	0	0	0	0	0	0	0	0	0	0	0	0	0	0	0	0	0	0	0	0	0	0	0	0	⟶	OE MOMP	1	1	1	1	0	1	1	1	0	0	0	0	1	0	0	0	0	0	0	0	1	0	0	0	0	0	0
Unperturbed	1	0	0	0	0	0	0	0	0	0	0	0	0	0	0	0	0	0	0	0	0	0	0	0	0	0	0	⟶	OE MPT	0	0	0	0	0	0	0	1	0	0	0	0	1	1	0	1	0	0	0	1	1	0	0	0	0	0	0
Unperturbed	1	0	0	0	0	0	0	0	0	0	0	0	0	0	0	0	0	0	0	0	0	0	0	0	0	0	0	⟶	OE NFkB	1	0	0	0	1	0	0	0	0	0	0	0	0	0	1	0	0	0	0	0	0	1	0	0	1	1	1
Unperturbed	1	0	0	0	0	0	0	0	0	0	0	0	0	0	0	0	0	0	0	0	0	0	0	0	0	0	0	⟶	OE NonACD	1	0	0	0	0	0	0	0	0	0	0	0	0	0	0	1	0	0	0	0	0	0	0	0	0	0	0
Unperturbed	1	0	0	0	0	0	0	0	0	0	0	0	0	0	0	0	0	0	0	0	0	0	0	0	0	0	0	⟶	OE RIP1	0	0	0	0	0	0	0	1	0	0	0	0	1	1	0	1	1	1	0	1	1	0	0	0	0	0	0
Unperturbed	1	0	0	0	0	0	0	0	0	0	0	0	0	0	0	0	0	0	0	0	0	0	0	0	0	0	0	⟶	OE RIP1K	0	0	0	0	0	0	0	1	0	0	0	0	1	1	0	1	0	1	0	1	1	0	0	0	0	0	0
Unperturbed	1	0	0	0	0	0	0	0	0	0	0	0	0	0	0	0	0	0	0	0	0	0	0	0	0	0	0	⟶	OE RIP1ub	1	0	0	0	1	0	0	0	0	0	0	1	0	0	1	0	0	0	1	0	0	1	0	0	1	1	1
Unperturbed	1	0	0	0	0	0	0	0	0	0	0	0	0	0	0	0	0	0	0	0	0	0	0	0	0	0	0	⟶	OE ROS	0	0	0	0	0	0	0	1	0	0	0	0	1	1	0	1	0	0	0	1	1	0	0	0	0	0	0
Unperturbed	1	0	0	0	0	0	0	0	0	0	0	0	0	0	0	0	0	0	0	0	0	0	0	0	0	0	0	⟶	OE SMAC	1	0	0	0	0	0	0	0	0	0	0	0	0	0	0	0	0	0	0	0	1	0	0	0	0	0	0
Unperturbed	1	0	0	0	0	0	0	0	0	0	0	0	0	0	0	0	0	0	0	0	0	0	0	0	0	0	0	⟶	OE Survival	1	0	0	0	0	0	0	0	0	0	0	0	0	0	0	0	0	0	0	0	0	1	0	0	0	0	0
Unperturbed	1	0	0	0	0	0	0	0	0	0	0	0	0	0	0	0	0	0	0	0	0	0	0	0	0	0	0	⟶	OE TNF	0	0	0	1	0	0	1	1	0	1	0	0	1	1	0	1	0	0	0	1	1	0	1	1	0	0	0
Unperturbed	1	0	0	0	0	0	0	0	0	0	0	0	0	0	0	0	0	0	0	0	0	0	0	0	0	0	0	⟶	OE TNF	1	1	1	1	0	1	1	1	0	1	0	0	1	0	0	0	0	0	0	0	1	0	1	1	0	0	0

Initial attractor																													Final attractor																											
Perturbation	ATP	Apoptosis	Apoptosome	BAX	BCL2	CASP3	CASP8	Cyt_c	DISC-FAS	DISC-TNF	FASL	IKK	MOMP	MPT	NFkB	NonACD	RIP1	RIP1K	RIP1ub	ROS	SMAC	Survival	TNF	TNFR	XIAP	cFLIP	cIAP		Perturbation	ATP	Apoptosis	Apoptosome	BAX	BCL2	CASP3	CASP8	Cyt_c	DISC-FAS	DISC-TNF	FASL	IKK	MOMP	MPT	NFkB	NonACD	RIP1	RIP1K	RIP1ub	ROS	SMAC	Survival	TNF	TNFR	XIAP	cFLIP	cIAP
Unperturbed	1	0	0	0	0	0	0	0	0	0	0	0	0	0	0	0	0	0	0	0	0	0	0	0	0	0	0	⟶	OE TNFR	0	0	0	1	0	0	1	1	0	1	0	0	1	1	0	1	0	0	0	1	1	0	0	1	0	0	0
Unperturbed	1	0	0	0	0	0	0	0	0	0	0	0	0	0	0	0	0	0	0	0	0	0	0	0	0	0	0	⟶	OE TNFR	1	1	1	1	0	1	1	1	0	1	0	0	1	0	0	0	0	0	0	0	1	0	0	1	0	0	0
Unperturbed	1	0	0	0	0	0	0	0	0	0	0	0	0	0	0	0	0	0	0	0	0	0	0	0	0	0	0	⟶	OE XIAP	1	0	0	0	0	0	0	0	0	0	0	0	0	0	0	0	0	0	0	0	0	0	0	0	1	0	0
Unperturbed	1	0	0	0	0	0	0	0	0	0	0	0	0	0	0	0	0	0	0	0	0	0	0	0	0	0	0	⟶	OE cFLIP	1	0	0	0	0	0	0	0	0	0	0	0	0	0	0	0	0	0	0	0	0	0	0	0	0	1	0
Unperturbed	1	0	0	0	0	0	0	0	0	0	0	0	0	0	0	0	0	0	0	0	0	0	0	0	0	0	0	⟶	OE cIAP	1	0	0	0	0	0	0	0	0	0	0	0	0	0	0	0	0	0	0	0	0	0	0	0	0	0	1
Unperturbed	1	0	0	0	0	0	0	0	0	0	0	0	0	0	0	0	0	0	0	0	0	0	0	0	0	0	0	⟶	KO ATP	0	0	0	0	0	0	0	0	0	0	0	0	0	0	0	1	0	0	0	0	0	0	0	0	0	0	0
Unperturbed	1	0	0	0	0	0	0	0	0	0	0	0	0	0	0	0	0	0	0	0	0	0	0	0	0	0	0	⟶	KO Apoptosis	1	0	0	0	0	0	0	0	0	0	0	0	0	0	0	0	0	0	0	0	0	0	0	0	0	0	0
Unperturbed	1	0	0	0	0	0	0	0	0	0	0	0	0	0	0	0	0	0	0	0	0	0	0	0	0	0	0	⟶	KO Apoptosome	1	0	0	0	0	0	0	0	0	0	0	0	0	0	0	0	0	0	0	0	0	0	0	0	0	0	0
Unperturbed	1	0	0	0	0	0	0	0	0	0	0	0	0	0	0	0	0	0	0	0	0	0	0	0	0	0	0	⟶	KO BAX	1	0	0	0	0	0	0	0	0	0	0	0	0	0	0	0	0	0	0	0	0	0	0	0	0	0	0
Unperturbed	1	0	0	0	0	0	0	0	0	0	0	0	0	0	0	0	0	0	0	0	0	0	0	0	0	0	0	⟶	KO BCL2	1	0	0	0	0	0	0	0	0	0	0	0	0	0	0	0	0	0	0	0	0	0	0	0	0	0	0
Unperturbed	1	0	0	0	0	0	0	0	0	0	0	0	0	0	0	0	0	0	0	0	0	0	0	0	0	0	0	⟶	KO CASP3	1	0	0	0	0	0	0	0	0	0	0	0	0	0	0	0	0	0	0	0	0	0	0	0	0	0	0
Unperturbed	1	0	0	0	0	0	0	0	0	0	0	0	0	0	0	0	0	0	0	0	0	0	0	0	0	0	0	⟶	KO CASP8	1	0	0	0	0	0	0	0	0	0	0	0	0	0	0	0	0	0	0	0	0	0	0	0	0	0	0
Unperturbed	1	0	0	0	0	0	0	0	0	0	0	0	0	0	0	0	0	0	0	0	0	0	0	0	0	0	0	⟶	KO Cyt_c	1	0	0	0	0	0	0	0	0	0	0	0	0	0	0	0	0	0	0	0	0	0	0	0	0	0	0
Unperturbed	1	0	0	0	0	0	0	0	0	0	0	0	0	0	0	0	0	0	0	0	0	0	0	0	0	0	0	⟶	KO DISC-FAS	1	0	0	0	0	0	0	0	0	0	0	0	0	0	0	0	0	0	0	0	0	0	0	0	0	0	0
Unperturbed	1	0	0	0	0	0	0	0	0	0	0	0	0	0	0	0	0	0	0	0	0	0	0	0	0	0	0	⟶	KO DISC-TNF	1	0	0	0	0	0	0	0	0	0	0	0	0	0	0	0	0	0	0	0	0	0	0	0	0	0	0
Unperturbed	1	0	0	0	0	0	0	0	0	0	0	0	0	0	0	0	0	0	0	0	0	0	0	0	0	0	0	⟶	KO FASL	1	0	0	0	0	0	0	0	0	0	0	0	0	0	0	0	0	0	0	0	0	0	0	0	0	0	0
Unperturbed	1	0	0	0	0	0	0	0	0	0	0	0	0	0	0	0	0	0	0	0	0	0	0	0	0	0	0	⟶	KO IKK	1	0	0	0	0	0	0	0	0	0	0	0	0	0	0	0	0	0	0	0	0	0	0	0	0	0	0
Unperturbed	1	0	0	0	0	0	0	0	0	0	0	0	0	0	0	0	0	0	0	0	0	0	0	0	0	0	0	⟶	KO MOMP	1	0	0	0	0	0	0	0	0	0	0	0	0	0	0	0	0	0	0	0	0	0	0	0	0	0	0
Unperturbed	1	0	0	0	0	0	0	0	0	0	0	0	0	0	0	0	0	0	0	0	0	0	0	0	0	0	0	⟶	KO MPT	1	0	0	0	0	0	0	0	0	0	0	0	0	0	0	0	0	0	0	0	0	0	0	0	0	0	0
Unperturbed	1	0	0	0	0	0	0	0	0	0	0	0	0	0	0	0	0	0	0	0	0	0	0	0	0	0	0	⟶	KO NFkB	1	0	0	0	0	0	0	0	0	0	0	0	0	0	0	0	0	0	0	0	0	0	0	0	0	0	0
Unperturbed	1	0	0	0	0	0	0	0	0	0	0	0	0	0	0	0	0	0	0	0	0	0	0	0	0	0	0	⟶	KO NonACD	1	0	0	0	0	0	0	0	0	0	0	0	0	0	0	0	0	0	0	0	0	0	0	0	0	0	0
Unperturbed	1	0	0	0	0	0	0	0	0	0	0	0	0	0	0	0	0	0	0	0	0	0	0	0	0	0	0	⟶	KO RIP1	1	0	0	0	0	0	0	0	0	0	0	0	0	0	0	0	0	0	0	0	0	0	0	0	0	0	0
Unperturbed	1	0	0	0	0	0	0	0	0	0	0	0	0	0	0	0	0	0	0	0	0	0	0	0	0	0	0	⟶	KO RIP1K	1	0	0	0	0	0	0	0	0	0	0	0	0	0	0	0	0	0	0	0	0	0	0	0	0	0	0
Unperturbed	1	0	0	0	0	0	0	0	0	0	0	0	0	0	0	0	0	0	0	0	0	0	0	0	0	0	0	⟶	KO RIP1ub	1	0	0	0	0	0	0	0	0	0	0	0	0	0	0	0	0	0	0	0	0	0	0	0	0	0	0
Unperturbed	1	0	0	0	0	0	0	0	0	0	0	0	0	0	0	0	0	0	0	0	0	0	0	0	0	0	0	⟶	KO ROS	1	0	0	0	0	0	0	0	0	0	0	0	0	0	0	0	0	0	0	0	0	0	0	0	0	0	0
Unperturbed	1	0	0	0	0	0	0	0	0	0	0	0	0	0	0	0	0	0	0	0	0	0	0	0	0	0	0	⟶	KO SMAC	1	0	0	0	0	0	0	0	0	0	0	0	0	0	0	0	0	0	0	0	0	0	0	0	0	0	0
Unperturbed	1	0	0	0	0	0	0	0	0	0	0	0	0	0	0	0	0	0	0	0	0	0	0	0	0	0	0	⟶	KO Survival	1	0	0	0	0	0	0	0	0	0	0	0	0	0	0	0	0	0	0	0	0	0	0	0	0	0	0
Unperturbed	1	0	0	0	0	0	0	0	0	0	0	0	0	0	0	0	0	0	0	0	0	0	0	0	0	0	0	⟶	KO TNF	1	0	0	0	0	0	0	0	0	0	0	0	0	0	0	0	0	0	0	0	0	0	0	0	0	0	0
Unperturbed	1	0	0	0	0	0	0	0	0	0	0	0	0	0	0	0	0	0	0	0	0	0	0	0	0	0	0	⟶	KO TNFR	1	0	0	0	0	0	0	0	0	0	0	0	0	0	0	0	0	0	0	0	0	0	0	0	0	0	0
Unperturbed	1	0	0	0	0	0	0	0	0	0	0	0	0	0	0	0	0	0	0	0	0	0	0	0	0	0	0	⟶	KO XIAP	1	0	0	0	0	0	0	0	0	0	0	0	0	0	0	0	0	0	0	0	0	0	0	0	0	0	0
Unperturbed	1	0	0	0	0	0	0	0	0	0	0	0	0	0	0	0	0	0	0	0	0	0	0	0	0	0	0	⟶	KO cFLIP	1	0	0	0	0	0	0	0	0	0	0	0	0	0	0	0	0	0	0	0	0	0	0	0	0	0	0
Unperturbed	1	0	0	0	0	0	0	0	0	0	0	0	0	0	0	0	0	0	0	0	0	0	0	0	0	0	0	⟶	KO cIAP	1	0	0	0	0	0	0	0	0	0	0	0	0	0	0	0	0	0	0	0	0	0	0	0	0	0	0
Unperturbed	1	1	1	1	0	1	1	1	0	0	0	0	1	0	0	0	0	0	0	0	1	0	0	0	0	0	0	⟶	OE ATP	1	1	1	1	0	1	1	1	0	0	0	0	1	0	0	0	0	0	0	0	1	0	0	0	0	0	0
Unperturbed	1	1	1	1	0	1	1	1	0	0	0	0	1	0	0	0	0	0	0	0	1	0	0	0	0	0	0	⟶	OE Apoptosis	1	1	1	1	0	1	1	1	0	0	0	0	1	0	0	0	0	0	0	0	1	0	0	0	0	0	0
Unperturbed	1	1	1	1	0	1	1	1	0	0	0	0	1	0	0	0	0	0	0	0	1	0	0	0	0	0	0	⟶	OE Apoptosome	1	1	1	1	0	1	1	1	0	0	0	0	1	0	0	0	0	0	0	0	1	0	0	0	0	0	0
Unperturbed	1	1	1	1	0	1	1	1	0	0	0	0	1	0	0	0	0	0	0	0	1	0	0	0	0	0	0	⟶	OE BAX	1	1	1	1	0	1	1	1	0	0	0	0	1	0	0	0	0	0	0	0	1	0	0	0	0	0	0
Unperturbed	1	1	1	1	0	1	1	1	0	0	0	0	1	0	0	0	0	0	0	0	1	0	0	0	0	0	0	⟶	OE BCL2	1	0	0	0	1	0	0	0	0	0	0	0	0	0	0	0	0	0	0	0	0	0	0	0	0	0	0
Unperturbed	1	1	1	1	0	1	1	1	0	0	0	0	1	0	0	0	0	0	0	0	1	0	0	0	0	0	0	⟶	OE CASP3	1	1	1	1	0	1	1	1	0	0	0	0	1	0	0	0	0	0	0	0	1	0	0	0	0	0	0
Unperturbed	1	1	1	1	0	1	1	1	0	0	0	0	1	0	0	0	0	0	0	0	1	0	0	0	0	0	0	⟶	OE CASP8	1	1	1	1	0	1	1	1	0	0	0	0	1	0	0	0	0	0	0	0	1	0	0	0	0	0	0
Unperturbed	1	1	1	1	0	1	1	1	0	0	0	0	1	0	0	0	0	0	0	0	1	0	0	0	0	0	0	⟶	OE Cyt_c	1	1	1	1	0	1	1	1	0	0	0	0	1	0	0	0	0	0	0	0	1	0	0	0	0	0	0
Unperturbed	1	1	1	1	0	1	1	1	0	0	0	0	1	0	0	0	0	0	0	0	1	0	0	0	0	0	0	⟶	OE DISC-FAS	1	1	1	1	0	1	1	1	1	0	0	0	1	0	0	0	0	0	0	0	1	0	0	0	0	0	0
Unperturbed	1	1	1	1	0	1	1	1	0	0	0	0	1	0	0	0	0	0	0	0	1	0	0	0	0	0	0	⟶	OE DISC-TNF	1	1	1	1	0	1	1	1	0	1	0	0	1	0	0	0	0	0	0	0	1	0	0	0	0	0	0
Unperturbed	1	1	1	1	0	1	1	1	0	0	0	0	1	0	0	0	0	0	0	0	1	0	0	0	0	0	0	⟶	OE FASL	1	1	1	1	0	1	1	1	1	0	1	0	1	0	0	0	0	0	0	0	1	0	0	0	0	0	0
Unperturbed	1	1	1	1	0	1	1	1	0	0	0	0	1	0	0	0	0	0	0	0	1	0	0	0	0	0	0	⟶	OE IKK	1	1	1	1	0	1	1	1	0	0	0	1	1	0	0	0	0	0	0	0	1	0	0	0	0	0	0
Unperturbed	1	1	1	1	0	1	1	1	0	0	0	0	1	0	0	0	0	0	0	0	1	0	0	0	0	0	0	⟶	OE MOMP	1	1	1	1	0	1	1	1	0	0	0	0	1	0	0	0	0	0	0	0	1	0	0	0	0	0	0
Unperturbed	1	1	1	1	0	1	1	1	0	0	0	0	1	0	0	0	0	0	0	0	1	0	0	0	0	0	0	⟶	OE MPT	0	0	0	0	0	0	0	1	0	0	0	0	1	1	0	1	0	0	0	1	1	0	0	0	0	0	0

Initial attractor																													Final attractor																											
Perturbation	ATP	Apoptosis	Apoptosome	BAX	BCL2	CASP3	CASP8	Cyt_c	DISC-FAS	DISC-TNF	FASL	IKK	MOMP	MPT	NFkB	NonACD	RIP1	RIP1K	RIP1ub	ROS	SMAC	Survival	TNF	TNFR	XIAP	cFLIP	cIAP		Perturbation	ATP	Apoptosis	Apoptosome	BAX	BCL2	CASP3	CASP8	Cyt_c	DISC-FAS	DISC-TNF	FASL	IKK	MOMP	MPT	NFkB	NonACD	RIP1	RIP1K	RIP1ub	ROS	SMAC	Survival	TNF	TNFR	XIAP	cFLIP	cIAP
Unperturbed	1	1	1	1	0	1	1	1	0	0	0	0	1	0	0	0	0	0	0	0	1	0	0	0	0	0	0	⟶	OE NFkB	1	0	0	0	1	0	0	0	0	0	0	0	0	0	1	0	0	0	0	0	0	1	0	0	1	1	1
Unperturbed	1	1	1	1	0	1	1	1	0	0	0	0	1	0	0	0	0	0	0	0	1	0	0	0	0	0	0	⟶	OE NonACD	1	1	1	1	0	1	1	1	0	0	0	0	1	0	0	1	0	0	0	0	1	0	0	0	0	0	0
Unperturbed	1	1	1	1	0	1	1	1	0	0	0	0	1	0	0	0	0	0	0	0	1	0	0	0	0	0	0	⟶	OE RIP1	0	0	0	0	0	0	0	1	0	0	0	0	1	1	0	1	1	1	0	1	1	0	0	0	0	0	0
Unperturbed	1	1	1	1	0	1	1	1	0	0	0	0	1	0	0	0	0	0	0	0	1	0	0	0	0	0	0	⟶	OE RIP1K	0	0	0	0	0	0	0	1	0	0	0	0	1	1	0	1	0	1	0	1	1	0	0	0	0	0	0
Unperturbed	1	1	1	1	0	1	1	1	0	0	0	0	1	0	0	0	0	0	0	0	1	0	0	0	0	0	0	⟶	OE RIP1ub	1	1	1	1	0	1	1	1	0	0	0	1	1	0	0	0	0	0	1	0	1	0	0	0	0	0	0
Unperturbed	1	1	1	1	0	1	1	1	0	0	0	0	1	0	0	0	0	0	0	0	1	0	0	0	0	0	0	⟶	OE ROS	0	0	0	0	0	0	0	1	0	0	0	0	1	1	0	1	0	0	0	1	1	0	0	0	0	0	0
Unperturbed	1	1	1	1	0	1	1	1	0	0	0	0	1	0	0	0	0	0	0	0	1	0	0	0	0	0	0	⟶	OE SMAC	1	1	1	1	0	1	1	1	0	0	0	0	1	0	0	0	0	0	0	0	1	0	0	0	0	0	0
Unperturbed	1	1	1	1	0	1	1	1	0	0	0	0	1	0	0	0	0	0	0	0	1	0	0	0	0	0	0	⟶	OE Survival	1	1	1	1	0	1	1	1	0	0	0	0	1	0	0	0	0	0	0	0	1	1	0	0	0	0	0
Unperturbed	1	1	1	1	0	1	1	1	0	0	0	0	1	0	0	0	0	0	0	0	1	0	0	0	0	0	0	⟶	OE TNF	1	1	1	1	0	1	1	1	0	1	0	0	1	0	0	0	0	0	0	0	1	0	1	1	0	0	0
Unperturbed	1	1	1	1	0	1	1	1	0	0	0	0	1	0	0	0	0	0	0	0	1	0	0	0	0	0	0	⟶	OE TNFR	1	1	1	1	0	1	1	1	0	1	0	0	1	0	0	0	0	0	0	0	1	0	0	1	0	0	0
Unperturbed	1	1	1	1	0	1	1	1	0	0	0	0	1	0	0	0	0	0	0	0	1	0	0	0	0	0	0	⟶	OE XIAP	1	0	0	0	0	0	0	0	0	0	0	0	0	0	0	0	0	0	0	0	0	0	0	0	1	0	0
Unperturbed	1	1	1	1	0	1	1	1	0	0	0	0	1	0	0	0	0	0	0	0	1	0	0	0	0	0	0	⟶	OE cFLIP	1	0	0	0	0	0	0	0	0	0	0	0	0	0	0	0	0	0	0	0	0	0	0	0	0	1	0
Unperturbed	1	1	1	1	0	1	1	1	0	0	0	0	1	0	0	0	0	0	0	0	1	0	0	0	0	0	0	⟶	OE cIAP	1	1	1	1	0	1	1	1	0	0	0	0	1	0	0	0	0	0	0	0	1	0	0	0	0	0	1
Unperturbed	1	1	1	1	0	1	1	1	0	0	0	0	1	0	0	0	0	0	0	0	1	0	0	0	0	0	0	⟶	KO ATP	0	0	0	0	0	0	0	0	0	0	0	0	0	0	0	1	0	0	0	0	0	0	0	0	0	0	0
Unperturbed	1	1	1	1	0	1	1	1	0	0	0	0	1	0	0	0	0	0	0	0	1	0	0	0	0	0	0	⟶	KO Apoptosis	1	0	1	1	0	1	1	1	0	0	0	0	1	0	0	0	0	0	0	0	1	0	0	0	0	0	0
Unperturbed	1	1	1	1	0	1	1	1	0	0	0	0	1	0	0	0	0	0	0	0	1	0	0	0	0	0	0	⟶	KO Apoptosome	1	0	0	0	0	0	0	0	0	0	0	0	0	0	0	0	0	0	0	0	0	0	0	0	0	0	0
Unperturbed	1	1	1	1	0	1	1	1	0	0	0	0	1	0	0	0	0	0	0	0	1	0	0	0	0	0	0	⟶	KO BAX	1	0	0	0	0	0	0	0	0	0	0	0	0	0	0	0	0	0	0	0	0	0	0	0	0	0	0
Unperturbed	1	1	1	1	0	1	1	1	0	0	0	0	1	0	0	0	0	0	0	0	1	0	0	0	0	0	0	⟶	KO BCL2	1	1	1	1	0	1	1	1	0	0	0	0	1	0	0	0	0	0	0	0	1	0	0	0	0	0	0
Unperturbed	1	1	1	1	0	1	1	1	0	0	0	0	1	0	0	0	0	0	0	0	1	0	0	0	0	0	0	⟶	KO CASP3	1	0	0	0	0	0	0	0	0	0	0	0	0	0	0	0	0	0	0	0	0	0	0	0	0	0	0
Unperturbed	1	1	1	1	0	1	1	1	0	0	0	0	1	0	0	0	0	0	0	0	1	0	0	0	0	0	0	⟶	KO CASP8	1	0	0	0	0	0	0	0	0	0	0	0	0	0	0	0	0	0	0	0	0	0	0	0	0	0	0
Unperturbed	1	1	1	1	0	1	1	1	0	0	0	0	1	0	0	0	0	0	0	0	1	0	0	0	0	0	0	⟶	KO Cyt_c	1	0	0	0	0	0	0	0	0	0	0	0	0	0	0	0	0	0	0	0	0	0	0	0	0	0	0
Unperturbed	1	1	1	1	0	1	1	1	0	0	0	0	1	0	0	0	0	0	0	0	1	0	0	0	0	0	0	⟶	KO DISC-FAS	1	1	1	1	0	1	1	1	0	0	0	0	1	0	0	0	0	0	0	0	1	0	0	0	0	0	0
Unperturbed	1	1	1	1	0	1	1	1	0	0	0	0	1	0	0	0	0	0	0	0	1	0	0	0	0	0	0	⟶	KO DISC-TNF	1	1	1	1	0	1	1	1	0	0	0	0	1	0	0	0	0	0	0	0	1	0	0	0	0	0	0
Unperturbed	1	1	1	1	0	1	1	1	0	0	0	0	1	0	0	0	0	0	0	0	1	0	0	0	0	0	0	⟶	KO FASL	1	1	1	1	0	1	1	1	0	0	0	0	1	0	0	0	0	0	0	0	1	0	0	0	0	0	0
Unperturbed	1	1	1	1	0	1	1	1	0	0	0	0	1	0	0	0	0	0	0	0	1	0	0	0	0	0	0	⟶	KO IKK	1	1	1	1	0	1	1	1	0	0	0	0	1	0	0	0	0	0	0	0	1	0	0	0	0	0	0
Unperturbed	1	1	1	1	0	1	1	1	0	0	0	0	1	0	0	0	0	0	0	0	1	0	0	0	0	0	0	⟶	KO MOMP	1	0	0	0	0	0	0	0	0	0	0	0	0	0	0	0	0	0	0	0	0	0	0	0	0	0	0
Unperturbed	1	1	1	1	0	1	1	1	0	0	0	0	1	0	0	0	0	0	0	0	1	0	0	0	0	0	0	⟶	KO MPT	1	1	1	1	0	1	1	1	0	0	0	0	1	0	0	0	0	0	0	0	1	0	0	0	0	0	0
Unperturbed	1	1	1	1	0	1	1	1	0	0	0	0	1	0	0	0	0	0	0	0	1	0	0	0	0	0	0	⟶	KO NFkB	1	1	1	1	0	1	1	1	0	0	0	0	1	0	0	0	0	0	0	0	1	0	0	0	0	0	0
Unperturbed	1	1	1	1	0	1	1	1	0	0	0	0	1	0	0	0	0	0	0	0	1	0	0	0	0	0	0	⟶	KO NonACD	1	1	1	1	0	1	1	1	0	0	0	0	1	0	0	0	0	0	0	0	1	0	0	0	0	0	0
Unperturbed	1	1	1	1	0	1	1	1	0	0	0	0	1	0	0	0	0	0	0	0	1	0	0	0	0	0	0	⟶	KO RIP1	1	1	1	1	0	1	1	1	0	0	0	0	1	0	0	0	0	0	0	0	1	0	0	0	0	0	0
Unperturbed	1	1	1	1	0	1	1	1	0	0	0	0	1	0	0	0	0	0	0	0	1	0	0	0	0	0	0	⟶	KO RIP1K	1	1	1	1	0	1	1	1	0	0	0	0	1	0	0	0	0	0	0	0	1	0	0	0	0	0	0
Unperturbed	1	1	1	1	0	1	1	1	0	0	0	0	1	0	0	0	0	0	0	0	1	0	0	0	0	0	0	⟶	KO RIP1ub	1	1	1	1	0	1	1	1	0	0	0	0	1	0	0	0	0	0	0	0	1	0	0	0	0	0	0
Unperturbed	1	1	1	1	0	1	1	1	0	0	0	0	1	0	0	0	0	0	0	0	1	0	0	0	0	0	0	⟶	KO ROS	1	1	1	1	0	1	1	1	0	0	0	0	1	0	0	0	0	0	0	0	1	0	0	0	0	0	0
Unperturbed	1	1	1	1	0	1	1	1	0	0	0	0	1	0	0	0	0	0	0	0	1	0	0	0	0	0	0	⟶	KO SMAC	1	1	1	1	0	1	1	1	0	0	0	0	1	0	0	0	0	0	0	0	0	0	0	0	0	0	0
Unperturbed	1	1	1	1	0	1	1	1	0	0	0	0	1	0	0	0	0	0	0	0	1	0	0	0	0	0	0	⟶	KO Survival	1	1	1	1	0	1	1	1	0	0	0	0	1	0	0	0	0	0	0	0	1	0	0	0	0	0	0
Unperturbed	1	1	1	1	0	1	1	1	0	0	0	0	1	0	0	0	0	0	0	0	1	0	0	0	0	0	0	⟶	KO TNF	1	1	1	1	0	1	1	1	0	0	0	0	1	0	0	0	0	0	0	0	1	0	0	0	0	0	0
Unperturbed	1	1	1	1	0	1	1	1	0	0	0	0	1	0	0	0	0	0	0	0	1	0	0	0	0	0	0	⟶	KO TNFR	1	1	1	1	0	1	1	1	0	0	0	0	1	0	0	0	0	0	0	0	1	0	0	0	0	0	0
Unperturbed	1	1	1	1	0	1	1	1	0	0	0	0	1	0	0	0	0	0	0	0	1	0	0	0	0	0	0	⟶	KO XIAP	1	1	1	1	0	1	1	1	0	0	0	0	1	0	0	0	0	0	0	0	1	0	0	0	0	0	0
Unperturbed	1	1	1	1	0	1	1	1	0	0	0	0	1	0	0	0	0	0	0	0	1	0	0	0	0	0	0	⟶	KO cFLIP	1	1	1	1	0	1	1	1	0	0	0	0	1	0	0	0	0	0	0	0	1	0	0	0	0	0	0
Unperturbed	1	1	1	1	0	1	1	1	0	0	0	0	1	0	0	0	0	0	0	0	1	0	0	0	0	0	0	⟶	KO cIAP	1	1	1	1	0	1	1	1	0	0	0	0	1	0	0	0	0	0	0	0	1	0	0	0	0	0	0
Unperturbed	0	0	0	0	0	0	0	1	0	0	0	0	1	1	0	1	0	0	0	1	1	0	0	0	0	0	0	⟶	OE ATP	1	1	1	1	0	1	1	1	0	0	0	0	1	1	0	0	0	0	0	1	1	0	0	0	0	0	0
Unperturbed	0	0	0	0	0	0	0	1	0	0	0	0	1	1	0	1	0	0	0	1	1	0	0	0	0	0	0	⟶	OE Apoptosis	0	1	0	0	0	0	0	1	0	0	0	0	1	1	0	1	0	0	0	1	1	0	0	0	0	0	0
Unperturbed	0	0	0	0	0	0	0	1	0	0	0	0	1	1	0	1	0	0	0	1	1	0	0	0	0	0	0	⟶	OE Apoptosome	0	1	1	1	0	1	1	1	0	0	0	0	1	1	0	1	0	0	0	1	1	0	0	0	0	0	0
Unperturbed	0	0	0	0	0	0	0	1	0	0	0	0	1	1	0	1	0	0	0	1	1	0	0	0	0	0	0	⟶	OE BAX	0	0	0	1	0	0	0	1	0	0	0	0	1	1	0	1	0	0	0	1	1	0	0	0	0	0	0
Unperturbed	0	0	0	0	0	0	0	1	0	0	0	0	1	1	0	1	0	0	0	1	1	0	0	0	0	0	0	⟶	OE BCL2	1	0	0	0	1	0	0	0	0	0	0	0	0	0	0	0	0	0	0	0	0	0	0	0	0	0	0
Unperturbed	0	0	0	0	0	0	0	1	0	0	0	0	1	1	0	1	0	0	0	1	1	0	0	0	0	0	0	⟶	OE CASP3	0	1	0	1	0	1	1	1	0	0	0	0	1	1	0	1	0	0	0	1	1	0	0	0	0	0	0

	Initial attractor																													Final attractor																									
Perturbation	ATP	Apoptosis	Apoptosome	BAX	BCL2	CASP3	CASP8	Cyt_c	DISC-FAS	DISC-TNF	FASL	IKK	MOMP	MPT	NFkB	NonACD	RIP1	RIP1K	RIP1ub	ROS	SMAC	Survival	TNF	TNFR	XIAP	cFLIP	cIAP		Perturbation	ATP	Apoptosis	Apoptosome	BAX	BCL2	CASP3	CASP8	Cyt_c	DISC-FAS	DISC-TNF	FASL	IKK	MOMP	MPT	NFkB	NonACD	RIP1	RIP1K	RIP1ub	ROS	SMAC	Survival	TNF	TNFR	XIAP	cFLIP	cIAP
Unperturbed	0	0	0	0	0	0	0	1	0	0	0	0	1	1	0	1	0	0	0	1	1	0	0	0	0	0	0	$\longrightarrow$	OE CASP8	0	0	0	1	0	0	1	1	0	0	0	0	1	1	0	1	0	0	0	1	1	0	0	0	0	0	0
Unperturbed	0	0	0	0	0	0	0	1	0	0	0	0	1	1	0	1	0	0	0	1	1	0	0	0	0	0	0	$\longrightarrow$	OE Cyt_c	0	0	0	0	0	0	0	1	0	0	0	0	1	1	0	1	0	0	0	1	1	0	0	0	0	0	0
Unperturbed	0	0	0	0	0	0	0	1	0	0	0	0	1	1	0	1	0	0	0	1	1	0	0	0	0	0	0	$\longrightarrow$	OE DISC-FAS	0	0	0	1	0	0	1	1	1	0	0	0	1	1	0	1	0	0	0	1	1	0	0	0	0	0	0
Unperturbed	0	0	0	0	0	0	0	1	0	0	0	0	1	1	0	1	0	0	0	1	1	0	0	0	0	0	0	$\longrightarrow$	OE DISC-TNF	0	0	0	1	0	0	1	1	0	1	0	0	1	1	0	1	0	0	0	1	1	0	0	0	0	0	0
Unperturbed	0	0	0	0	0	0	0	1	0	0	0	0	1	1	0	1	0	0	0	1	1	0	0	0	0	0	0	$\longrightarrow$	OE FASL	0	0	0	1	0	0	1	1	1	0	1	0	1	1	0	1	0	0	0	1	1	0	0	0	0	0	0
Unperturbed	0	0	0	0	0	0	0	1	0	0	0	0	1	1	0	1	0	0	0	1	1	0	0	0	0	0	0	$\longrightarrow$	OE IKK	1	0	0	0	1	0	0	0	0	0	0	1	0	0	1	0	0	0	0	0	0	1	0	0	1	1	1
Unperturbed	0	0	0	0	0	0	0	1	0	0	0	0	1	1	0	1	0	0	0	1	1	0	0	0	0	0	0	$\longrightarrow$	OE IKK	1	1	1	1	0	1	1	1	0	0	0	1	1	0	0	0	0	0	0	0	1	0	0	0	0	0	0
Unperturbed	0	0	0	0	0	0	0	1	0	0	0	0	1	1	0	1	0	0	0	1	1	0	0	0	0	0	0	$\longrightarrow$	OE MOMP	0	0	0	0	0	0	0	1	0	0	0	0	1	1	0	1	0	0	0	1	1	0	0	0	0	0	0
Unperturbed	0	0	0	0	0	0	0	1	0	0	0	0	1	1	0	1	0	0	0	1	1	0	0	0	0	0	0	$\longrightarrow$	OE MPT	0	0	0	0	0	0	0	1	0	0	0	0	1	1	0	1	0	0	0	1	1	0	0	0	0	0	0
Unperturbed	0	0	0	0	0	0	0	1	0	0	0	0	1	1	0	1	0	0	0	1	1	0	0	0	0	0	0	$\longrightarrow$	OE NFkB	1	0	0	0	1	0	0	0	0	0	0	0	0	0	1	0	0	0	0	0	0	1	0	0	1	1	1
Unperturbed	0	0	0	0	0	0	0	1	0	0	0	0	1	1	0	1	0	0	0	1	1	0	0	0	0	0	0	$\longrightarrow$	OE NonACD	0	0	0	0	0	0	0	1	0	0	0	0	1	1	0	1	0	0	0	1	1	0	0	0	0	0	0
Unperturbed	0	0	0	0	0	0	0	1	0	0	0	0	1	1	0	1	0	0	0	1	1	0	0	0	0	0	0	$\longrightarrow$	OE RIP1	0	0	0	0	0	0	0	1	0	0	0	0	1	1	0	1	1	1	0	1	1	0	0	0	0	0	0
Unperturbed	0	0	0	0	0	0	0	1	0	0	0	0	1	1	0	1	0	0	0	1	1	0	0	0	0	0	0	$\longrightarrow$	OE RIP1K	0	0	0	0	0	0	0	1	0	0	0	0	1	1	0	1	0	1	0	1	1	0	0	0	0	0	0
Unperturbed	0	0	0	0	0	0	0	1	0	0	0	0	1	1	0	1	0	0	0	1	1	0	0	0	0	0	0	$\longrightarrow$	OE RIP1ub	1	0	0	0	1	0	0	0	0	0	0	1	0	0	1	0	0	0	1	0	0	1	0	0	1	1	1
Unperturbed	0	0	0	0	0	0	0	1	0	0	0	0	1	1	0	1	0	0	0	1	1	0	0	0	0	0	0	$\longrightarrow$	OE RIP1ub	1	1	1	1	0	1	1	1	0	0	0	1	1	0	0	0	0	0	1	0	1	0	0	0	0	0	0
Unperturbed	0	0	0	0	0	0	0	1	0	0	0	0	1	1	0	1	0	0	0	1	1	0	0	0	0	0	0	$\longrightarrow$	OE ROS	0	0	0	0	0	0	0	1	0	0	0	0	1	1	0	1	0	0	0	1	1	0	0	0	0	0	0
Unperturbed	0	0	0	0	0	0	0	1	0	0	0	0	1	1	0	1	0	0	0	1	1	0	0	0	0	0	0	$\longrightarrow$	OE SMAC	0	0	0	0	0	0	0	1	0	0	0	0	1	1	0	1	0	0	0	1	1	0	0	0	0	0	0
Unperturbed	0	0	0	0	0	0	0	1	0	0	0	0	1	1	0	1	0	0	0	1	1	0	0	0	0	0	0	$\longrightarrow$	OE Survival	0	0	0	0	0	0	0	1	0	0	0	0	1	1	0	1	0	0	0	1	1	1	0	0	0	0	0
Unperturbed	0	0	0	0	0	0	0	1	0	0	0	0	1	1	0	1	0	0	0	1	1	0	0	0	0	0	0	$\longrightarrow$	OE TNF	0	0	0	1	0	0	1	1	0	1	0	0	1	1	0	1	0	0	0	1	1	0	1	1	0	0	0
Unperturbed	0	0	0	0	0	0	0	1	0	0	0	0	1	1	0	1	0	0	0	1	1	0	0	0	0	0	0	$\longrightarrow$	OE TNFR	0	0	0	1	0	0	1	1	0	1	0	0	1	1	0	1	0	0	0	1	1	0	0	1	0	0	0
Unperturbed	0	0	0	0	0	0	0	1	0	0	0	0	1	1	0	1	0	0	0	1	1	0	0	0	0	0	0	$\longrightarrow$	OE XIAP	0	0	0	0	0	0	0	1	0	0	0	0	1	1	0	1	0	0	0	1	1	0	0	0	1	0	0
Unperturbed	0	0	0	0	0	0	0	1	0	0	0	0	1	1	0	1	0	0	0	1	1	0	0	0	0	0	0	$\longrightarrow$	OE cFLIP	0	0	0	0	0	0	0	1	0	0	0	0	1	1	0	1	0	0	0	1	1	0	0	0	0	1	0
Unperturbed	0	0	0	0	0	0	0	1	0	0	0	0	1	1	0	1	0	0	0	1	1	0	0	0	0	0	0	$\longrightarrow$	OE cIAP	0	0	0	0	0	0	0	1	0	0	0	0	1	1	0	1	0	0	0	1	1	0	0	0	0	0	1
Unperturbed	0	0	0	0	0	0	0	1	0	0	0	0	1	1	0	1	0	0	0	1	1	0	0	0	0	0	0	$\longrightarrow$	KO ATP	0	0	0	0	0	0	0	1	0	0	0	0	1	1	0	1	0	0	0	1	1	0	0	0	0	0	0
Unperturbed	0	0	0	0	0	0	0	1	0	0	0	0	1	1	0	1	0	0	0	1	1	0	0	0	0	0	0	$\longrightarrow$	KO Apoptosis	0	0	0	0	0	0	0	1	0	0	0	0	1	1	0	1	0	0	0	1	1	0	0	0	0	0	0
Unperturbed	0	0	0	0	0	0	0	1	0	0	0	0	1	1	0	1	0	0	0	1	1	0	0	0	0	0	0	$\longrightarrow$	KO Apoptosome	0	0	0	0	0	0	0	1	0	0	0	0	1	1	0	1	0	0	0	1	1	0	0	0	0	0	0
Unperturbed	0	0	0	0	0	0	0	1	0	0	0	0	1	1	0	1	0	0	0	1	1	0	0	0	0	0	0	$\longrightarrow$	KO BAX	0	0	0	0	0	0	0	1	0	0	0	0	1	1	0	1	0	0	0	1	1	0	0	0	0	0	0
Unperturbed	0	0	0	0	0	0	0	1	0	0	0	0	1	1	0	1	0	0	0	1	1	0	0	0	0	0	0	$\longrightarrow$	KO BCL2	0	0	0	0	0	0	0	1	0	0	0	0	1	1	0	1	0	0	0	1	1	0	0	0	0	0	0
Unperturbed	0	0	0	0	0	0	0	1	0	0	0	0	1	1	0	1	0	0	0	1	1	0	0	0	0	0	0	$\longrightarrow$	KO CASP3	0	0	0	0	0	0	0	1	0	0	0	0	1	1	0	1	0	0	0	1	1	0	0	0	0	0	0
Unperturbed	0	0	0	0	0	0	0	1	0	0	0	0	1	1	0	1	0	0	0	1	1	0	0	0	0	0	0	$\longrightarrow$	KO CASP8	0	0	0	0	0	0	0	1	0	0	0	0	1	1	0	1	0	0	0	1	1	0	0	0	0	0	0
Unperturbed	0	0	0	0	0	0	0	1	0	0	0	0	1	1	0	1	0	0	0	1	1	0	0	0	0	0	0	$\longrightarrow$	KO Cyt_c	0	0	0	0	0	0	0	0	0	0	0	0	1	1	0	1	0	0	0	1	1	0	0	0	0	0	0
Unperturbed	0	0	0	0	0	0	0	1	0	0	0	0	1	1	0	1	0	0	0	1	1	0	0	0	0	0	0	$\longrightarrow$	KO DISC-FAS	0	0	0	0	0	0	0	1	0	0	0	0	1	1	0	1	0	0	0	1	1	0	0	0	0	0	0
Unperturbed	0	0	0	0	0	0	0	1	0	0	0	0	1	1	0	1	0	0	0	1	1	0	0	0	0	0	0	$\longrightarrow$	KO DISC-TNF	0	0	0	0	0	0	0	1	0	0	0	0	1	1	0	1	0	0	0	1	1	0	0	0	0	0	0
Unperturbed	0	0	0	0	0	0	0	1	0	0	0	0	1	1	0	1	0	0	0	1	1	0	0	0	0	0	0	$\longrightarrow$	KO FASL	0	0	0	0	0	0	0	1	0	0	0	0	1	1	0	1	0	0	0	1	1	0	0	0	0	0	0
Unperturbed	0	0	0	0	0	0	0	1	0	0	0	0	1	1	0	1	0	0	0	1	1	0	0	0	0	0	0	$\longrightarrow$	KO IKK	0	0	0	0	0	0	0	1	0	0	0	0	1	1	0	1	0	0	0	1	1	0	0	0	0	0	0
Unperturbed	0	0	0	0	0	0	0	1	0	0	0	0	1	1	0	1	0	0	0	1	1	0	0	0	0	0	0	$\longrightarrow$	KO MOMP	0	0	0	0	0	0	0	0	0	0	0	0	0	1	0	1	0	0	0	1	0	0	0	0	0	0	0
Unperturbed	0	0	0	0	0	0	0	1	0	0	0	0	1	1	0	1	0	0	0	1	1	0	0	0	0	0	0	$\longrightarrow$	KO MPT	1	0	0	0	0	0	0	0	0	0	0	0	0	0	0	0	0	0	0	0	0	0	0	0	0	0	0
Unperturbed	0	0	0	0	0	0	0	1	0	0	0	0	1	1	0	1	0	0	0	1	1	0	0	0	0	0	0	$\longrightarrow$	KO MPT	1	1	1	1	0	1	1	1	0	0	0	0	1	0	0	0	0	0	0	0	1	0	0	0	0	0	0
Unperturbed	0	0	0	0	0	0	0	1	0	0	0	0	1	1	0	1	0	0	0	1	1	0	0	0	0	0	0	$\longrightarrow$	KO NFkB	0	0	0	0	0	0	0	1	0	0	0	0	1	1	0	1	0	0	0	1	1	0	0	0	0	0	0
Unperturbed	0	0	0	0	0	0	0	1	0	0	0	0	1	1	0	1	0	0	0	1	1	0	0	0	0	0	0	$\longrightarrow$	KO NonACD	0	0	0	0	0	0	0	1	0	0	0	0	1	1	0	0	0	0	0	1	1	0	0	0	0	0	0
Unperturbed	0	0	0	0	0	0	0	1	0	0	0	0	1	1	0	1	0	0	0	1	1	0	0	0	0	0	0	$\longrightarrow$	KO RIP1	0	0	0	0	0	0	0	1	0	0	0	0	1	1	0	1	0	0	0	1	1	0	0	0	0	0	0
Unperturbed	0	0	0	0	0	0	0	1	0	0	0	0	1	1	0	1	0	0	0	1	1	0	0	0	0	0	0	$\longrightarrow$	KO RIP1K	0	0	0	0	0	0	0	1	0	0	0	0	1	1	0	1	0	0	0	1	1	0	0	0	0	0	0
Unperturbed	0	0	0	0	0	0	0	1	0	0	0	0	1	1	0	1	0	0	0	1	1	0	0	0	0	0	0	$\longrightarrow$	KO RIP1ub	0	0	0	0	0	0	0	1	0	0	0	0	1	1	0	1	0	0	0	1	1	0	0	0	0	0	0
Unperturbed	0	0	0	0	0	0	0	1	0	0	0	0	1	1	0	1	0	0	0	1	1	0	0	0	0	0	0	$\longrightarrow$	KO ROS	1	0	0	0	0	0	0	0	0	0	0	0	0	0	0	0	0	0	0	0	0	0	0	0	0	0	0
Unperturbed	0	0	0	0	0	0	0	1	0	0	0	0	1	1	0	1	0	0	0	1	1	0	0	0	0	0	0	$\longrightarrow$	KO ROS	1	1	1	1	0	1	1	1	0	0	0	0	1	0	0	0	0	0	0	0	1	0	0	0	0	0	0
Unperturbed	0	0	0	0	0	0	0	1	0	0	0	0	1	1	0	1	0	0	0	1	1	0	0	0	0	0	0	$\longrightarrow$	KO SMAC	0	0	0	0	0	0	0	1	0	0	0	0	1	1	0	1	0	0	0	1	0	0	0	0	0	0	0

Initial attractor																												Final attractor																										
Perturbation	ATP	Apoptosis	Apoptosome	BAX	BCL2	CASP3	CASP8	Cyt_c	DISC-FAS	DISC-TNF	FASL	IKK	MOMP	MPT	NFkB	NonACD	RIP1	RIP1K	RIP1ub	ROS	SMAC	Survival	TNF	TNFR	XIAP	cFLIP	cIAP		Perturbation	ATP	Apoptosis	Apoptosome	BAX	BCL2	CASP3	CASP8	Cyt_c	DISC-FAS	DISC-TNF	FASL	IKK	MOMP	MPT	NFkB	NonACD	RIP1	RIP1K	RIP1ub	ROS	SMAC	Survival	TNF	TNFR	XIAP	cFLIP	cIAP
Unperturbed	0	0	0	0	0	0	0	1	0	0	0	0	1	1	0	1	0	0	0	1	1	0	0	0	0	0	0	⟶	KO Survival	0	0	0	0	0	0	0	1	0	0	0	0	1	1	0	1	0	0	0	1	1	0	0	0	0	0	0
Unperturbed	0	0	0	0	0	0	0	1	0	0	0	0	1	1	0	1	0	0	0	1	1	0	0	0	0	0	0	⟶	KO TNF	0	0	0	0	0	0	0	1	0	0	0	0	1	1	0	1	0	0	0	1	1	0	0	0	0	0	0
Unperturbed	0	0	0	0	0	0	0	1	0	0	0	0	1	1	0	1	0	0	0	1	1	0	0	0	0	0	0	⟶	KO TNFR	0	0	0	0	0	0	0	1	0	0	0	0	1	1	0	1	0	0	0	1	1	0	0	0	0	0	0
Unperturbed	0	0	0	0	0	0	0	1	0	0	0	0	1	1	0	1	0	0	0	1	1	0	0	0	0	0	0	⟶	KO XIAP	0	0	0	0	0	0	0	1	0	0	0	0	1	1	0	1	0	0	0	1	1	0	0	0	0	0	0
Unperturbed	0	0	0	0	0	0	0	1	0	0	0	0	1	1	0	1	0	0	0	1	1	0	0	0	0	0	0	⟶	KO cFLIP	0	0	0	0	0	0	0	1	0	0	0	0	1	1	0	1	0	0	0	1	1	0	0	0	0	0	0
Unperturbed	0	0	0	0	0	0	0	1	0	0	0	0	1	1	0	1	0	0	0	1	1	0	0	0	0	0	0	⟶	KO cIAP	0	0	0	0	0	0	0	1	0	0	0	0	1	1	0	1	0	0	0	1	1	0	0	0	0	0	0
