# Supplementary material for: Boolean regulatory network reconstruction using literature based knowledge with a genetic algorithm optimization method
Source: BMC Bioinformatics. 2016 Oct 6;17:410. doi: 10.1186/s12859-016-1287-z (PMC5053080; doi:10.1186/s12859-016-1287-z)
Supplement: Additional file 4: — Cell-fate decision model: in silico PKNs. In silico PKNs for the cell-fate decision model. For each PKN, a graphical representation as well as the list of interactions is given. Interactions that were changed to introduce noise in the PKNs are shown in blue in the list of interactions. In the graphical representation, interactions that were changed to introduce noise in the PKNs are shown in magenta (negative interactions) and cyan (positive interactions). (PDF 314 kb) [file 12859_2016_1287_MOESM4_ESM.pdf]

Cell-fate decision model: in-silico PKN 1

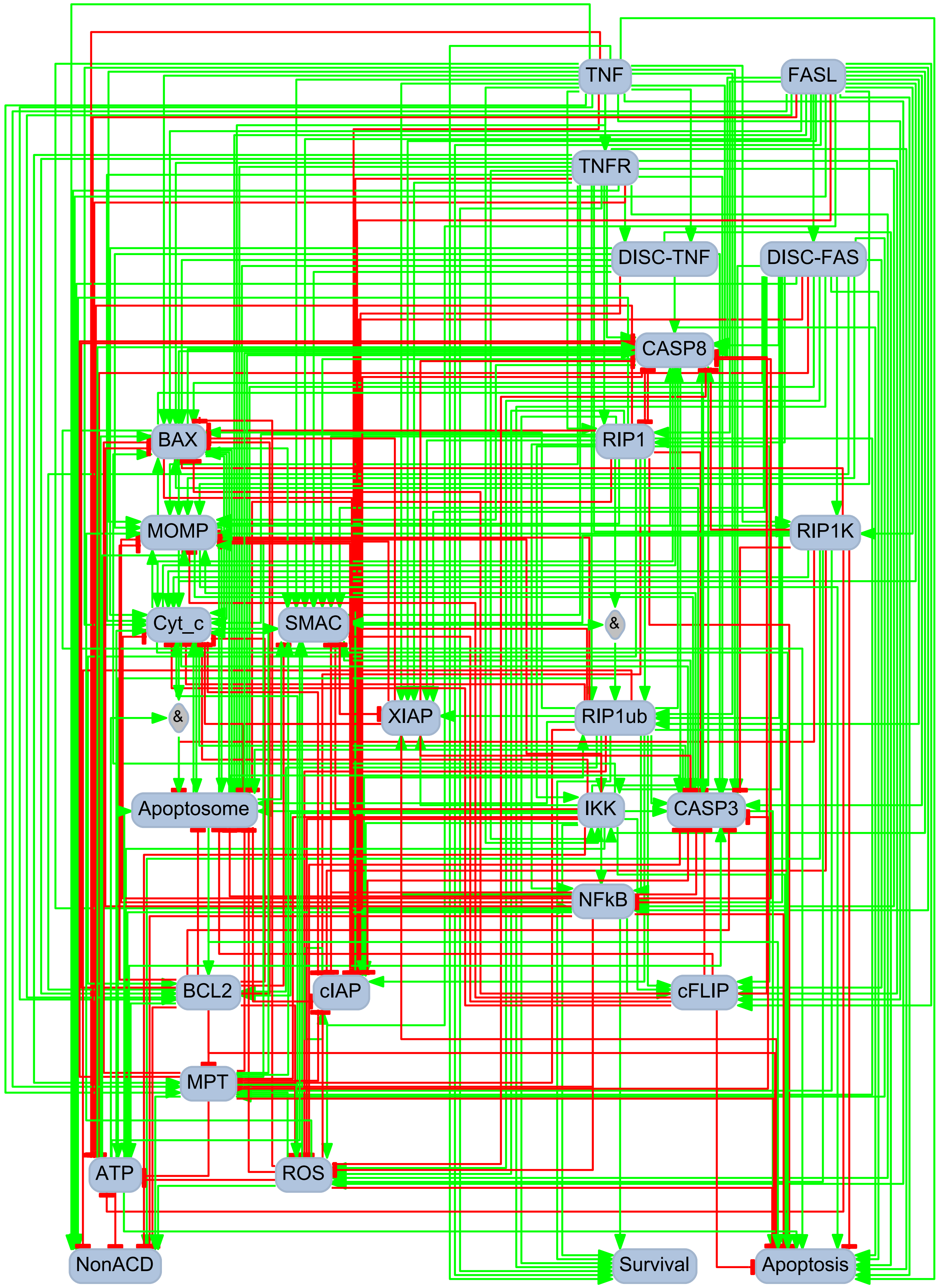

BCL2	$\rightarrow$	ATP
DISC-FAS	$\dashv$	ATP
FASL	$\dashv$	ATP
IKK	$\rightarrow$	ATP
MPT	$\dashv$	ATP
NFkB	$\rightarrow$	ATP
RIP1	$\dashv$	ATP
RIP1K	$\dashv$	ATP
RIP1ub	$\rightarrow$	ATP
ROS	$\dashv$	ATP
TNF	$\dashv$	ATP
TNFR	$\dashv$	ATP
ATP	$\rightarrow$	Apoptosis
Apoptosome	$\rightarrow$	Apoptosis
BAX	$\rightarrow$	Apoptosis
BCL2	$\dashv$	Apoptosis
CASP3	$\rightarrow$	Apoptosis
CASP8	$\rightarrow$	Apoptosis
Cyt_c	$\rightarrow$	Apoptosis
DISC-FAS	$\rightarrow$	Apoptosis
DISC-TNF	$\rightarrow$	Apoptosis
FASL	$\rightarrow$	Apoptosis
IKK	$\rightarrow$	Apoptosis
MOMP	$\rightarrow$	Apoptosis
MPT	$\dashv$	Apoptosis
NFkB	$\dashv$	Apoptosis
RIP1	$\dashv$	Apoptosis
RIP1K	$\dashv$	Apoptosis
RIP1ub	$\rightarrow$	Apoptosis
ROS	$\dashv$	Apoptosis
TNF	$\rightarrow$	Apoptosis
TNFR	$\rightarrow$	Apoptosis
XIAP	$\dashv$	Apoptosis
cFLIP	$\dashv$	Apoptosis
ATP	$\rightarrow$	Apoptosome
BAX	$\rightarrow$	Apoptosome
BCL2	$\dashv$	Apoptosome
CASP3	$\rightarrow$	Apoptosome
CASP8	$\rightarrow$	Apoptosome
Cyt_c	$\rightarrow$	Apoptosome
Cyt_c&ATP	$\rightarrow$	Apoptosome
DISC-FAS	$\rightarrow$	Apoptosome
DISC-TNF	$\rightarrow$	Apoptosome
FASL	$\rightarrow$	Apoptosome
IKK	$\rightarrow$	Apoptosome
MOMP	$\rightarrow$	Apoptosome
MPT	$\dashv$	Apoptosome
NFkB	$\dashv$	Apoptosome
RIP1	$\dashv$	Apoptosome
RIP1K	$\dashv$	Apoptosome
RIP1ub	$\rightarrow$	Apoptosome
ROS	$\dashv$	Apoptosome
TNF	$\rightarrow$	Apoptosome
TNFR	$\rightarrow$	Apoptosome
XIAP	$\dashv$	Apoptosome
cFLIP	$\dashv$	Apoptosome
ATP	$\rightarrow$	BAX
Apoptosome	$\rightarrow$	BAX
BCL2	$\dashv$	BAX
CASP3	$\rightarrow$	BAX
CASP8	$\rightarrow$	BAX
Cyt_c	$\rightarrow$	BAX
DISC-FAS	$\rightarrow$	BAX
DISC-TNF	$\rightarrow$	BAX
FASL	$\rightarrow$	BAX
IKK	$\rightarrow$	BAX

MOMP	$\rightarrow$	BAX
MPT	$\dashv$	BAX
NFkB	$\dashv$	BAX
RIP1	$\dashv$	BAX
RIP1K	$\dashv$	BAX
RIP1ub	$\rightarrow$	BAX
ROS	$\dashv$	BAX
TNF	$\rightarrow$	BAX
TNFR	$\rightarrow$	BAX
XIAP	$\dashv$	BAX
cFLIP	$\dashv$	BAX
DISC-FAS	$\rightarrow$	BCL2
FASL	$\rightarrow$	BCL2
IKK	$\rightarrow$	BCL2
NFkB	$\rightarrow$	BCL2
RIP1	$\rightarrow$	BCL2
RIP1ub	$\rightarrow$	BCL2
TNF	$\rightarrow$	BCL2
TNFR	$\rightarrow$	BCL2
ATP	$\rightarrow$	CASP3
Apoptosome	$\rightarrow$	CASP3
BAX	$\rightarrow$	CASP3
BCL2	$\dashv$	CASP3
CASP8	$\rightarrow$	CASP3
Cyt_c	$\rightarrow$	CASP3
DISC-FAS	$\rightarrow$	CASP3
DISC-TNF	$\rightarrow$	CASP3
FASL	$\rightarrow$	CASP3
IKK	$\rightarrow$	CASP3
MOMP	$\rightarrow$	CASP3
MPT	$\dashv$	CASP3
NFkB	$\dashv$	CASP3
RIP1	$\dashv$	CASP3
RIP1K	$\dashv$	CASP3
RIP1ub	$\rightarrow$	CASP3
ROS	$\dashv$	CASP3
TNF	$\rightarrow$	CASP3
TNFR	$\rightarrow$	CASP3
XIAP	$\dashv$	CASP3
cFLIP	$\dashv$	CASP3
ATP	$\rightarrow$	CASP8
Apoptosome	$\rightarrow$	CASP8
BAX	$\rightarrow$	CASP8
BCL2	$\dashv$	CASP8
CASP3	$\rightarrow$	CASP8
Cyt_c	$\rightarrow$	CASP8
DISC-FAS	$\rightarrow$	CASP8
DISC-TNF	$\rightarrow$	CASP8
FASL	$\rightarrow$	CASP8
IKK	$\rightarrow$	CASP8
MOMP	$\rightarrow$	CASP8
MPT	$\dashv$	CASP8
NFkB	$\dashv$	CASP8
RIP1	$\dashv$	CASP8
RIP1K	$\dashv$	CASP8
RIP1ub	$\rightarrow$	CASP8
ROS	$\dashv$	CASP8
TNF	$\rightarrow$	CASP8
TNFR	$\rightarrow$	CASP8
XIAP	$\dashv$	CASP8
cFLIP	$\dashv$	CASP8
ATP	$\rightarrow$	Cyt_c
Apoptosome	$\rightarrow$	Cyt_c
BAX	$\rightarrow$	Cyt_c
BCL2	$\dashv$	Cyt_c
CASP3	$\rightarrow$	Cyt_c

CASP8	$\rightarrow$	Cyt_c
DISC-FAS	$\rightarrow$	Cyt_c
DISC-TNF	$\rightarrow$	Cyt_c
FASL	$\rightarrow$	Cyt_c
IKK	$\dashv$	Cyt_c
MOMP	$\rightarrow$	Cyt_c
MPT	$\rightarrow$	Cyt_c
NFkB	$\dashv$	Cyt_c
RIP1	$\rightarrow$	Cyt_c
RIP1K	$\rightarrow$	Cyt_c
RIP1ub	$\dashv$	Cyt_c
ROS	$\rightarrow$	Cyt_c
TNF	$\rightarrow$	Cyt_c
TNFR	$\rightarrow$	Cyt_c
XIAP	$\dashv$	Cyt_c
cFLIP	$\dashv$	Cyt_c
FASL	$\rightarrow$	DISC-FAS
TNF	$\rightarrow$	DISC-TNF
TNFR	$\rightarrow$	DISC-TNF
DISC-FAS	$\rightarrow$	IKK
FASL	$\rightarrow$	IKK
RIP1	$\rightarrow$	IKK
RIP1ub	$\rightarrow$	IKK
TNF	$\rightarrow$	IKK
TNFR	$\rightarrow$	IKK
ATP	$\rightarrow$	MOMP
Apoptosome	$\rightarrow$	MOMP
BAX	$\rightarrow$	MOMP
BCL2	$\dashv$	MOMP
CASP3	$\rightarrow$	MOMP
CASP8	$\rightarrow$	MOMP
Cyt_c	$\rightarrow$	MOMP
DISC-FAS	$\rightarrow$	MOMP
DISC-TNF	$\rightarrow$	MOMP
FASL	$\rightarrow$	MOMP
IKK	$\dashv$	MOMP
MPT	$\rightarrow$	MOMP
NFkB	$\dashv$	MOMP
RIP1	$\rightarrow$	MOMP
RIP1K	$\rightarrow$	MOMP
RIP1ub	$\dashv$	MOMP
ROS	$\rightarrow$	MOMP
TNF	$\rightarrow$	MOMP
TNFR	$\rightarrow$	MOMP
XIAP	$\dashv$	MOMP
cFLIP	$\dashv$	MOMP
BCL2	$\dashv$	MPT
DISC-FAS	$\rightarrow$	MPT
FASL	$\rightarrow$	MPT
IKK	$\dashv$	MPT
NFkB	$\dashv$	MPT
RIP1	$\rightarrow$	MPT
RIP1K	$\rightarrow$	MPT
RIP1ub	$\dashv$	MPT
ROS	$\rightarrow$	MPT
TNF	$\rightarrow$	MPT
TNFR	$\rightarrow$	MPT
CASP3	$\dashv$	NFkB
DISC-FAS	$\rightarrow$	NFkB
FASL	$\rightarrow$	NFkB
IKK	$\rightarrow$	NFkB
RIP1	$\rightarrow$	NFkB
RIP1ub	$\rightarrow$	NFkB
TNF	$\rightarrow$	NFkB
TNFR	$\rightarrow$	NFkB
ATP	$\dashv$	NonACD

BCL2	⊣	NonACD
DISC-FAS	→	NonACD
FASL	→	NonACD
IKK	⊣	NonACD
MPT	→	NonACD
NFkB	⊣	NonACD
RIP1	→	NonACD
RIP1K	→	NonACD
RIP1ub	⊣	NonACD
ROS	→	NonACD
TNF	→	NonACD
TNFR	→	NonACD
CASP8	⊣	RIP1
DISC-FAS	→	RIP1
FASL	→	RIP1
TNF	→	RIP1
TNFR	→	RIP1
DISC-FAS	→	RIP1K
FASL	→	RIP1K
RIP1	→	RIP1K
TNF	→	RIP1K
TNFR	→	RIP1K
DISC-FAS	→	RIP1ub
FASL	→	RIP1ub
RIP1	→	RIP1ub
TNF	→	RIP1ub
TNFR	→	RIP1ub
cIAP	→	RIP1ub
cIAP&RIP1	→	RIP1ub
BCL2	⊣	ROS
DISC-FAS	→	ROS
FASL	→	ROS
IKK	⊣	ROS
MPT	→	ROS
NFkB	⊣	ROS
RIP1	→	ROS
RIP1K	→	ROS
RIP1ub	⊣	ROS
TNF	→	ROS
TNFR	→	ROS
ATP	→	SMAC
Apoptosome	→	SMAC
BAX	→	SMAC
BCL2	⊣	SMAC
CASP3	→	SMAC
CASP8	→	SMAC
Cyt_c	→	SMAC
DISC-FAS	→	SMAC
DISC-TNF	→	SMAC
FASL	→	SMAC
IKK	⊣	SMAC
MOMP	→	SMAC
MPT	→	SMAC
NFkB	⊣	SMAC
RIP1	→	SMAC
RIP1K	→	SMAC
RIP1ub	⊣	SMAC
ROS	→	SMAC
TNF	→	SMAC
TNFR	→	SMAC
XIAP	⊣	SMAC
cFLIP	⊣	SMAC
DISC-FAS	→	Survival
FASL	→	Survival
IKK	→	Survival
NFkB	→	Survival

RIP1	$\rightarrow$	Survival
RIP1ub	$\rightarrow$	Survival
TNF	$\rightarrow$	Survival
TNFR	$\rightarrow$	Survival
TNF	$\rightarrow$	TNFR
DISC-FAS	$\rightarrow$	XIAP
FASL	$\rightarrow$	XIAP
IKK	$\rightarrow$	XIAP
NFkB	$\rightarrow$	XIAP
RIP1	$\rightarrow$	XIAP
RIP1ub	$\rightarrow$	XIAP
SMAC	$\dashv$	XIAP
TNF	$\rightarrow$	XIAP
TNFR	$\rightarrow$	XIAP
DISC-FAS	$\rightarrow$	cFLIP
FASL	$\rightarrow$	cFLIP
IKK	$\rightarrow$	cFLIP
NFkB	$\rightarrow$	cFLIP
RIP1	$\rightarrow$	cFLIP
RIP1ub	$\rightarrow$	cFLIP
TNF	$\rightarrow$	cFLIP
TNFR	$\rightarrow$	cFLIP
Apoptosome	$\dashv$	cIAP
BAX	$\dashv$	cIAP
CASP3	$\dashv$	cIAP
CASP8	$\dashv$	cIAP
Cyt_c	$\dashv$	cIAP
DISC-FAS	$\dashv$	cIAP
DISC-TNF	$\dashv$	cIAP
FASL	$\dashv$	cIAP
IKK	$\rightarrow$	cIAP
MOMP	$\dashv$	cIAP
MPT	$\dashv$	cIAP
NFkB	$\rightarrow$	cIAP
RIP1	$\dashv$	cIAP
RIP1K	$\dashv$	cIAP
RIP1ub	$\rightarrow$	cIAP
ROS	$\dashv$	cIAP
SMAC	$\dashv$	cIAP
TNF	$\dashv$	cIAP
TNFR	$\dashv$	cIAP
cIAP	$\rightarrow$	cIAP

Cell-fate decision model: in-silico PKN 2 (10% noise)

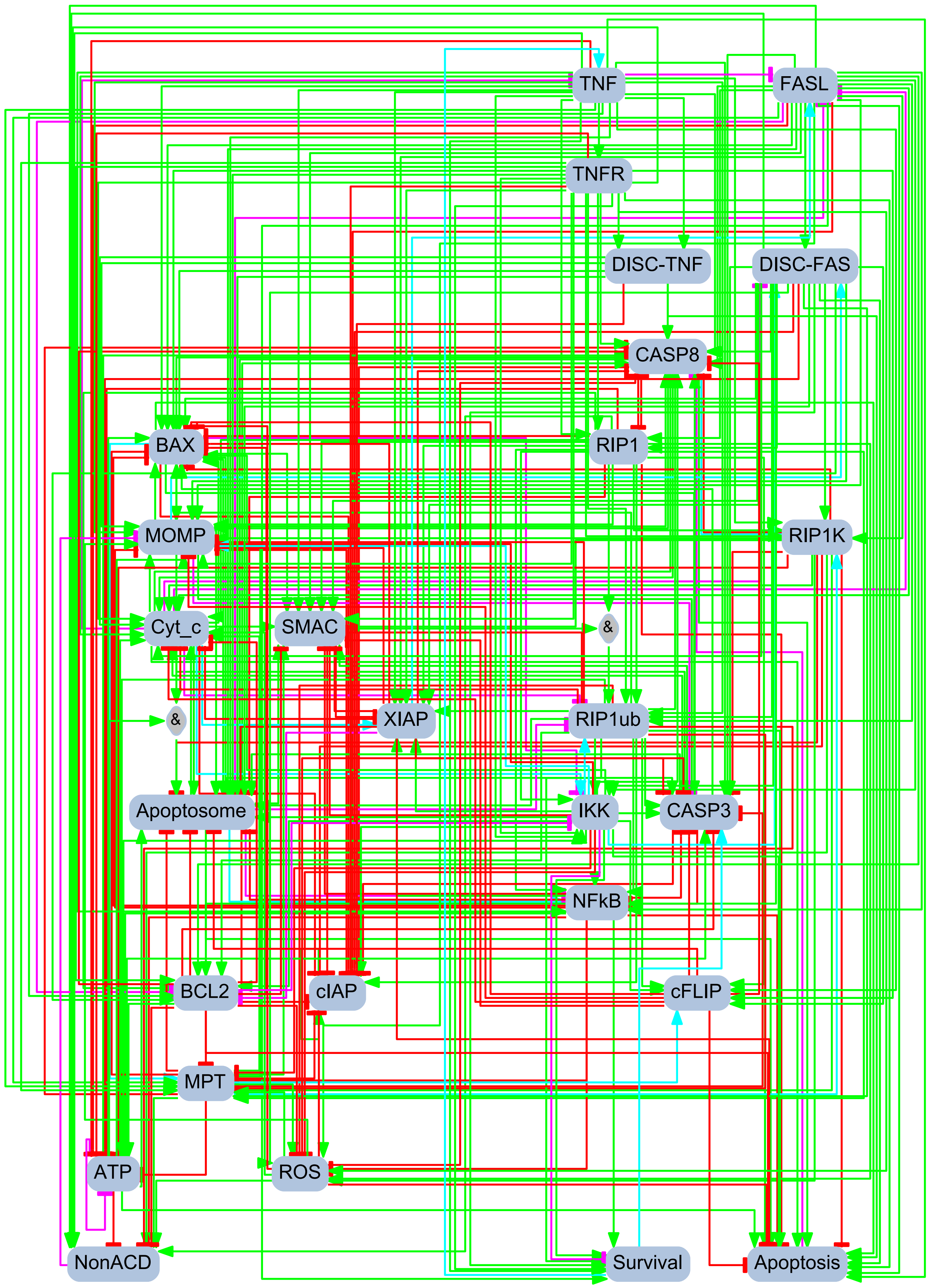

BCL2	→	ATP
DISC-FAS	⊣	ATP
FASL	⊣	ATP
IKK	→	ATP
MPT	⊣	ATP
NFkB	→	ATP
RIP1	⊣	ATP
RIP1K	⊣	ATP
RIP1ub	→	ATP
TNF	⊣	ATP
TNFR	⊣	ATP
ATP	⊣	ATP
ATP	→	Apoptosis
Apoptosome	→	Apoptosis
BAX	→	Apoptosis
BCL2	⊣	Apoptosis
CASP3	→	Apoptosis
CASP8	→	Apoptosis
Cyt_c	→	Apoptosis
DISC-FAS	→	Apoptosis
DISC-TNF	→	Apoptosis
FASL	→	Apoptosis
IKK	→	Apoptosis
NFkB	⊣	Apoptosis
RIP1	⊣	Apoptosis
RIP1K	⊣	Apoptosis
RIP1ub	→	Apoptosis
ROS	⊣	Apoptosis
TNF	→	Apoptosis
TNFR	→	Apoptosis
XIAP	⊣	Apoptosis
cFLIP	⊣	Apoptosis
ATP	→	Apoptosome
BAX	→	Apoptosome
BCL2	⊣	Apoptosome
CASP3	→	Apoptosome
CASP8	→	Apoptosome
Cyt_c	→	Apoptosome
Cyt_c&ATP	→	Apoptosome
DISC-FAS	→	Apoptosome
DISC-TNF	→	Apoptosome
FASL	→	Apoptosome
IKK	→	Apoptosome
MOMP	→	Apoptosome
MPT	⊣	Apoptosome
NFkB	⊣	Apoptosome
RIP1	⊣	Apoptosome
RIP1K	⊣	Apoptosome
RIP1ub	→	Apoptosome
TNF	→	Apoptosome
TNFR	→	Apoptosome
XIAP	⊣	Apoptosome
cFLIP	⊣	Apoptosome
ATP	→	BAX
Apoptosome	→	BAX
BCL2	⊣	BAX
CASP3	→	BAX
CASP8	→	BAX
Cyt_c	→	BAX
DISC-FAS	→	BAX
DISC-TNF	→	BAX
FASL	→	BAX
IKK	→	BAX
MOMP	→	BAX
MPT	⊣	BAX
NFkB	⊣	BAX

RIP1	⊣	BAX
RIP1K	⊣	BAX
RIP1ub	→	BAX
ROS	⊣	BAX
TNF	→	BAX
TNFR	→	BAX
XIAP	⊣	BAX
cFLIP	⊣	BAX
DISC-FAS	→	BCL2
FASL	→	BCL2
IKK	→	BCL2
NFkB	→	BCL2
RIP1	→	BCL2
RIP1ub	→	BCL2
TNF	→	BCL2
TNFR	→	BCL2
FASL	⊣	BCL2
XIAP	⊣	BCL2
ATP	→	CASP3
Apoptosome	→	CASP3
BAX	→	CASP3
BCL2	⊣	CASP3
Cyt_c	→	CASP3
DISC-FAS	→	CASP3
FASL	→	CASP3
IKK	→	CASP3
MOMP	→	CASP3
MPT	⊣	CASP3
NFkB	⊣	CASP3
RIP1K	⊣	CASP3
RIP1ub	→	CASP3
ROS	⊣	CASP3
TNF	→	CASP3
XIAP	⊣	CASP3
cFLIP	⊣	CASP3
MOMP	⊣	CASP3
Survival	→	CASP3
ATP	→	CASP8
Apoptosome	→	CASP8
BCL2	⊣	CASP8
CASP3	→	CASP8
Cyt_c	→	CASP8
DISC-FAS	→	CASP8
DISC-TNF	→	CASP8
FASL	→	CASP8
IKK	→	CASP8
MOMP	→	CASP8
MPT	⊣	CASP8
RIP1	⊣	CASP8
RIP1K	⊣	CASP8
RIP1ub	→	CASP8
ROS	⊣	CASP8
TNF	→	CASP8
TNFR	→	CASP8
XIAP	⊣	CASP8
cFLIP	⊣	CASP8
Apoptosis	⊣	CASP8
ATP	→	Cyt_c
Apoptosome	→	Cyt_c
BCL2	⊣	Cyt_c
CASP3	→	Cyt_c
CASP8	→	Cyt_c
DISC-FAS	→	Cyt_c
DISC-TNF	→	Cyt_c
FASL	→	Cyt_c
MOMP	→	Cyt_c

MPT	→	Cyt_c
RIP1	→	Cyt_c
RIP1K	→	Cyt_c
RIP1ub	⊣	Cyt_c
ROS	→	Cyt_c
TNF	→	Cyt_c
TNFR	→	Cyt_c
XIAP	⊣	Cyt_c
cFLIP	⊣	Cyt_c
FASL	→	DISC-FAS
Cyt_c	⊣	DISC-FAS
IKK	→	DISC-FAS
MOMP	→	DISC-FAS
TNF	→	DISC-TNF
TNFR	→	DISC-TNF
Apoptosome	⊣	FASL
Cyt_c	⊣	FASL
TNF	⊣	FASL
XIAP	→	FASL
DISC-FAS	→	IKK
FASL	→	IKK
RIP1	→	IKK
RIP1ub	→	IKK
TNF	→	IKK
TNFR	→	IKK
BAX	⊣	IKK
BCL2	⊣	IKK
Cyt_c	→	IKK
ATP	→	MOMP
BAX	→	MOMP
CASP8	→	MOMP
Cyt_c	→	MOMP
DISC-FAS	→	MOMP
DISC-TNF	→	MOMP
FASL	→	MOMP
IKK	⊣	MOMP
MPT	→	MOMP
NFkB	⊣	MOMP
RIP1	→	MOMP
RIP1K	→	MOMP
RIP1ub	⊣	MOMP
ROS	→	MOMP
TNF	→	MOMP
TNFR	→	MOMP
XIAP	⊣	MOMP
cFLIP	⊣	MOMP
IKK	→	MOMP
NonACD	⊣	MOMP
BCL2	⊣	MPT
DISC-FAS	→	MPT
FASL	→	MPT
IKK	⊣	MPT
NFkB	⊣	MPT
RIP1	→	MPT
RIP1K	→	MPT
RIP1ub	⊣	MPT
ROS	→	MPT
TNF	→	MPT
TNFR	→	MPT
BAX	→	MPT
CASP3	⊣	NFkB
DISC-FAS	→	NFkB
FASL	→	NFkB
IKK	→	NFkB
RIP1	→	NFkB
RIP1ub	→	NFkB

TNF	→	NFkB
Apoptosome	→	NFkB
Apoptosome	⊣	NFkB
ATP	⊣	NonACD
BCL2	⊣	NonACD
DISC-FAS	→	NonACD
FASL	→	NonACD
MPT	→	NonACD
NFkB	⊣	NonACD
RIP1	→	NonACD
RIP1K	→	NonACD
RIP1ub	⊣	NonACD
ROS	→	NonACD
TNF	→	NonACD
TNFR	→	NonACD
CASP8	⊣	RIP1
DISC-FAS	→	RIP1
FASL	→	RIP1
TNF	→	RIP1
TNFR	→	RIP1
DISC-FAS	→	RIP1K
FASL	→	RIP1K
RIP1	→	RIP1K
TNF	→	RIP1K
TNFR	→	RIP1K
CASP8	→	RIP1K
MPT	→	RIP1K
DISC-FAS	→	RIP1ub
FASL	→	RIP1ub
RIP1	→	RIP1ub
TNF	→	RIP1ub
TNFR	→	RIP1ub
cIAP&RIP1	→	RIP1ub
Apoptosome	⊣	RIP1ub
Cyt_c	⊣	RIP1ub
IKK	→	RIP1ub
BCL2	⊣	ROS
DISC-FAS	→	ROS
FASL	→	ROS
IKK	⊣	ROS
MPT	→	ROS
NFkB	⊣	ROS
RIP1	→	ROS
RIP1K	→	ROS
RIP1ub	⊣	ROS
TNFR	→	ROS
Apoptosome	→	SMAC
BAX	→	SMAC
BCL2	⊣	SMAC
CASP3	→	SMAC
Cyt_c	→	SMAC
DISC-FAS	→	SMAC
FASL	→	SMAC
IKK	⊣	SMAC
MPT	→	SMAC
NFkB	⊣	SMAC
RIP1	→	SMAC
RIP1K	→	SMAC
RIP1ub	⊣	SMAC
TNF	→	SMAC
TNFR	→	SMAC
XIAP	⊣	SMAC
cFLIP	⊣	SMAC
DISC-FAS	→	Survival
FASL	→	Survival
IKK	→	Survival

NFkB	→	Survival
RIP1ub	→	Survival
TNF	→	Survival
TNFR	→	Survival
IKK	⊣	Survival
Cyt_c	⊣	TNF
Survival	→	TNF
TNF	→	TNFR
DISC-FAS	→	XIAP
FASL	→	XIAP
IKK	→	XIAP
NFkB	→	XIAP
RIP1	→	XIAP
RIP1ub	→	XIAP
SMAC	⊣	XIAP
TNF	→	XIAP
TNFR	→	XIAP
Cyt_c	→	XIAP
DISC-FAS	→	cFLIP
IKK	→	cFLIP
RIP1	→	cFLIP
RIP1ub	→	cFLIP
TNF	→	cFLIP
TNFR	→	cFLIP
MPT	→	cFLIP
Apoptosome	⊣	cIAP
BAX	⊣	cIAP
CASP3	⊣	cIAP
CASP8	⊣	cIAP
Cyt_c	⊣	cIAP
DISC-FAS	⊣	cIAP
DISC-TNF	⊣	cIAP
FASL	⊣	cIAP
IKK	→	cIAP
MOMP	⊣	cIAP
MPT	⊣	cIAP
RIP1K	⊣	cIAP
RIP1ub	→	cIAP
ROS	⊣	cIAP
SMAC	⊣	cIAP
TNFR	⊣	cIAP
cIAP	→	cIAP

Cell-fate decision model: in-silico PKN 3 (20% noise)

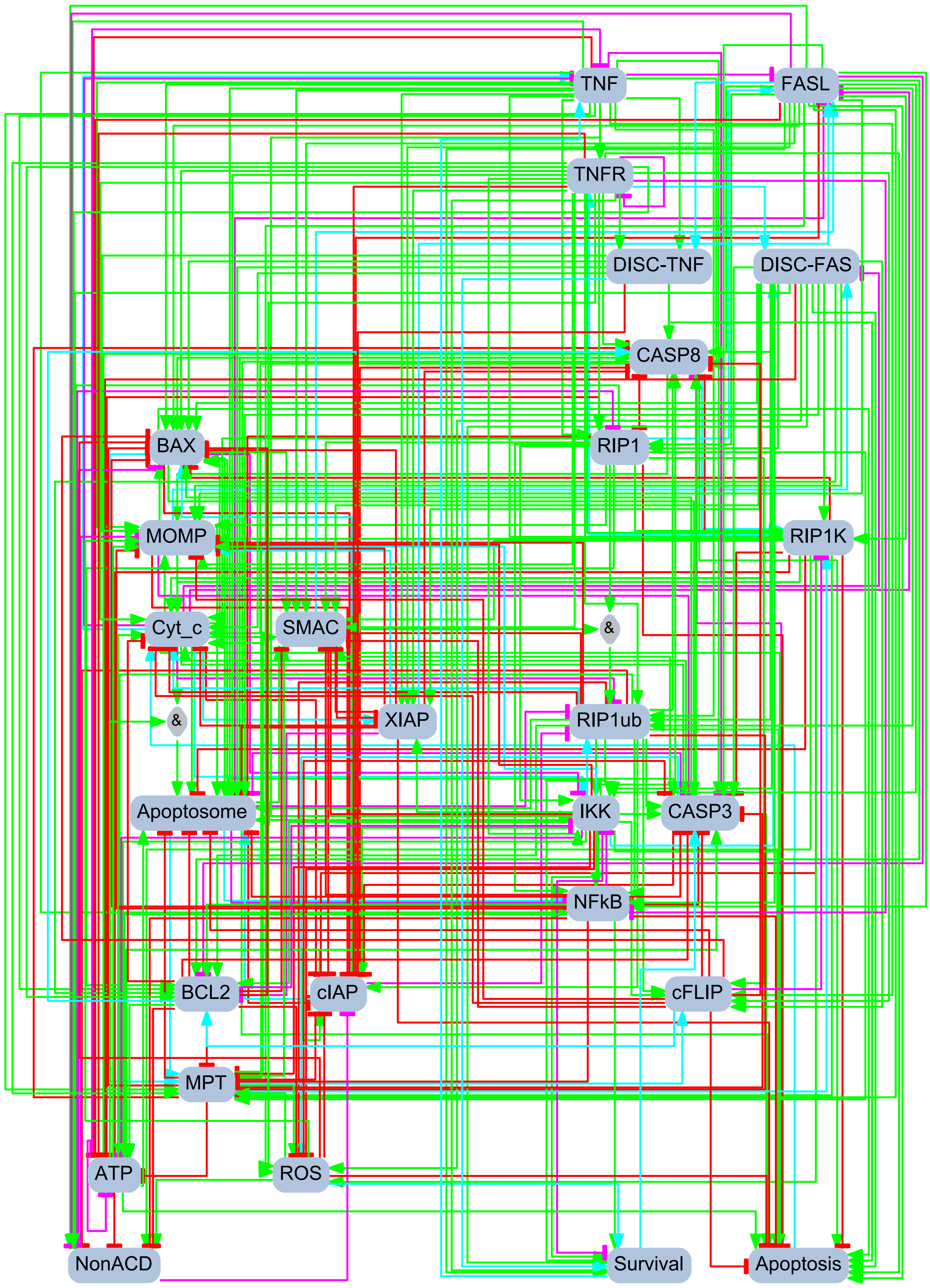

BCL2	$\to$	ATP
DISC-FAS	$\dashv$	ATP
FASL	$\dashv$	ATP
IKK	$\to$	ATP
MPT	$\dashv$	ATP
NFkB	$\to$	ATP
RIP1	$\dashv$	ATP
RIP1K	$\dashv$	ATP
RIP1ub	$\to$	ATP
TNF	$\dashv$	ATP
TNFR	$\dashv$	ATP
ATP	$\dashv$	ATP
IKK	$\dashv$	ATP
ATP	$\to$	Apoptosis
Apoptosome	$\to$	Apoptosis
BAX	$\to$	Apoptosis
CASP3	$\to$	Apoptosis
CASP8	$\to$	Apoptosis
Cyt_c	$\to$	Apoptosis
DISC-FAS	$\to$	Apoptosis
DISC-TNF	$\to$	Apoptosis
FASL	$\to$	Apoptosis
IKK	$\to$	Apoptosis
NFkB	$\dashv$	Apoptosis
RIP1	$\dashv$	Apoptosis
RIP1K	$\dashv$	Apoptosis
RIP1ub	$\to$	Apoptosis
ROS	$\dashv$	Apoptosis
TNFR	$\to$	Apoptosis
XIAP	$\dashv$	Apoptosis
cFLIP	$\dashv$	Apoptosis
ATP	$\to$	Apoptosome
BAX	$\to$	Apoptosome
BCL2	$\dashv$	Apoptosome
CASP8	$\to$	Apoptosome
Cyt_c	$\to$	Apoptosome
Cyt_c&ATP	$\to$	Apoptosome
DISC-FAS	$\to$	Apoptosome
DISC-TNF	$\to$	Apoptosome
IKK	$\to$	Apoptosome
MOMP	$\to$	Apoptosome
MPT	$\dashv$	Apoptosome
NFkB	$\dashv$	Apoptosome
RIP1	$\dashv$	Apoptosome
RIP1K	$\dashv$	Apoptosome
RIP1ub	$\to$	Apoptosome
TNF	$\to$	Apoptosome
TNFR	$\to$	Apoptosome
XIAP	$\dashv$	Apoptosome
cFLIP	$\dashv$	Apoptosome
CASP3	$\dashv$	Apoptosome
cIAP	$\to$	Apoptosome
Apoptosome	$\to$	BAX
BCL2	$\dashv$	BAX
CASP3	$\to$	BAX
CASP8	$\to$	BAX
Cyt_c	$\to$	BAX
DISC-FAS	$\to$	BAX
DISC-TNF	$\to$	BAX
FASL	$\to$	BAX
MOMP	$\to$	BAX
MPT	$\dashv$	BAX
NFkB	$\dashv$	BAX
RIP1K	$\dashv$	BAX
ROS	$\dashv$	BAX
TNF	$\to$	BAX

TNFR	→	BAX
XIAP	⊣	BAX
cFLIP	⊣	BAX
NonACD	⊣	BAX
cIAP	→	BAX
DISC-FAS	→	BCL2
FASL	→	BCL2
IKK	→	BCL2
NFkB	→	BCL2
RIP1	→	BCL2
RIP1ub	→	BCL2
TNF	→	BCL2
TNFR	→	BCL2
FASL	⊣	BCL2
XIAP	⊣	BCL2
cFLIP	→	BCL2
ATP	→	CASP3
Apoptosome	→	CASP3
BAX	→	CASP3
BCL2	⊣	CASP3
Cyt_c	→	CASP3
DISC-FAS	→	CASP3
FASL	→	CASP3
IKK	→	CASP3
MPT	⊣	CASP3
NFkB	⊣	CASP3
RIP1K	⊣	CASP3
RIP1ub	→	CASP3
ROS	⊣	CASP3
TNF	→	CASP3
cFLIP	⊣	CASP3
MOMP	⊣	CASP3
Survival	→	CASP3
TNF	⊣	CASP3
ATP	→	CASP8
Apoptosome	→	CASP8
CASP3	→	CASP8
DISC-FAS	→	CASP8
DISC-TNF	→	CASP8
FASL	→	CASP8
IKK	→	CASP8
MPT	⊣	CASP8
RIP1	⊣	CASP8
RIP1K	⊣	CASP8
RIP1ub	→	CASP8
TNF	→	CASP8
TNFR	→	CASP8
XIAP	⊣	CASP8
cFLIP	⊣	CASP8
Apoptosis	⊣	CASP8
BCL2	→	CASP8
ATP	→	Cyt_c
BCL2	⊣	Cyt_c
CASP3	→	Cyt_c
CASP8	→	Cyt_c
DISC-FAS	→	Cyt_c
DISC-TNF	→	Cyt_c
MOMP	→	Cyt_c
MPT	→	Cyt_c
RIP1	→	Cyt_c
RIP1K	→	Cyt_c
RIP1ub	⊣	Cyt_c
TNFR	→	Cyt_c
XIAP	⊣	Cyt_c
cFLIP	⊣	Cyt_c
Apoptosis	→	Cyt_c

RIP1ub	$\rightarrow$	Cyt_c
Cyt_c	$\dashv$	DISC-FAS
IKK	$\rightarrow$	DISC-FAS
MOMP	$\rightarrow$	DISC-FAS
TNFR	$\rightarrow$	DISC-FAS
TNF	$\rightarrow$	DISC-TNF
TNFR	$\rightarrow$	DISC-TNF
FASL	$\rightarrow$	DISC-TNF
Apoptosome	$\dashv$	FASL
Cyt_c	$\dashv$	FASL
RIP1	$\rightarrow$	FASL
SMAC	$\rightarrow$	FASL
TNF	$\dashv$	FASL
XIAP	$\rightarrow$	FASL
DISC-FAS	$\rightarrow$	IKK
FASL	$\rightarrow$	IKK
RIP1	$\rightarrow$	IKK
RIP1ub	$\rightarrow$	IKK
TNF	$\rightarrow$	IKK
TNFR	$\rightarrow$	IKK
BAX	$\dashv$	IKK
BCL2	$\dashv$	IKK
Cyt_c	$\rightarrow$	IKK
NFkB	$\dashv$	IKK
ATP	$\rightarrow$	MOMP
CASP8	$\rightarrow$	MOMP
Cyt_c	$\rightarrow$	MOMP
DISC-FAS	$\rightarrow$	MOMP
DISC-TNF	$\rightarrow$	MOMP
FASL	$\rightarrow$	MOMP
IKK	$\dashv$	MOMP
MPT	$\rightarrow$	MOMP
NFkB	$\dashv$	MOMP
RIP1	$\rightarrow$	MOMP
RIP1K	$\rightarrow$	MOMP
RIP1ub	$\dashv$	MOMP
ROS	$\rightarrow$	MOMP
TNF	$\rightarrow$	MOMP
TNFR	$\rightarrow$	MOMP
XIAP	$\dashv$	MOMP
cFLIP	$\dashv$	MOMP
IKK	$\rightarrow$	MOMP
NonACD	$\dashv$	MOMP
XIAP	$\rightarrow$	MOMP
BCL2	$\dashv$	MPT
FASL	$\rightarrow$	MPT
IKK	$\dashv$	MPT
NFkB	$\dashv$	MPT
RIP1	$\rightarrow$	MPT
RIP1K	$\rightarrow$	MPT
RIP1ub	$\dashv$	MPT
ROS	$\rightarrow$	MPT
TNF	$\rightarrow$	MPT
TNFR	$\rightarrow$	MPT
Apoptosome	$\rightarrow$	MPT
BAX	$\rightarrow$	MPT
CASP3	$\dashv$	NFkB
DISC-FAS	$\rightarrow$	NFkB
FASL	$\rightarrow$	NFkB
IKK	$\rightarrow$	NFkB
RIP1	$\rightarrow$	NFkB
RIP1ub	$\rightarrow$	NFkB
TNF	$\rightarrow$	NFkB
Apoptosome	$\rightarrow$	NFkB
Apoptosome	$\dashv$	NFkB
TNFR	$\dashv$	NFkB

ATP	$\dashv$	NonACD
BCL2	$\dashv$	NonACD
DISC-FAS	$\rightarrow$	NonACD
FASL	$\rightarrow$	NonACD
NFkB	$\dashv$	NonACD
RIP1	$\rightarrow$	NonACD
RIP1K	$\rightarrow$	NonACD
RIP1ub	$\dashv$	NonACD
ROS	$\rightarrow$	NonACD
TNF	$\rightarrow$	NonACD
TNFR	$\rightarrow$	NonACD
FASL	$\dashv$	NonACD
CASP8	$\dashv$	RIP1
DISC-FAS	$\rightarrow$	RIP1
FASL	$\rightarrow$	RIP1
TNF	$\rightarrow$	RIP1
TNFR	$\rightarrow$	RIP1
NonACD	$\dashv$	RIP1
DISC-FAS	$\rightarrow$	RIP1K
FASL	$\rightarrow$	RIP1K
RIP1	$\rightarrow$	RIP1K
TNF	$\rightarrow$	RIP1K
TNFR	$\rightarrow$	RIP1K
CASP8	$\rightarrow$	RIP1K
MPT	$\rightarrow$	RIP1K
cFLIP	$\dashv$	RIP1K
DISC-FAS	$\rightarrow$	RIP1ub
FASL	$\rightarrow$	RIP1ub
TNF	$\rightarrow$	RIP1ub
TNFR	$\rightarrow$	RIP1ub
cIAP&RIP1	$\rightarrow$	RIP1ub
Apoptosome	$\dashv$	RIP1ub
Cyt_c	$\dashv$	RIP1ub
IKK	$\rightarrow$	RIP1ub
cIAP	$\dashv$	RIP1ub
BCL2	$\dashv$	ROS
DISC-FAS	$\rightarrow$	ROS
FASL	$\rightarrow$	ROS
IKK	$\dashv$	ROS
MPT	$\rightarrow$	ROS
RIP1K	$\rightarrow$	ROS
RIP1ub	$\dashv$	ROS
TNFR	$\rightarrow$	ROS
CASP3	$\rightarrow$	ROS
Apoptosome	$\rightarrow$	SMAC
BAX	$\rightarrow$	SMAC
BCL2	$\dashv$	SMAC
CASP3	$\rightarrow$	SMAC
Cyt_c	$\rightarrow$	SMAC
DISC-FAS	$\rightarrow$	SMAC
FASL	$\rightarrow$	SMAC
IKK	$\dashv$	SMAC
MPT	$\rightarrow$	SMAC
NFkB	$\dashv$	SMAC
RIP1	$\rightarrow$	SMAC
RIP1K	$\rightarrow$	SMAC
RIP1ub	$\dashv$	SMAC
TNF	$\rightarrow$	SMAC
TNFR	$\rightarrow$	SMAC
XIAP	$\dashv$	SMAC
cFLIP	$\dashv$	SMAC
DISC-FAS	$\rightarrow$	Survival
FASL	$\rightarrow$	Survival
IKK	$\rightarrow$	Survival
NFkB	$\rightarrow$	Survival
RIP1ub	$\rightarrow$	Survival

TNFR	$\rightarrow$	Survival
DISC-TNF	$\rightarrow$	Survival
IKK	$\dashv$	Survival
ROS	$\rightarrow$	Survival
ATP	$\dashv$	TNF
Cyt_c	$\rightarrow$	TNF
Cyt_c	$\dashv$	TNF
Survival	$\rightarrow$	TNF
TNF	$\rightarrow$	TNFR
RIP1K	$\rightarrow$	TNFR
TNFR	$\dashv$	TNFR
DISC-FAS	$\rightarrow$	XIAP
FASL	$\rightarrow$	XIAP
IKK	$\rightarrow$	XIAP
SMAC	$\dashv$	XIAP
TNF	$\rightarrow$	XIAP
TNFR	$\rightarrow$	XIAP
Cyt_c	$\rightarrow$	XIAP
DISC-FAS	$\rightarrow$	cFLIP
IKK	$\rightarrow$	cFLIP
RIP1	$\rightarrow$	cFLIP
RIP1ub	$\rightarrow$	cFLIP
TNF	$\rightarrow$	cFLIP
TNFR	$\rightarrow$	cFLIP
MPT	$\rightarrow$	cFLIP
Apoptosome	$\dashv$	cIAP
BAX	$\dashv$	cIAP
CASP3	$\dashv$	cIAP
CASP8	$\dashv$	cIAP
Cyt_c	$\dashv$	cIAP
DISC-TNF	$\dashv$	cIAP
FASL	$\dashv$	cIAP
IKK	$\rightarrow$	cIAP
MOMP	$\dashv$	cIAP
MPT	$\dashv$	cIAP
RIP1K	$\dashv$	cIAP
RIP1ub	$\rightarrow$	cIAP
ROS	$\dashv$	cIAP
SMAC	$\dashv$	cIAP
TNFR	$\dashv$	cIAP
cIAP	$\rightarrow$	cIAP
NonACD	$\dashv$	cIAP

Cell-fate decision model: in-silico PKN 4 (30% noise)

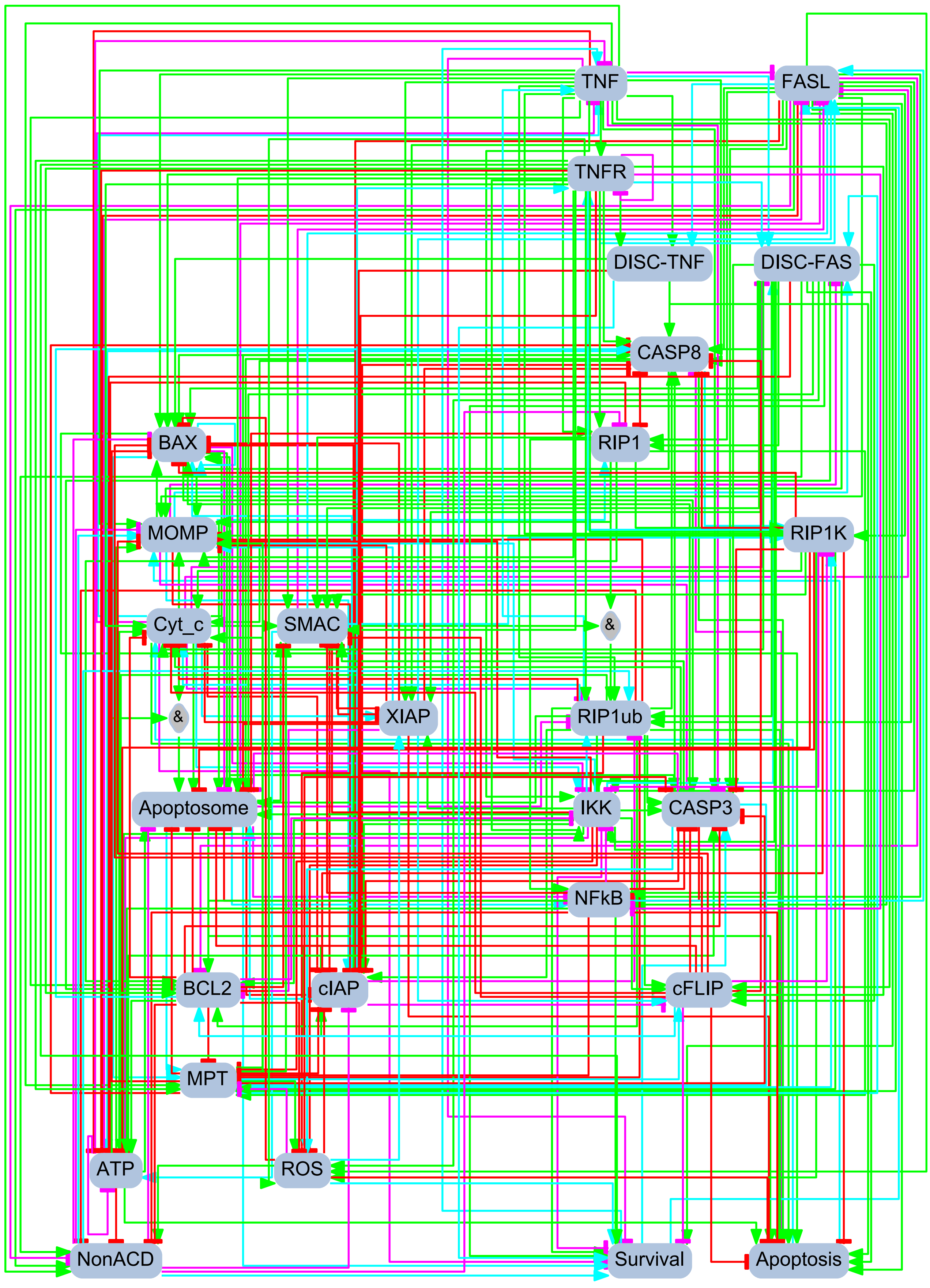

BCL2	→	ATP
DISC-FAS	⊣	ATP
FASL	⊣	ATP
IKK	→	ATP
NFkB	→	ATP
RIP1	⊣	ATP
RIP1K	⊣	ATP
RIP1ub	→	ATP
TNF	⊣	ATP
TNFR	⊣	ATP
ATP	⊣	ATP
CASP8	→	ATP
IKK	⊣	ATP
SMAC	→	ATP
ATP	→	Apoptosis
Apoptosome	→	Apoptosis
BAX	→	Apoptosis
CASP8	→	Apoptosis
Cyt_c	→	Apoptosis
DISC-FAS	→	Apoptosis
DISC-TNF	→	Apoptosis
FASL	→	Apoptosis
IKK	→	Apoptosis
NFkB	⊣	Apoptosis
RIP1K	⊣	Apoptosis
RIP1ub	→	Apoptosis
ROS	⊣	Apoptosis
XIAP	⊣	Apoptosis
cFLIP	⊣	Apoptosis
ATP	→	Apoptosome
BAX	→	Apoptosome
BCL2	⊣	Apoptosome
CASP8	→	Apoptosome
Cyt_c	→	Apoptosome
Cyt_c&ATP	→	Apoptosome
DISC-FAS	→	Apoptosome
IKK	→	Apoptosome
MOMP	→	Apoptosome
MPT	⊣	Apoptosome
NFkB	⊣	Apoptosome
RIP1	⊣	Apoptosome
RIP1K	⊣	Apoptosome
RIP1ub	→	Apoptosome
TNFR	→	Apoptosome
XIAP	⊣	Apoptosome
cFLIP	⊣	Apoptosome
BAX	⊣	Apoptosome
CASP3	⊣	Apoptosome
NonACD	⊣	Apoptosome
cIAP	→	Apoptosome
Apoptosome	→	BAX
BCL2	⊣	BAX
CASP3	→	BAX
CASP8	→	BAX
Cyt_c	→	BAX
DISC-FAS	→	BAX
DISC-TNF	→	BAX
MOMP	→	BAX
MPT	⊣	BAX
RIP1K	⊣	BAX
ROS	⊣	BAX
TNF	→	BAX
TNFR	→	BAX
XIAP	⊣	BAX
cFLIP	⊣	BAX
BAX	→	BAX

NonACD	⊣	BAX
cIAP	→	BAX
DISC-FAS	→	BCL2
IKK	→	BCL2
NFkB	→	BCL2
RIP1	→	BCL2
RIP1ub	→	BCL2
TNF	→	BCL2
TNFR	→	BCL2
FASL	⊣	BCL2
XIAP	⊣	BCL2
cFLIP	→	BCL2
ATP	→	CASP3
Apoptosome	→	CASP3
BAX	→	CASP3
BCL2	⊣	CASP3
DISC-FAS	→	CASP3
FASL	→	CASP3
IKK	→	CASP3
MPT	⊣	CASP3
NFkB	⊣	CASP3
RIP1K	⊣	CASP3
RIP1ub	→	CASP3
ROS	⊣	CASP3
TNF	→	CASP3
cFLIP	⊣	CASP3
MOMP	⊣	CASP3
Survival	→	CASP3
TNF	⊣	CASP3
Apoptosome	→	CASP8
DISC-FAS	→	CASP8
DISC-TNF	→	CASP8
FASL	→	CASP8
IKK	→	CASP8
MPT	⊣	CASP8
RIP1	⊣	CASP8
RIP1K	⊣	CASP8
RIP1ub	→	CASP8
TNF	→	CASP8
TNFR	→	CASP8
XIAP	⊣	CASP8
cFLIP	⊣	CASP8
Apoptosis	⊣	CASP8
BCL2	→	CASP8
ATP	→	Cyt_c
BCL2	⊣	Cyt_c
CASP3	→	Cyt_c
CASP8	→	Cyt_c
DISC-FAS	→	Cyt_c
MPT	→	Cyt_c
RIP1K	→	Cyt_c
RIP1ub	⊣	Cyt_c
TNFR	→	Cyt_c
XIAP	⊣	Cyt_c
cFLIP	⊣	Cyt_c
Apoptosis	→	Cyt_c
RIP1ub	→	Cyt_c
Cyt_c	⊣	DISC-FAS
IKK	→	DISC-FAS
MOMP	→	DISC-FAS
MOMP	⊣	DISC-FAS
MPT	→	DISC-FAS
TNF	→	DISC-FAS
TNFR	→	DISC-FAS
TNF	→	DISC-TNF
TNFR	→	DISC-TNF

FASL	→	DISC-TNF
ATP	⊣	FASL
Apoptosome	⊣	FASL
Cyt_c	⊣	FASL
NFkB	→	FASL
RIP1	→	FASL
SMAC	→	FASL
SMAC	⊣	FASL
Survival	→	FASL
TNF	⊣	FASL
XIAP	→	FASL
DISC-FAS	→	IKK
FASL	→	IKK
RIP1ub	→	IKK
TNF	→	IKK
TNFR	→	IKK
BAX	⊣	IKK
BCL2	⊣	IKK
Cyt_c	→	IKK
NFkB	⊣	IKK
RIP1K	⊣	IKK
ATP	→	MOMP
CASP8	→	MOMP
Cyt_c	→	MOMP
DISC-FAS	→	MOMP
FASL	→	MOMP
IKK	⊣	MOMP
MPT	→	MOMP
RIP1	→	MOMP
RIP1K	→	MOMP
RIP1ub	⊣	MOMP
TNF	→	MOMP
TNFR	→	MOMP
XIAP	⊣	MOMP
cFLIP	⊣	MOMP
Apoptosis	→	MOMP
IKK	→	MOMP
NonACD	→	MOMP
NonACD	⊣	MOMP
XIAP	→	MOMP
BCL2	⊣	MPT
FASL	→	MPT
IKK	⊣	MPT
NFkB	⊣	MPT
RIP1	→	MPT
RIP1K	→	MPT
RIP1ub	⊣	MPT
ROS	→	MPT
TNF	→	MPT
TNFR	→	MPT
Apoptosome	→	MPT
BAX	→	MPT
CASP3	→	MPT
ROS	⊣	MPT
CASP3	⊣	NFkB
DISC-FAS	→	NFkB
FASL	→	NFkB
RIP1	→	NFkB
TNF	→	NFkB
Apoptosome	→	NFkB
Apoptosome	⊣	NFkB
TNFR	⊣	NFkB
ATP	⊣	NonACD
BCL2	⊣	NonACD
DISC-FAS	→	NonACD
FASL	→	NonACD

NFkB	$\dashv$	NonACD
RIP1ub	$\dashv$	NonACD
ROS	$\rightarrow$	NonACD
TNF	$\rightarrow$	NonACD
FASL	$\dashv$	NonACD
CASP8	$\dashv$	RIP1
DISC-FAS	$\rightarrow$	RIP1
FASL	$\rightarrow$	RIP1
TNF	$\rightarrow$	RIP1
TNFR	$\rightarrow$	RIP1
MOMP	$\rightarrow$	RIP1
NonACD	$\dashv$	RIP1
FASL	$\rightarrow$	RIP1K
RIP1	$\rightarrow$	RIP1K
TNF	$\rightarrow$	RIP1K
TNFR	$\rightarrow$	RIP1K
CASP8	$\rightarrow$	RIP1K
MPT	$\rightarrow$	RIP1K
SMAC	$\rightarrow$	RIP1K
cFLIP	$\dashv$	RIP1K
DISC-FAS	$\rightarrow$	RIP1ub
FASL	$\rightarrow$	RIP1ub
TNF	$\rightarrow$	RIP1ub
TNFR	$\rightarrow$	RIP1ub
cIAP&RIP1	$\rightarrow$	RIP1ub
Apoptosome	$\dashv$	RIP1ub
Cyt_c	$\dashv$	RIP1ub
IKK	$\rightarrow$	RIP1ub
NonACD	$\rightarrow$	RIP1ub
cIAP	$\dashv$	RIP1ub
BCL2	$\dashv$	ROS
DISC-FAS	$\rightarrow$	ROS
FASL	$\rightarrow$	ROS
IKK	$\dashv$	ROS
MPT	$\rightarrow$	ROS
RIP1K	$\rightarrow$	ROS
RIP1ub	$\dashv$	ROS
TNFR	$\rightarrow$	ROS
CASP3	$\rightarrow$	ROS
Apoptosome	$\rightarrow$	SMAC
BCL2	$\dashv$	SMAC
CASP3	$\rightarrow$	SMAC
Cyt_c	$\rightarrow$	SMAC
DISC-FAS	$\rightarrow$	SMAC
IKK	$\dashv$	SMAC
MPT	$\rightarrow$	SMAC
NFkB	$\dashv$	SMAC
RIP1	$\rightarrow$	SMAC
RIP1K	$\rightarrow$	SMAC
TNF	$\rightarrow$	SMAC
TNFR	$\rightarrow$	SMAC
XIAP	$\dashv$	SMAC
cFLIP	$\dashv$	SMAC
DISC-FAS	$\rightarrow$	Survival
FASL	$\rightarrow$	Survival
NFkB	$\rightarrow$	Survival
RIP1ub	$\rightarrow$	Survival
TNFR	$\rightarrow$	Survival
Apoptosome	$\rightarrow$	Survival
Cyt_c	$\dashv$	Survival
DISC-TNF	$\rightarrow$	Survival
IKK	$\dashv$	Survival
NonACD	$\rightarrow$	Survival
ROS	$\rightarrow$	Survival
TNF	$\dashv$	Survival
cFLIP	$\dashv$	Survival

ATP	⊣	TNF
Cyt_c	→	TNF
Cyt_c	⊣	TNF
RIP1ub	→	TNF
Survival	→	TNF
TNF	→	TNFR
RIP1K	→	TNFR
TNFR	⊣	TNFR
cIAP	→	TNFR
DISC-FAS	→	XIAP
FASL	→	XIAP
IKK	→	XIAP
SMAC	⊣	XIAP
TNF	→	XIAP
Cyt_c	→	XIAP
ROS	→	XIAP
DISC-FAS	→	cFLIP
IKK	→	cFLIP
RIP1ub	→	cFLIP
TNF	→	cFLIP
TNFR	→	cFLIP
MPT	→	cFLIP
XIAP	→	cFLIP
cIAP	⊣	cFLIP
Apoptosome	⊣	cIAP
BAX	⊣	cIAP
CASP3	⊣	cIAP
CASP8	⊣	cIAP
Cyt_c	⊣	cIAP
DISC-TNF	⊣	cIAP
FASL	⊣	cIAP
IKK	→	cIAP
MOMP	⊣	cIAP
MPT	⊣	cIAP
RIP1K	⊣	cIAP
RIP1ub	→	cIAP
ROS	⊣	cIAP
SMAC	⊣	cIAP
TNFR	⊣	cIAP
cIAP	→	cIAP
MOMP	→	cIAP
NonACD	⊣	cIAP

Cell-fate decision model: in-silico PKN 5 (40% noise)

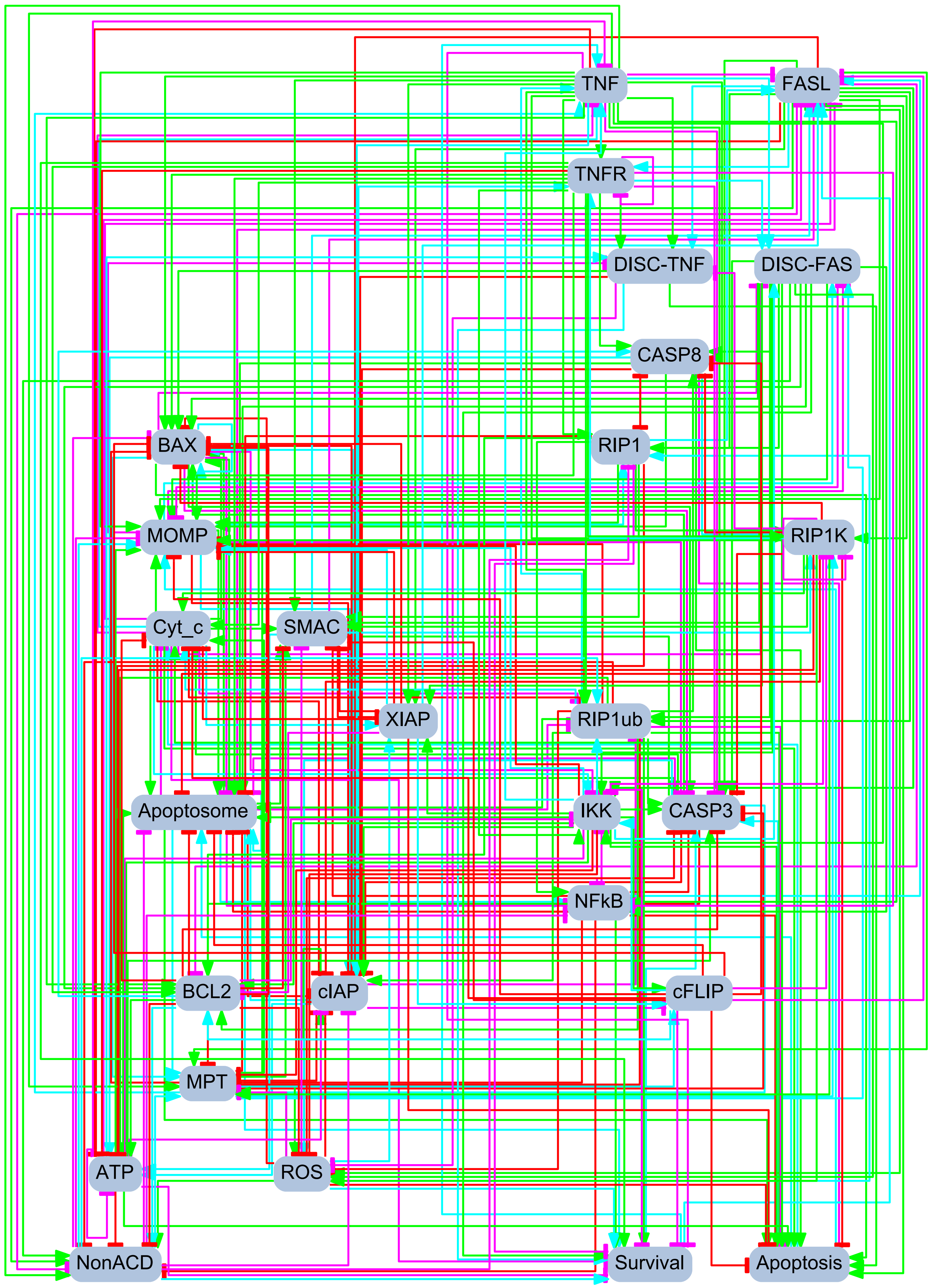

BCL2	→	ATP
FASL	⊣	ATP
IKK	→	ATP
RIP1	⊣	ATP
RIP1K	⊣	ATP
RIP1ub	→	ATP
TNF	⊣	ATP
TNFR	⊣	ATP
ATP	⊣	ATP
CASP8	→	ATP
IKK	⊣	ATP
SMAC	→	ATP
cIAP	→	ATP
ATP	→	Apoptosis
Apoptosome	→	Apoptosis
BAX	→	Apoptosis
CASP8	→	Apoptosis
Cyt_c	→	Apoptosis
DISC-FAS	→	Apoptosis
DISC-TNF	→	Apoptosis
FASL	→	Apoptosis
IKK	→	Apoptosis
NFkB	⊣	Apoptosis
RIP1K	⊣	Apoptosis
RIP1ub	→	Apoptosis
ROS	⊣	Apoptosis
XIAP	⊣	Apoptosis
cFLIP	⊣	Apoptosis
RIP1ub	⊣	Apoptosis
ATP	→	Apoptosome
BAX	→	Apoptosome
BCL2	⊣	Apoptosome
CASP8	→	Apoptosome
Cyt_c	→	Apoptosome
DISC-FAS	→	Apoptosome
IKK	→	Apoptosome
MOMP	→	Apoptosome
MPT	⊣	Apoptosome
NFkB	⊣	Apoptosome
RIP1	⊣	Apoptosome
RIP1K	⊣	Apoptosome
RIP1ub	→	Apoptosome
TNFR	→	Apoptosome
cFLIP	⊣	Apoptosome
Apoptosis	→	Apoptosome
Apoptosome	→	Apoptosome
BAX	⊣	Apoptosome
CASP3	⊣	Apoptosome
NonACD	⊣	Apoptosome
cIAP	→	Apoptosome
Apoptosome	→	BAX
BCL2	⊣	BAX
CASP3	→	BAX
CASP8	→	BAX
Cyt_c	→	BAX
DISC-FAS	→	BAX
DISC-TNF	→	BAX
MPT	⊣	BAX
RIP1K	⊣	BAX
ROS	⊣	BAX
TNF	→	BAX
TNFR	→	BAX
XIAP	⊣	BAX
cFLIP	⊣	BAX
BAX	→	BAX
NonACD	⊣	BAX

cIAP	→	BAX
DISC-FAS	→	BCL2
IKK	→	BCL2
NFkB	→	BCL2
RIP1	→	BCL2
RIP1ub	→	BCL2
TNF	→	BCL2
TNFR	→	BCL2
FASL	⊣	BCL2
XIAP	⊣	BCL2
cFLIP	→	BCL2
ATP	→	CASP3
Apoptosome	→	CASP3
BAX	→	CASP3
BCL2	⊣	CASP3
DISC-FAS	→	CASP3
FASL	→	CASP3
IKK	→	CASP3
MPT	⊣	CASP3
NFkB	⊣	CASP3
RIP1K	⊣	CASP3
RIP1ub	→	CASP3
ROS	⊣	CASP3
TNF	→	CASP3
Apoptosis	→	CASP3
BAX	⊣	CASP3
MOMP	⊣	CASP3
Survival	→	CASP3
TNF	⊣	CASP3
TNFR	⊣	CASP3
DISC-FAS	→	CASP8
RIP1	⊣	CASP8
RIP1K	⊣	CASP8
RIP1ub	→	CASP8
TNF	→	CASP8
TNFR	→	CASP8
cFLIP	⊣	CASP8
Apoptosis	⊣	CASP8
BCL2	→	CASP8
BCL2	⊣	Cyt_c
CASP3	→	Cyt_c
DISC-FAS	→	Cyt_c
MPT	→	Cyt_c
RIP1K	→	Cyt_c
RIP1ub	⊣	Cyt_c
TNFR	→	Cyt_c
XIAP	⊣	Cyt_c
cFLIP	⊣	Cyt_c
Apoptosis	→	Cyt_c
Apoptosome	⊣	Cyt_c
RIP1ub	→	Cyt_c
BAX	⊣	DISC-FAS
Cyt_c	⊣	DISC-FAS
IKK	→	DISC-FAS
MOMP	→	DISC-FAS
MOMP	⊣	DISC-FAS
MPT	→	DISC-FAS
TNF	→	DISC-FAS
TNFR	→	DISC-FAS
TNF	→	DISC-TNF
TNFR	→	DISC-TNF
ATP	→	DISC-TNF
FASL	→	DISC-TNF
MOMP	⊣	DISC-TNF
RIP1K	⊣	DISC-TNF
ATP	⊣	FASL

Apoptosome	$\dashv$	FASL
Cyt_c	$\dashv$	FASL
NFkB	$\to$	FASL
RIP1	$\to$	FASL
SMAC	$\to$	FASL
SMAC	$\dashv$	FASL
Survival	$\to$	FASL
TNF	$\dashv$	FASL
XIAP	$\to$	FASL
cFLIP	$\dashv$	FASL
DISC-FAS	$\to$	IKK
FASL	$\to$	IKK
TNF	$\to$	IKK
TNFR	$\to$	IKK
BAX	$\dashv$	IKK
BCL2	$\dashv$	IKK
Cyt_c	$\to$	IKK
NFkB	$\dashv$	IKK
RIP1K	$\dashv$	IKK
cFLIP	$\to$	IKK
ATP	$\to$	MOMP
CASP8	$\to$	MOMP
Cyt_c	$\to$	MOMP
DISC-FAS	$\to$	MOMP
FASL	$\to$	MOMP
IKK	$\dashv$	MOMP
MPT	$\to$	MOMP
RIP1	$\to$	MOMP
RIP1K	$\to$	MOMP
RIP1ub	$\dashv$	MOMP
TNF	$\to$	MOMP
TNFR	$\to$	MOMP
XIAP	$\dashv$	MOMP
cFLIP	$\dashv$	MOMP
Apoptosis	$\to$	MOMP
DISC-FAS	$\dashv$	MOMP
IKK	$\to$	MOMP
NonACD	$\to$	MOMP
NonACD	$\dashv$	MOMP
XIAP	$\to$	MOMP
BCL2	$\dashv$	MPT
FASL	$\to$	MPT
IKK	$\dashv$	MPT
NFkB	$\dashv$	MPT
RIP1K	$\to$	MPT
RIP1ub	$\dashv$	MPT
ROS	$\to$	MPT
TNF	$\to$	MPT
Apoptosome	$\to$	MPT
BAX	$\to$	MPT
CASP3	$\to$	MPT
NonACD	$\to$	MPT
ROS	$\dashv$	MPT
CASP3	$\dashv$	NFkB
DISC-FAS	$\to$	NFkB
RIP1	$\to$	NFkB
TNF	$\to$	NFkB
Apoptosome	$\to$	NFkB
Apoptosome	$\dashv$	NFkB
IKK	$\dashv$	NFkB
NonACD	$\dashv$	NFkB
TNFR	$\dashv$	NFkB
ATP	$\dashv$	NonACD
BCL2	$\dashv$	NonACD
DISC-FAS	$\to$	NonACD
FASL	$\to$	NonACD

NFkB	$\dashv$	NonACD
RIP1ub	$\dashv$	NonACD
ROS	$\to$	NonACD
TNF	$\to$	NonACD
BCL2	$\to$	NonACD
FASL	$\dashv$	NonACD
CASP8	$\dashv$	RIP1
DISC-FAS	$\to$	RIP1
FASL	$\to$	RIP1
TNF	$\to$	RIP1
MOMP	$\to$	RIP1
NonACD	$\dashv$	RIP1
ROS	$\to$	RIP1
FASL	$\to$	RIP1K
RIP1	$\to$	RIP1K
TNF	$\to$	RIP1K
TNFR	$\to$	RIP1K
CASP8	$\to$	RIP1K
MPT	$\to$	RIP1K
RIP1K	$\dashv$	RIP1K
SMAC	$\to$	RIP1K
cFLIP	$\dashv$	RIP1K
DISC-FAS	$\to$	RIP1ub
FASL	$\to$	RIP1ub
TNF	$\to$	RIP1ub
TNFR	$\to$	RIP1ub
Apoptosome	$\dashv$	RIP1ub
Cyt_c	$\dashv$	RIP1ub
IKK	$\to$	RIP1ub
NonACD	$\to$	RIP1ub
cIAP	$\dashv$	RIP1ub
BCL2	$\dashv$	ROS
DISC-FAS	$\to$	ROS
FASL	$\to$	ROS
IKK	$\dashv$	ROS
MPT	$\to$	ROS
RIP1K	$\to$	ROS
RIP1ub	$\dashv$	ROS
CASP3	$\to$	ROS
DISC-TNF	$\dashv$	ROS
Apoptosome	$\to$	SMAC
BCL2	$\dashv$	SMAC
CASP3	$\to$	SMAC
Cyt_c	$\to$	SMAC
DISC-FAS	$\to$	SMAC
MPT	$\to$	SMAC
NFkB	$\dashv$	SMAC
RIP1	$\to$	SMAC
RIP1K	$\to$	SMAC
TNF	$\to$	SMAC
XIAP	$\dashv$	SMAC
cFLIP	$\dashv$	SMAC
ROS	$\dashv$	SMAC
DISC-FAS	$\to$	Survival
NFkB	$\to$	Survival
RIP1ub	$\to$	Survival
TNFR	$\to$	Survival
ATP	$\dashv$	Survival
Apoptosome	$\to$	Survival
Cyt_c	$\dashv$	Survival
DISC-TNF	$\to$	Survival
IKK	$\dashv$	Survival
NonACD	$\to$	Survival
RIP1	$\dashv$	Survival
ROS	$\to$	Survival
TNF	$\dashv$	Survival

cFLIP	⊣	Survival
ATP	⊣	TNF
Cyt_c	→	TNF
Cyt_c	⊣	TNF
IKK	→	TNF
MPT	→	TNF
RIP1ub	→	TNF
Survival	→	TNF
TNF	→	TNFR
FASL	→	TNFR
RIP1K	→	TNFR
TNFR	⊣	TNFR
cIAP	→	TNFR
DISC-FAS	→	XIAP
FASL	→	XIAP
IKK	→	XIAP
SMAC	⊣	XIAP
TNF	→	XIAP
Cyt_c	→	XIAP
ROS	→	XIAP
IKK	→	cFLIP
MPT	→	cFLIP
XIAP	→	cFLIP
cIAP	⊣	cFLIP
Apoptosome	⊣	cIAP
BAX	⊣	cIAP
CASP3	⊣	cIAP
CASP8	⊣	cIAP
Cyt_c	⊣	cIAP
DISC-TNF	⊣	cIAP
FASL	⊣	cIAP
IKK	→	cIAP
MOMP	⊣	cIAP
MPT	⊣	cIAP
RIP1K	⊣	cIAP
RIP1ub	→	cIAP
ROS	⊣	cIAP
cIAP	→	cIAP
ATP	⊣	cIAP
MOMP	→	cIAP
NonACD	⊣	cIAP
TNF	→	cIAP

Cell-fate decision model: in-silico PKN 6 (50% noise)

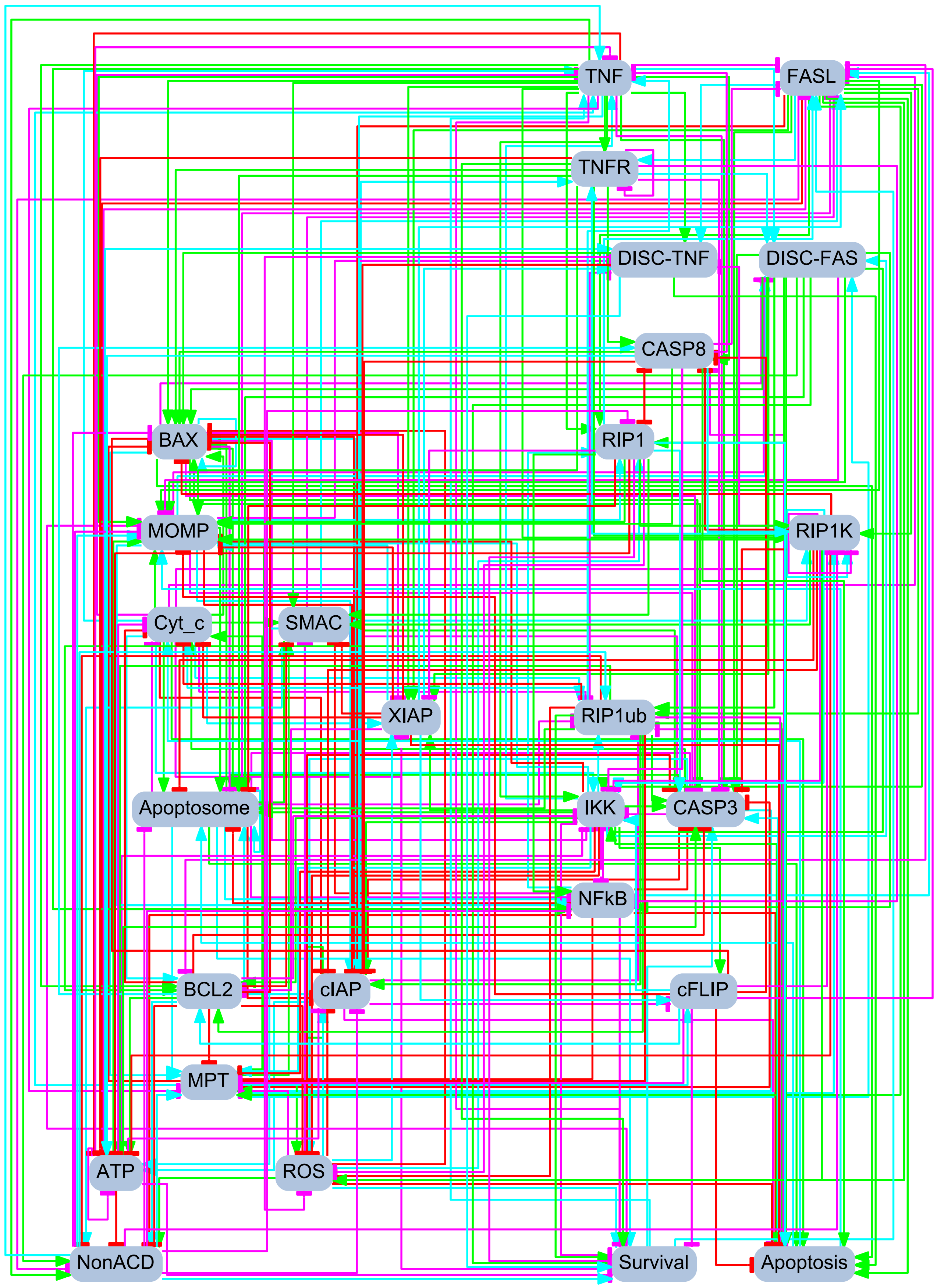

BCL2	→	ATP
FASL	⊣	ATP
RIP1	⊣	ATP
RIP1K	⊣	ATP
RIP1ub	→	ATP
TNF	⊣	ATP
TNFR	⊣	ATP
ATP	⊣	ATP
CASP8	→	ATP
IKK	⊣	ATP
SMAC	→	ATP
cIAP	→	ATP
Apoptosome	→	Apoptosis
BAX	→	Apoptosis
CASP8	→	Apoptosis
Cyt_c	→	Apoptosis
DISC-FAS	→	Apoptosis
DISC-TNF	→	Apoptosis
FASL	→	Apoptosis
IKK	→	Apoptosis
NFkB	⊣	Apoptosis
RIP1ub	→	Apoptosis
ROS	⊣	Apoptosis
XIAP	⊣	Apoptosis
cFLIP	⊣	Apoptosis
RIP1	→	Apoptosis
RIP1ub	⊣	Apoptosis
cIAP	⊣	Apoptosis
BAX	→	Apoptosome
Cyt_c	→	Apoptosome
DISC-FAS	→	Apoptosome
IKK	→	Apoptosome
MOMP	→	Apoptosome
NFkB	⊣	Apoptosome
RIP1	⊣	Apoptosome
RIP1K	⊣	Apoptosome
RIP1ub	→	Apoptosome
TNFR	→	Apoptosome
Apoptosis	→	Apoptosome
Apoptosome	→	Apoptosome
BAX	⊣	Apoptosome
CASP3	⊣	Apoptosome
NonACD	⊣	Apoptosome
cIAP	→	Apoptosome
Apoptosome	→	BAX
BCL2	⊣	BAX
CASP3	→	BAX
CASP8	→	BAX
Cyt_c	→	BAX
DISC-FAS	→	BAX
DISC-TNF	→	BAX
MPT	⊣	BAX
RIP1K	⊣	BAX
ROS	⊣	BAX
TNF	→	BAX
TNFR	→	BAX
XIAP	⊣	BAX
cFLIP	⊣	BAX
BAX	→	BAX
NonACD	⊣	BAX
cIAP	→	BAX
DISC-FAS	→	BCL2
IKK	→	BCL2
RIP1ub	→	BCL2
TNF	→	BCL2
Cyt_c	→	BCL2

FASL	⊣	BCL2
XIAP	⊣	BCL2
cFLIP	→	BCL2
ATP	→	CASP3
Apoptosome	→	CASP3
BAX	→	CASP3
BCL2	⊣	CASP3
DISC-FAS	→	CASP3
FASL	→	CASP3
IKK	→	CASP3
MPT	⊣	CASP3
NFkB	⊣	CASP3
RIP1K	⊣	CASP3
RIP1ub	→	CASP3
ROS	⊣	CASP3
TNF	→	CASP3
Apoptosis	→	CASP3
BAX	⊣	CASP3
MOMP	⊣	CASP3
RIP1	→	CASP3
Survival	→	CASP3
TNF	⊣	CASP3
TNFR	⊣	CASP3
RIP1	⊣	CASP8
RIP1K	⊣	CASP8
TNF	→	CASP8
TNFR	→	CASP8
cFLIP	⊣	CASP8
Apoptosis	⊣	CASP8
BCL2	→	CASP8
BCL2	⊣	Cyt_c
CASP3	→	Cyt_c
MPT	→	Cyt_c
RIP1ub	⊣	Cyt_c
XIAP	⊣	Cyt_c
ATP	⊣	Cyt_c
Apoptosis	→	Cyt_c
Apoptosome	⊣	Cyt_c
RIP1ub	→	Cyt_c
BAX	⊣	DISC-FAS
Cyt_c	⊣	DISC-FAS
IKK	→	DISC-FAS
MOMP	→	DISC-FAS
MOMP	⊣	DISC-FAS
MPT	→	DISC-FAS
TNF	→	DISC-FAS
TNFR	→	DISC-FAS
TNF	→	DISC-TNF
ATP	→	DISC-TNF
FASL	→	DISC-TNF
MOMP	⊣	DISC-TNF
RIP1K	⊣	DISC-TNF
RIP1ub	⊣	DISC-TNF
XIAP	→	DISC-TNF
ATP	⊣	FASL
Apoptosome	⊣	FASL
CASP8	⊣	FASL
Cyt_c	⊣	FASL
NFkB	→	FASL
RIP1	→	FASL
SMAC	→	FASL
SMAC	⊣	FASL
Survival	→	FASL
TNF	⊣	FASL
XIAP	→	FASL
cFLIP	⊣	FASL

DISC-FAS	$\rightarrow$	IKK
FASL	$\rightarrow$	IKK
TNF	$\rightarrow$	IKK
BAX	$\dashv$	IKK
BCL2	$\dashv$	IKK
CASP3	$\dashv$	IKK
CASP8	$\dashv$	IKK
Cyt_c	$\rightarrow$	IKK
NFkB	$\dashv$	IKK
RIP1K	$\rightarrow$	IKK
RIP1K	$\dashv$	IKK
cFLIP	$\rightarrow$	IKK
ATP	$\rightarrow$	MOMP
CASP8	$\rightarrow$	MOMP
Cyt_c	$\rightarrow$	MOMP
DISC-FAS	$\rightarrow$	MOMP
FASL	$\rightarrow$	MOMP
IKK	$\dashv$	MOMP
MPT	$\rightarrow$	MOMP
RIP1	$\rightarrow$	MOMP
RIP1K	$\rightarrow$	MOMP
TNF	$\rightarrow$	MOMP
TNFR	$\rightarrow$	MOMP
XIAP	$\dashv$	MOMP
cFLIP	$\dashv$	MOMP
Apoptosis	$\rightarrow$	MOMP
DISC-FAS	$\dashv$	MOMP
IKK	$\rightarrow$	MOMP
NonACD	$\rightarrow$	MOMP
NonACD	$\dashv$	MOMP
Survival	$\dashv$	MOMP
XIAP	$\rightarrow$	MOMP
BCL2	$\dashv$	MPT
FASL	$\rightarrow$	MPT
IKK	$\dashv$	MPT
NFkB	$\dashv$	MPT
RIP1ub	$\dashv$	MPT
ROS	$\rightarrow$	MPT
Apoptosome	$\rightarrow$	MPT
BAX	$\rightarrow$	MPT
CASP3	$\rightarrow$	MPT
IKK	$\rightarrow$	MPT
NonACD	$\rightarrow$	MPT
ROS	$\dashv$	MPT
TNF	$\dashv$	MPT
cFLIP	$\dashv$	MPT
CASP3	$\dashv$	NFkB
DISC-FAS	$\rightarrow$	NFkB
RIP1	$\rightarrow$	NFkB
TNF	$\rightarrow$	NFkB
Apoptosome	$\rightarrow$	NFkB
Apoptosome	$\dashv$	NFkB
IKK	$\dashv$	NFkB
MOMP	$\rightarrow$	NFkB
NonACD	$\dashv$	NFkB
TNFR	$\dashv$	NFkB
ATP	$\dashv$	NonACD
BCL2	$\dashv$	NonACD
DISC-FAS	$\rightarrow$	NonACD
NFkB	$\dashv$	NonACD
RIP1ub	$\dashv$	NonACD
ROS	$\rightarrow$	NonACD
TNF	$\rightarrow$	NonACD
BCL2	$\rightarrow$	NonACD
FASL	$\dashv$	NonACD
SMAC	$\rightarrow$	NonACD

CASP8	$\dashv$	RIP1
DISC-FAS	$\rightarrow$	RIP1
FASL	$\rightarrow$	RIP1
TNF	$\rightarrow$	RIP1
MOMP	$\rightarrow$	RIP1
NFkB	$\rightarrow$	RIP1
NonACD	$\dashv$	RIP1
ROS	$\rightarrow$	RIP1
FASL	$\rightarrow$	RIP1K
RIP1	$\rightarrow$	RIP1K
TNF	$\rightarrow$	RIP1K
TNFR	$\rightarrow$	RIP1K
CASP8	$\rightarrow$	RIP1K
MPT	$\rightarrow$	RIP1K
NonACD	$\dashv$	RIP1K
RIP1K	$\rightarrow$	RIP1K
RIP1K	$\dashv$	RIP1K
SMAC	$\rightarrow$	RIP1K
cFLIP	$\dashv$	RIP1K
DISC-FAS	$\rightarrow$	RIP1ub
FASL	$\rightarrow$	RIP1ub
Apoptosis	$\dashv$	RIP1ub
Apoptosome	$\dashv$	RIP1ub
Cyt_c	$\rightarrow$	RIP1ub
Cyt_c	$\dashv$	RIP1ub
IKK	$\rightarrow$	RIP1ub
NonACD	$\rightarrow$	RIP1ub
cIAP	$\dashv$	RIP1ub
BCL2	$\dashv$	ROS
FASL	$\rightarrow$	ROS
IKK	$\dashv$	ROS
MPT	$\rightarrow$	ROS
RIP1K	$\rightarrow$	ROS
RIP1ub	$\dashv$	ROS
CASP3	$\rightarrow$	ROS
DISC-TNF	$\dashv$	ROS
RIP1	$\dashv$	ROS
Apoptosome	$\rightarrow$	SMAC
BCL2	$\dashv$	SMAC
CASP3	$\rightarrow$	SMAC
Cyt_c	$\rightarrow$	SMAC
MPT	$\rightarrow$	SMAC
NFkB	$\dashv$	SMAC
RIP1	$\rightarrow$	SMAC
RIP1K	$\rightarrow$	SMAC
TNF	$\rightarrow$	SMAC
XIAP	$\dashv$	SMAC
CASP3	$\dashv$	SMAC
ROS	$\dashv$	SMAC
DISC-FAS	$\rightarrow$	Survival
RIP1ub	$\rightarrow$	Survival
TNFR	$\rightarrow$	Survival
ATP	$\dashv$	Survival
Apoptosome	$\rightarrow$	Survival
Cyt_c	$\dashv$	Survival
DISC-TNF	$\rightarrow$	Survival
IKK	$\dashv$	Survival
NFkB	$\dashv$	Survival
NonACD	$\rightarrow$	Survival
RIP1	$\dashv$	Survival
ROS	$\rightarrow$	Survival
TNF	$\dashv$	Survival
cFLIP	$\dashv$	Survival
ATP	$\dashv$	TNF
CASP8	$\dashv$	TNF
Cyt_c	$\rightarrow$	TNF

Cyt_c	⊣	TNF
IKK	→	TNF
MPT	→	TNF
NonACD	→	TNF
RIP1ub	→	TNF
Survival	→	TNF
TNF	→	TNFR
FASL	→	TNFR
RIP1K	→	TNFR
TNFR	⊣	TNFR
cIAP	→	TNFR
DISC-FAS	→	XIAP
FASL	→	XIAP
IKK	→	XIAP
TNF	→	XIAP
BAX	⊣	XIAP
Cyt_c	→	XIAP
NFkB	⊣	XIAP
RIP1	⊣	XIAP
ROS	→	XIAP
IKK	→	cFLIP
MPT	→	cFLIP
XIAP	→	cFLIP
cIAP	⊣	cFLIP
Apoptosome	⊣	cIAP
BAX	⊣	cIAP
CASP3	⊣	cIAP
CASP8	⊣	cIAP
Cyt_c	⊣	cIAP
DISC-TNF	⊣	cIAP
FASL	⊣	cIAP
IKK	→	cIAP
MOMP	⊣	cIAP
RIP1K	⊣	cIAP
RIP1ub	→	cIAP
ROS	⊣	cIAP
cIAP	→	cIAP
ATP	⊣	cIAP
MOMP	→	cIAP
NonACD	⊣	cIAP
ROS	→	cIAP
TNF	→	cIAP
