# Supplementary material for: Boolean regulatory network reconstruction using literature based knowledge with a genetic algorithm optimization method
Source: BMC Bioinformatics. 2016 Oct 6;17:410. doi: 10.1186/s12859-016-1287-z (PMC5053080; doi:10.1186/s12859-016-1287-z)
Supplement: Additional file 5: — Comparison with related methods. Runtime comparison between our implementation of the method (optimusqual) and related methods. (PDF 1190 kb) [file 12859_2016_1287_MOESM5_ESM.pdf]

Comparison with related methods

Julien Dorier*[1] , Isaac Crespo[1] , Anne Niknejad[1] , Robin Liechti[1] , Martin Ebeling[2] and Ioannis Xenarios*[1]

[1]Vital-IT, SIB Swiss Institute of Bioinformatics, 1015 Lausanne, Switzerland

[2]Pharmaceutical Sciences / Translational Technologies and Bionformatics, Roche Innovation Center Basel, 124 Grenzacherstrasse, 4070 Basel, Switzerland

Email: Julien Dorier *- julien.dorier@isb-sib.ch; Isaac Crespo - isaac.crespo@isb-sib.ch; Anne Niknejad - Anne.Niknejad@isb-sib.ch; Robin Liechti - Robin.Liechti@isb-sib.ch; Martin Ebeling - martin.ebeling@roche.com; Ioannis Xenarios *- ioannis.xenarios@isb-sib.ch;

*Corresponding author

In the following we compare the performance of optimusqual (our implementation of the network optimization method) with related software that use a training set based on stable phenotypes (like optimusqual), and for which a fair comparison is possible: lpNet [1, 2], XPRED [3] and PRUNET [4]. Before presenting and discussing the results of the comparison, we start by specifying the conditions and input files used for each software.

All comparisons were performed using PKN 1 (Additional file 3) and training set 1 (Additional file 4) as input. However, since lpNet, XPRED and PRUNET cannot use information on transitions between steady states, we generated a training set containing only the information on stable phenotypes (steady states). For optimusqual, the full training set 1 was used, including transitions between steady states.

*lpNet**

Training set 1 contains information on steady states obtained under various perturbations combining the simultaneous knock-out (node always set to 0) and/or over-expression (node always set to 1) of multiple nodes. Although lpNet understands the notion of perturbations, only node knock-out is implemented and node over-expression is not possible. To overcome this problem, self-feedback loops on each of the nodes that are over-expressed in training set 1 (TNF and FASL) were added to the input PKN. Since both TNF and FASL are input nodes (without incoming edges), this simple workaround allow the networks to sustain a continuous activation of these nodes, effectively mimicking over-expression. Contrarily to optimusqual, where prior knowledge consists in a list of allowed edges, lpNet uses prior knowledge in the form of a list of edges that are either mandatory or forbidden. To allow lpNet to use the prior knowledge given in PKN 1, we converted it to a list of forbidden edges formed by all possible edges that were not in PKN 1.

For a given set of input parameters, lpNet produced always the same network. Therefore, only one run of lpNet is needed once input parameters have been chosen. However, an important penalty parameter (λ) controlling the introduction of slack variables in the model must be specified. To determine the optimal value for this parameter, we used leave-one-out cross-validation†, as suggested in the lpNet documentation.

*version 2.2 with R 3.2.2.

†using loocv(), with delta=0.5, times=5, active_mu=0, inactive_mu=1, active_sd=0.01 and inactive_sd=0.01.

PRUNET

The notion of perturbation is not implemented in PRUNET. Therefore, to allow PRUNET to use all information contained in the training set, we had to modify the input PKN. To allow PRUNET to mimic over-expression of TNF and FASL, two self-feedback loops on TNF and FASL were added to the input PKN. To allow knock-out of CASP3, CASP8, NFkB, RIP1 and cIAP, dummy nodes were added to the PKN (CASP3_KO, CASP8_KO, NFkB_KO, RIP1_KO and cIAP_KO), each with a self-feedback loop and inhibiting the corresponding node:

$$\begin{array}{rcl} \text{CASP3\_KO} & \rightarrow & \text{CASP3\_KO} \\ \text{CASP3\_KO} & \dashv & \text{CASP3} \\ \text{CASP8\_KO} & \rightarrow & \text{CASP8\_KO} \\ \text{CASP8\_KO} & \dashv & \text{CASP8} \\ \text{NFkB\_KO} & \rightarrow & \text{NFkB\_KO} \\ \text{NFkB\_KO} & \dashv & \text{NFkB} \\ \text{RIP1\_KO} & \rightarrow & \text{RIP1\_KO} \\ \text{RIP1\_KO} & \dashv & \text{RIP1} \\ \text{cIAP\_KO} & \rightarrow & \text{cIAP\_KO} \\ \text{cIAP\_KO} & \dashv & \text{cIAP} \end{array}$$

This trick allows PRUNET to mimic the knock-out of one node (e.g. CASP3), by choosing a state with the corresponding KO dummy node in its active state (e.g. CASP3_KO=1). States of these dummy nodes were also added to the training set, with a state 1 for all phenotypes obtained with a knock-out of the corresponding node, and a state 0 in all other phenotypes.

Similarly to optimusqual, PRUNET is based on a evolutionary algorithms and require starting multiple runs and keeping only a fraction of the resulting networks (10% in this work) to increase the probability of finding good solutions. To evaluate PRUNET, we started 100 independent runs and kept the best network obtained with each run. Among these 100 resulting networks, only the 10 best networks (according to PRUNET internal scoring) were kept as model networks. For each run, we used a population of 85 networks (which corresponds to the maximum number of networks evaluated by optimusqual with 50 replicas) and 1000 iterations. This number of iteration is significantly larger than the number of iteration used in optimusqual (175 iterations in average, with standard deviation 45), but it was required to allow PRUNET to find sufficiently good solutions.

XPRED

We used almost the same inputs and settings as PRUNET. The only difference was the number of iterations that was decreased to 500. Using more iterations did not improve the convergence.

Optimusqual

We used the networks obtained in section “Network optimization: applied example” in the main text (50 models networks out of 500 runs).

Results and discussion

Running times

Table S1 presents a comparison of running times (wall time) measurements performed on an Intel Xeon X5550 @ 2.67GHz, 12Gb RAM, 64bits Linux OS (CentOS 7). First line of table S1 reports the average running time measured per run for each software. For lpNet, only cross-validation runs are reported. It is worth noting here that once the parameter λ is known, a network can be obtained with one run of lpNet without cross-validation with an average running time of 3.2 ± 0.7 minutes. For one run, and considering only cross-validation run for lpNet, optimusqual is about two order of magnitude faster than the other software with 10 minutes per run, while PRUNET, XPRED and lpNet take on average 36 hours, 64 hours and 48 hours respectively. However, one run is not sufficient to generate one network. For evolutionary algorithms

based methods (optimusqual, XPRED and PRUNET), we keep only the 10% best networks. About 10 runs are therefore required to produce one network. For lpNet, several runs of cross-validation are needed, each with a different value of the parameter λ, to determine its optimal value. For this specific problem, lpNet proposes a list of 94 values for λ. Based on these considerations, the second line of table S1 presents the average running time required to obtain one network. Our software optimusqual is the fastest, with less than 2 hours for 10 runs, while PRUNET and XPRED require about 15 days and 27 days respectively, and about 6 months are needed with lpNet. Given the large running time required by lpNet, testing all 94 values of λ proposed by lpNet is probably not necessary. A set of 10 different λ values may be sufficient to obtain an estimation of λ. In this case, the average running time taken by lpNet to obtain one network decreases to about 20 days.

Optimality

In addition to measuring its running time, it is important to check if a software can find optimal solutions, i.e. to check whether networks found by the software can reproduce the training set. To measure the difference between steady states of the networks and the training set, we used f_T (defined in Additional file 1) applied to the training set used as input for XPRED, PRUNET and lpNet (training set 1 without information on transition between steady states). With this definion, f_T ranges from 0 (best) to 1 (worst) and can be interpreted as the average difference (per node) between observations in the training set and steady states of the network. In particular, $f_T = 0$ can be only obtained if for all pairs of perturbation/observation (P, O) in the training set, the network perturbed with P has at least one steady state whose nodes states exactly correspond to the observation O.

A comparison of f_T measured on model networks obtained with all software is shown in Figure S3. Model networks obtained with optimusqual all have steady states that perfectly match the training set ($f_T \simeq 0$). The network obtained with lpNet is almost optimal, with $f_T \simeq 0.0087$, which corresponds to 6 out of 686 node states (49 steady states in training set, each with 14 nodes) that do not correspond to the observations contained in the training set. With a median $f_T \simeq 0.069$ and a best value of $f_T \simeq 0.048$, the model networks obtained with PRUNET are not as good as those obtained with optimusqual and lpNet. Finally, the model networks obtained with XPRED have the highest values of f_T with a median $f_T \simeq 0.089$ and a best value of $f_T \simeq 0.054$.

Predictive power

Figure S4 presents the distribution of s_{all} scores (defined in Additional file 1) of model networks as a function of the software used to generate the model networks. With a median $s_{all} \simeq 0.97$, optimusqual shows a significantly higher predictive power than than the networks obtained with lpNet ($s_{all} \simeq 0.92$), PRUNET (median $s_{all} \simeq 0.87$) and XPRED (median $s_{all} \simeq 0.85$).

Attractor reachability graphs

In contrast to other related methods, the training set used as input in optimusqual can include information not only on equilibrium properties (steady states), but also on the dynamical behaviour of the system (transitions between steady states upon perturbation). To illustrate the notion of dynamical behaviour, we considered the response to TNF perturbation starting from unperturbed network and evaluated the corresponding attractor reachability graphs for the gold standard network and the best network obtained with each software (according to the software). Figure S5 presents the attractor reachability graph obtained with the original gold-standard network. Note that part of the information contained in this graph is included in the training set: node states shown in blue are in the training set used as input for all software while transitions shown in blue were in training set used by optimusqual.

The attractor reachability graph for the best network obtained with optimusqual is shown in Figure S6. Interestingly, although no information on steady states 2, 3 and 4 of the unperturbed network was included in the training set, the attractor reachability graph of the best network obtained with optimusqual is almost

exactly correct, with only wrong node state in one steady state (in red, CASP8 in unperturbed steady state 4). In particular, it recovered the three additional unperturbed steady states (steady states 2, 3 and 4), as well as the correct transitions from these steady states to those obtained after TNF perturbation. Therefore, in this specific example, optimusqual was able to correctly recover not only the static properties (steady states), but also the dynamical behaviour of the original gold-standard model (transitions between steady states upon TNF perturbation).

The network obtained with lpNet recovers the steady states that were part of the training set (unperturbed steady state 1 and all steady states with TNF perturbation in Figure S7) with only one error (in red, CASP8 in unperturbed steady state 4). However, if fails to recover one steady state of the unperturbed network (steady state 2). More importantly, the dynamical behaviour is significantly different from the original gold standard model and several transitions are missing (dashed grey). In particular, this network predicts that starting from physiological condition (steady state 1, unperturbed network), TNF exposure will trigger only survival (Steady state 1 with TNF perturbation), while the original gold standard model predicts that TNF can trigger either survival (Survival=1, steady state 1 with TNF perturbation) or cell death by apoptosis (Apoptosis=1, steady state 2 with TNF perturbation) or by necrosis (NonACD=1, steady state 3 with TNF perturbation).

Figure S8 presents the attractor reachability graph for the best network obtained with PRUNET. All steady states are recovered, although with errors (shown in red). Several of these errors appear on nodes that are not in the training set (e.g. RIP1ub, TNFR, XIAP). Indeed, contrarily to optimusqual and lpNet, which try to reduce networks sizes during optimization, PRUNET generates model networks that contain all nodes appearing in the PKN, even if they are not in the training set. Another error is the presence of two spurious steady states, one in each condition (in red). The dynamical behaviour is also problematic: several transitions between steady states are missing (dashed grey), while others were added (red). In particular, starting from physiological condition (steady state 1, unperturbed network), this network predicts that TNF exposure will trigger either survival (Steady state 1 with TNF perturbation) or a state corresponding neither to survival, nor cell death (steady state 4 with TNF perturbation). This behaviour is not in agreement with the gold standard model behaviour (survival, apoptosis or necrosis).

The attractor reachability graph for the best network obtained with XPRED is shown in Figure S9. All steady states that were part of the training set (unperturbed steady state 1 and steady states 1, 2 and 3 with TNF perturbation) are recovered, although with errors (shown in red). Similarly to PRUNET, XPRED generates model networks that contain all nodes appearing in the PKN. As a consequence, a majority of errors are found on nodes that are not in the training set. In addition to these errors, one steady state of the unperturbed networks is not recovered (steady state 2), and a spurious steady state is predicted after TNF perturbation. Finally, the dynamical behaviour is significantly different from the original gold standard model, with several missing transitions (dashed grey). In particular, this network predicts only survival after TNF perturbation (starting from physiological condition), while the original gold standard model predicts that TNF can trigger either survival or cell death by apoptosis or by necrosis.

Conclusion

Contrarily to lpNet, XPRED and PRUNET, which all solve a similar optimization problem with a training set containing only information on steady states, optimusqual solves a different optimization problem with a training set that can contain not only information on steady states, but also on transitions between them upon perturbation. The use of transitions in the training set strongly increases the complexity of this optimization problem and prevents its reformulation as a linear optimization problem, as it is done in lpNet. However, thanks to the use of a heuristic algorithm, optimusqual is faster than all other tested software and finds better solutions, i.e. networks that can better reproduce the training set (as measured by f_T). More importantly, networks found by optimusqual have a higher predictive power (as measured by s_{all}). As illustrated with the response to TNF perturbation, networks found by optimusqual better reproduce both the equilibrium properties (steady states) and dynamical behaviour (transitions between steady states upon perturbation) of the underlying biological system.

References

1. Knapp B, Kaderali L: **Reconstruction of cellular signal transduction networks using perturbation assays and linear programming.** *PloS one* 2013, **8**(7):e69220.

2. Matos MRA, Knapp B, Kaderali L: **lpNet: a linear programming approach to reconstruct signal transduction networks**. *Bioinformatics* 2015, **31**(19):3231–3233.

3. Crespo I, Krishna A, Le Béchec A, del Sol A: **Predicting missing expression values in gene regulatory networks using a discrete logic modeling optimization guided by network stable states.** *Nucleic acids research* 2013, **41**:e8.

4. Rodriguez A, Crespo I, Androsova G, del Sol A: **Discrete Logic Modelling Optimization to Contextualize Prior Knowledge Networks Using PRUNET**. *PLOS ONE* 2015, **10**(6):e0127216.

	optimusqual	lpNet	PRUNET	XPRED
Time per run	10 ± 0.3	2910 ± 90	2160 ± 80	3860 ± 40
Time per network	100 ± 3	273540 ± 8000	21600 ± 1300	38600 ± 400

Table S1: Average running time (wall time) in minutes. For lpNet, time per run corresponds was measured on cross-validation runs. Time per network denote the time needed to obtain one network, i.e. 10 runs for methods based on evolutionary algorithms (optimusqual, XPRED and PRUNET), and 94 cross-validation runs for lpNet.

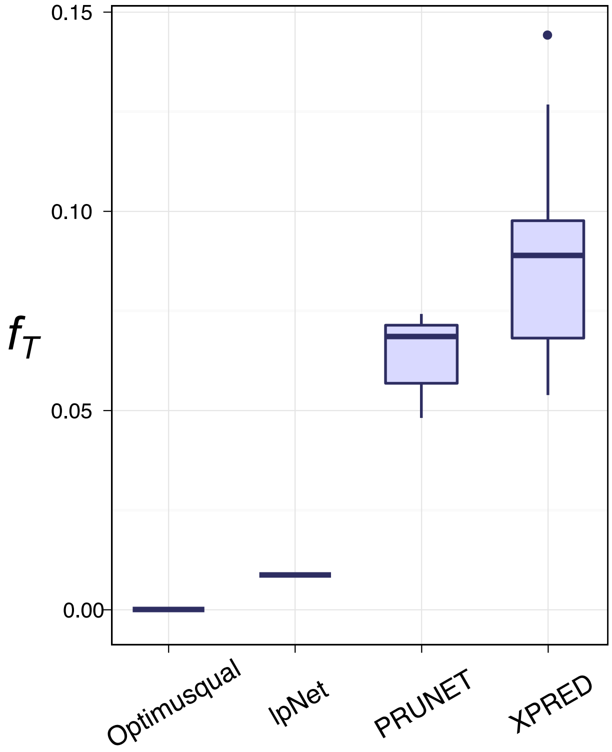

Figure S3: Comparison between steady states and training set measured by f_T for model networks obtained with all software (lower is better).

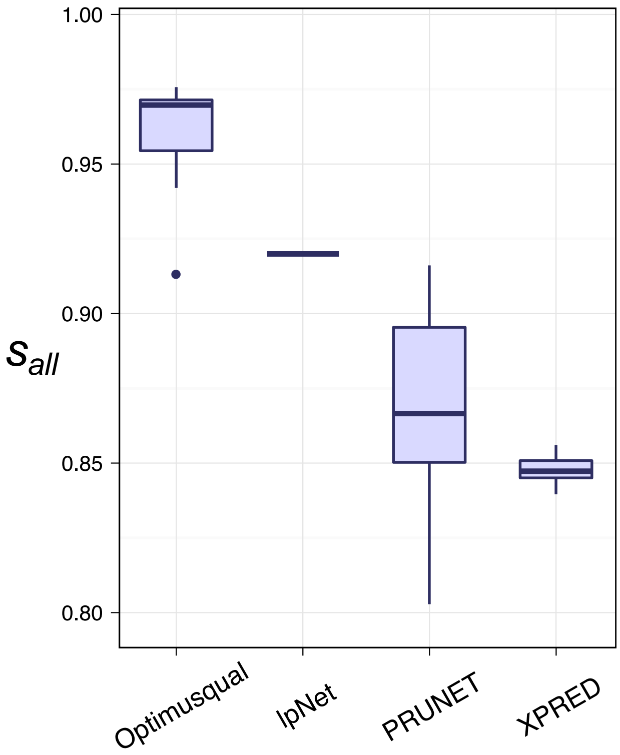

Figure S4: Predictive power s_{all} of model networks obtained with all software (higher is better).

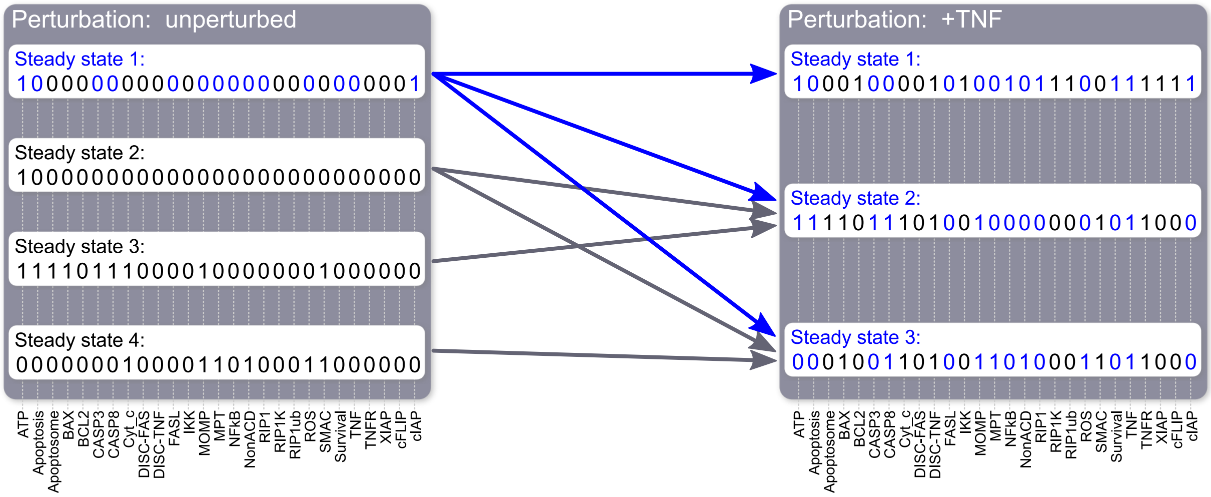

Figure S5: Attractor reachability graph of the gold-standard network for the response to TNF perturbation. States shown in blue are part of the training set used as input for all software. Transitions shown in blue are part of the training set used as input with optimusqual.

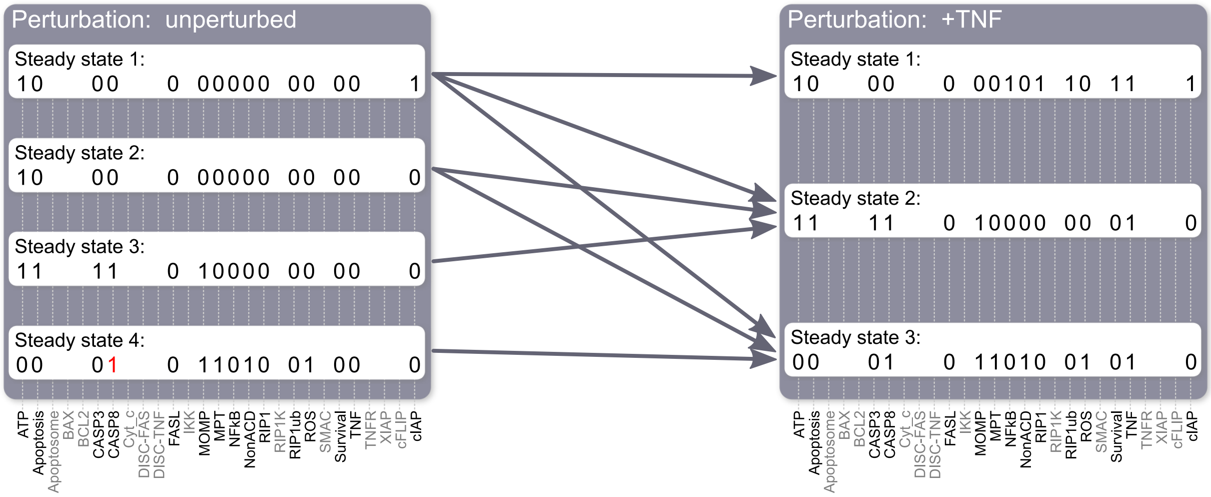

Figure S6: Attractor reachability graph of the best model network obtained with optimusqual. Errors in node states are shown in red. Missing node state values correspond to nodes of the gold-standard network that are not in the model network.

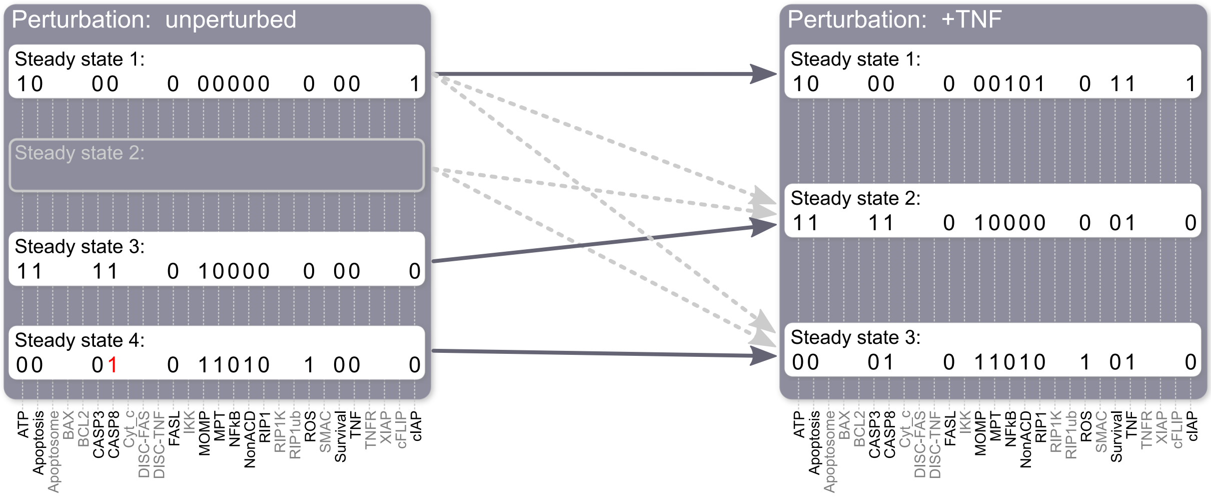

Figure S7: Attractor reachability graph of the model network obtained with lpNet. Errors in node states are shown in red. Missing transitions are shown in dashed grey. Missing steady state (steady state 2 of unperturbed network) is shown in light grey with empty background. Missing node state values correspond to nodes of the gold-standard network that are not in the model network.

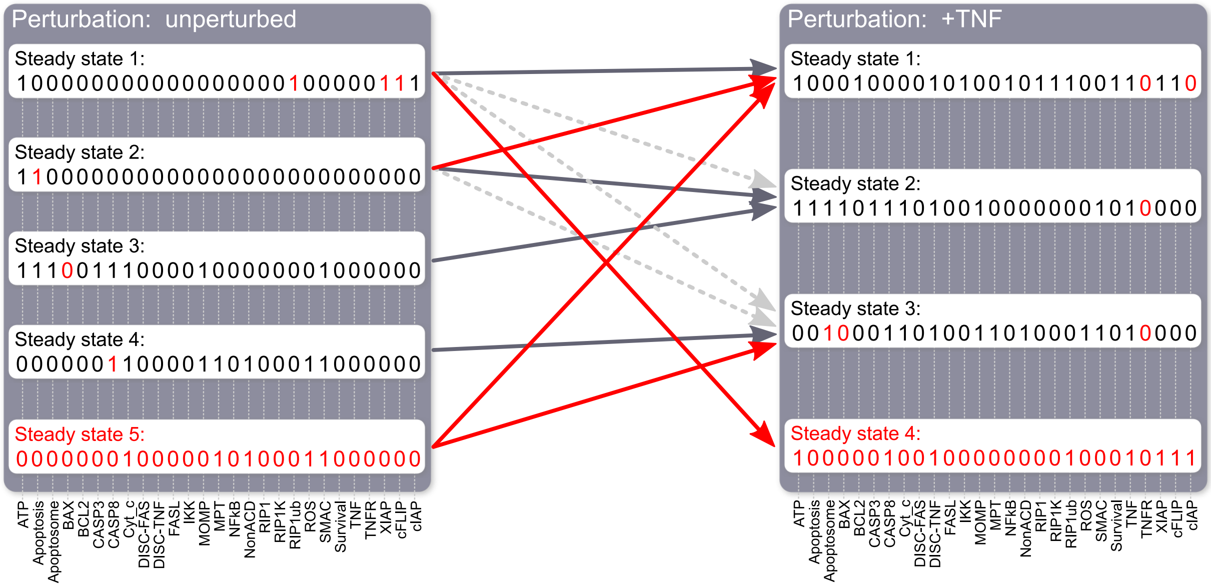

Figure S8: Attractor reachability graph of the best model network obtained with PRUNET. Errors in node states are shown in red. Missing transitions are shown in dashed grey. Additional steady states and transitions are shown in red.

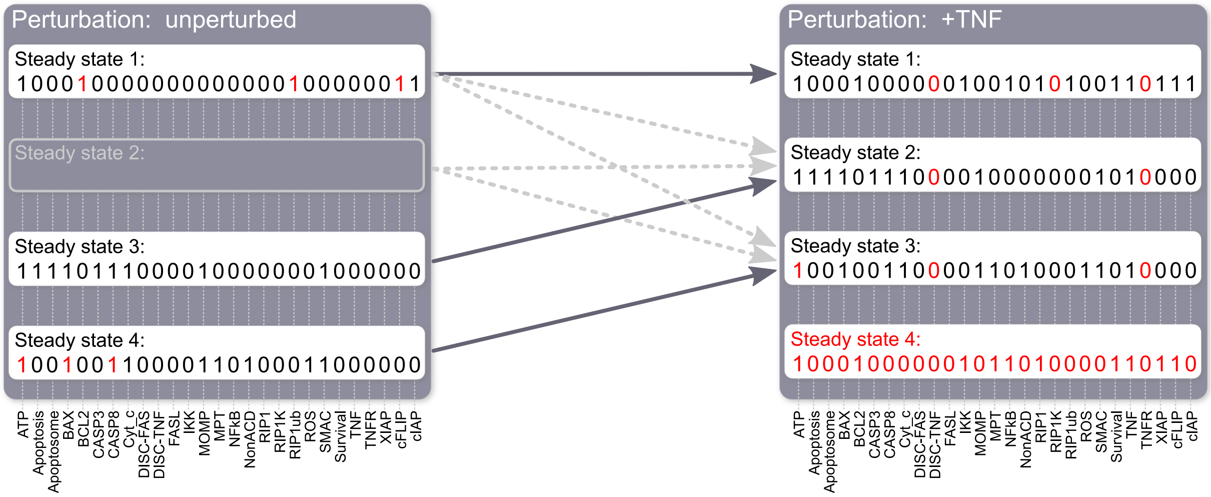

Figure S9: Attractor reachability graph of the best model network obtained with XPRED. Errors in node states are shown in red. Missing steady state (steady state 2 of unperturbed network) is shown in light grey with empty background. Missing transitions are shown in dashed grey. An additional steady state obtained with TNF perturbation (steady state 4) is shown in red.
